# RoBuster—Corpus Annotated With Risk of Bias Text Spans in Randomized Controlled Trials in Physiotherapy and Rehabilitation: Corpus Development and Annotation Study

**DOI:** 10.2196/55127

**Published:** 2026-04-27

**Authors:** Anjani Dhrangadhariya, Roger Hilfiker, Karl Martin Sattelmayer, Nona Naderi, Katia Giacomino, Rahel Caliesch, Julian Higgins, Stéphane Marchand-Maillet, Henning Müller

**Affiliations:** 1 Informatics Institute, HES-SO Valais-Wallis, Rue du Technopole 3, Sierre, 3960, Switzerland, 41 787084007; 2 Institute of Health Sciences, HES-SO Valais-Wallis, Thermenstrasse 41, Leukerbad, Valais, Switzerland; 3 Physiotherapy Tschopp & Hilfiker, Brig, Valais, Switzerland; 4 Centre national de la recherche scientifique, Laboratoire Interdisciplinaire des Sciences du Numérique, Université Paris-Saclay, Orsay, France; 5 Population Health Sciences, Bristol Medical School, University of Bristol, Bristol, United Kingdom; 6 Centre d'Informatique Universitaire, University of Geneva, Geneva, Switzerland; 7 Medical Faculty, University of Geneva, Geneva, Switzerland; 8 The Sense Innovation and Research Center, Sion, Switzerland

**Keywords:** risk of bias, corpus annotation, natural language processing, large language models, LLM, information extraction, RoBuster, corpus, randomized controlled trials, RCT, reviewer, tools, physiotherapy, rehabilitation, effectiveness

## Abstract

**Background:**

Risk of bias (RoB) assessment of randomized clinical trials (RCTs) is vital to answering systematic review questions accurately. Manual RoB assessment for hundreds of RCTs is a cognitively demanding and lengthy process. Automation has the potential to assist reviewers in rapidly identifying text descriptions in RCTs that indicate potential risks of bias. However, no RoB text span annotated corpus could be used to fine-tune or evaluate large language models (LLMs), and there are no established guidelines for annotating the RoB spans in RCTs.

**Objective:**

The revised Cochrane RoB 2 test (RoB 2) tool provides comprehensive guidelines for RoB assessment; however, due to the inherent subjectivity of this tool, it cannot be directly used as RoB annotation guidelines. The study aimed to develop precise RoB text span annotation instructions that could address this subjectivity and thus aid the corpus annotation.

**Methods:**

We leveraged RoB 2 guidelines to develop visual instructional placards that serve as annotation guidelines for RoB spans and risk judgments. Expert annotators used these visual placards to annotate a dataset named RoBuster, consisting of 41 full-text RCTs from the domains of physiotherapy and rehabilitation. We report interannotator agreement (IAA) between 2 annotators for text span annotations before and after applying visual instructions on a subset (n=9) of RoBuster. We also provide IAA on bias risk judgments using Cohen κ. Moreover, we used a portion of RoBuster (n=10) to evaluate an LLM using a straightforward evaluation framework. This evaluation aimed to gauge the performance of an LLM (here GPT 3.5) in the challenging task of RoB span extraction and demonstrate the utility of this corpus using a straightforward framework.

**Results:**

We present a corpus of 41 RCTs with fine-grained text span annotations comprising more than 28,427 tokens belonging to 22 RoB classes. The IAA at the text span level calculated using the F1 measure varies from 0% to 90%, while Cohen κ for risk judgments ranges between –0.235 and 1.0. Using visual instructions for annotation increases the IAA by more than 17 percentage points. LLM (GPT-3.5) shows promising but varied observed agreements with the expert annotation across the different bias questions.

**Conclusions:**

Despite having comprehensive bias assessment guidelines and visual instructional placards, RoB annotation remains a complex task. Using visual placards for bias assessment and annotation enhances IAA compared to cases where visual placards are absent; however, text annotation remains challenging for the subjective questions and the questions for which annotation data are unavailable in RCTs. Similarly, while GPT-3.5 demonstrates effectiveness, its accuracy diminishes with more subjective RoB questions and low information availability.

## Introduction

Systematic reviews (SRs) synthesized using randomized controlled trials (RCTs) are the highest quality of evidence in the evidence pyramid. SRs help medical professionals make informed health decisions and guide health policies [1,2]. An RCT tests an intervention’s effectiveness by randomly assigning patients to intervention groups; for example, the impact of the intervention under investigation is compared to other interventions in a controlled setting [3]. Theoretically, RCTs are low on biases given the randomized design, but biases can still infiltrate the design, execution, or reporting phases. Such biases may cause medical professionals to misjudge intervention effects, impacting health outcomes [4,5]. Therefore, bias assessment, known as risk-of-bias (RoB) assessment, is vital for RCTs used for writing SRs.

There are several tools to assess RoB, including the Cochrane Collaboration’s RoB Tool, PEDro RoB scale, revised Cochrane RoB 2 tool, AMSTAR (A Measurement Tool to Assess Systematic Reviews) or AMSTAR 2, EPOC (Effective Practice and Organization of Care) RoB Tool, and other checklists [6-11]. These tools provide structured questions to elicit bias-relevant information from RCTs. Manual RoB assessment, a time-consuming task requiring substantial expertise, can take hours per RCT and months for a full SR, emphasizing the need for automation [12,13]. Moreover, writing SRs is highly resource-heavy, taking about 6 months to several years to complete [6,14,15]. Machine learning (ML) could expedite this process by pinpointing bias-relevant RCT text, aiding quicker quality assessments [16]. Marshall et al [17] attempted automation of RoB assessment using a distant supervision approach supported by proprietary data from CDSR. The study was supported by the manually entered data from CDSR, which is behind a paywall and automates based on Cochrane RoB 1 guidelines and not the latest RoB 2 [7]. Although RoB 1 is most frequently used for assessment, the recently revised Cochrane RoB 2 offers significant differences [18]. Compared to RoB 1, RoB 2 provides a reliable and concrete structure to the RoB evaluation by developing comprehensive guidelines that aim to increase consistency [7,11]. The use of RoB 2 increased from 0% in 2019 to 24.1% in 2022, indicating the need to switch to this updated tool for bias assessment [19].

Millard et al [20] also explored ML-based RoB assessment using proprietary data. Their work using these pay-walled data was used to develop RobotReviewer, which has been evaluated by several studies for its human-level performance [17,21-24]. A lack of public RoB-annotated data still limits community advancements. Wang et al [25] recently released RoB datasets for animal studies, but human clinical trials still lack a comprehensive RoB corpus. Manual RoB assessment is a complex, expert-led task laden with subjective judgments [26,27]. Systematically translating this manual process for developing a RoB annotated corpus requires a carefully designed annotation scheme and annotation guidelines. We previously worked on a pilot study to test whether RoB 2 guidelines could be effectively used as annotation guidelines to annotate a corpus of RCTs with RoB. We concluded that RoB 2 cannot be used as text annotation guidelines but did not provide any annotation guidelines [28]. Here, we aim to establish clear annotation guidelines to annotate RCTs with RoB spans corresponding to the RoB 2 tool [11].

In addition, recent large language models (LLMs) have shown potential in handling complex tasks with minimal instructions [29]. However, their capability to identify RoB spans in RCTs has yet to be assessed. Our contributions with this paper are 5-fold as follows:

1. Development of detailed annotation guidelines for RCT RoB spans.
2. Development of visual placards to simplify annotation and assist trainee RoB assessors.
3. Compilation of “RoBuster,” a corpus of 41 annotated RCTs with 22 RoB span types, for ML and LLM training or benchmarking.
4. Evaluation of an LLM in identifying answers to signaling questions using prompts.
5. Open sharing of annotation guidelines, dataset, and LLM prompts with the community.

## Methods

### Overview

**Figure 1.**
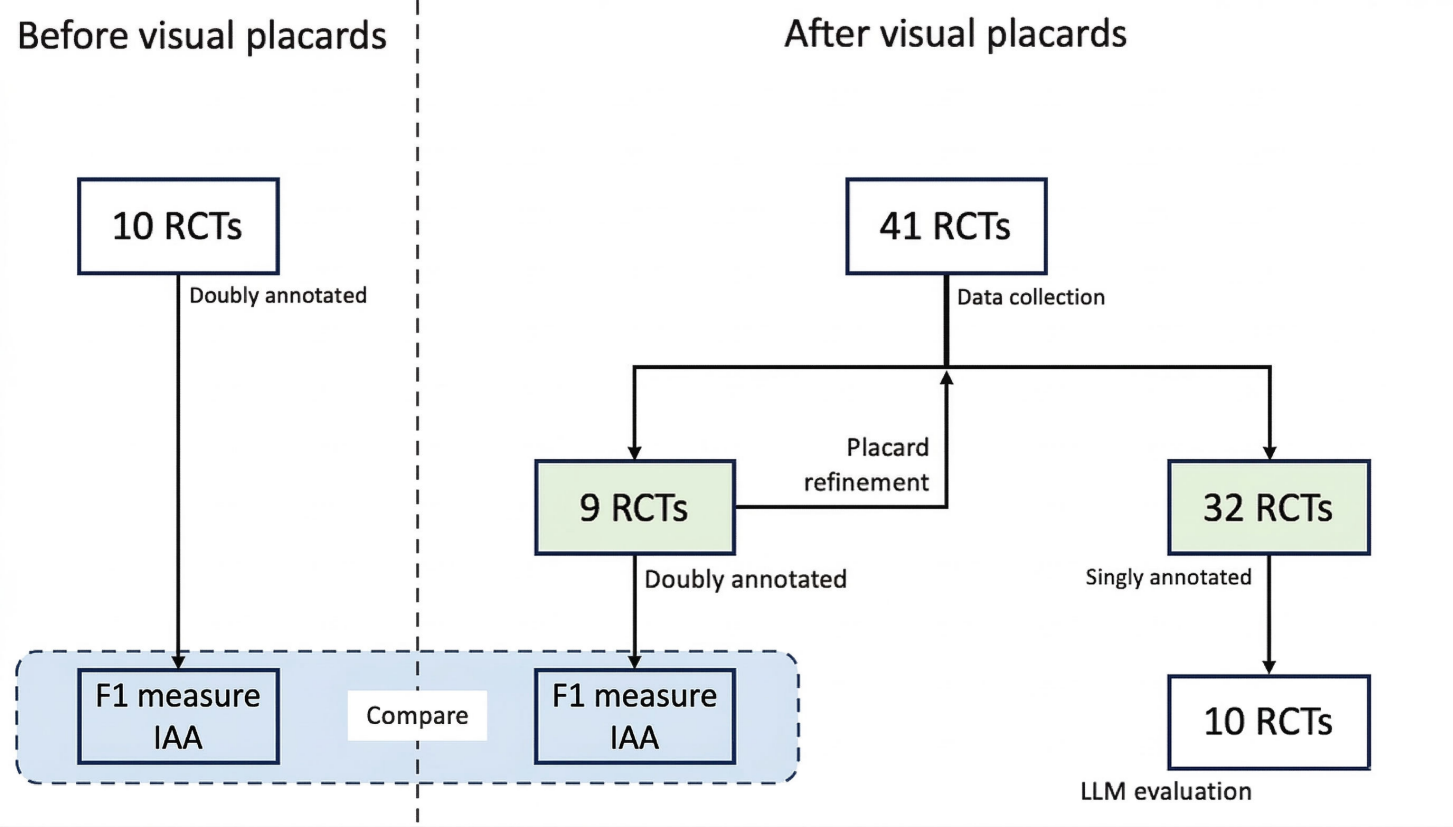
The flowchart (right) illustrates our methodology starting from data collection until interannotator agreement (IAA) calculation. The agreement is calculated between 10 RCTs annotated (left) in Dhrangadhariya et al [28] and 9 RCTs from the current work. LLM: large language model; RCT: randomized controlled trial

This section details the annotation scheme, software, and visual placard development. With no existing RoB span annotation guidelines, we created them from scratch based on the revised Cochrane RoB 2 tool [6]. We created a draft of the visual guidelines, doubly annotating a fraction of RCTs using it, and refined the guidelines using identified conflicts. Figure 1 illustrates our methodology starting from data collection.

### Expert Team

RoB annotation requires specialized expertise due to the need to thoroughly review the entire RCT text to identify 22 different bias categories. Our annotation team comprised 2 experts in RoB assessment in the physiotherapy and rehabilitation domains: an epidemiology researcher (RH) and an associate professor in physiotherapy (KMS), both with extensive experience in physiotherapy, statistical methods, and SRs. Two senior PhD students (KG and RC) contributed to the development of the visual annotation guidelines. Two researchers in natural language processing, an associate professor in computational linguistics (NN) and a PhD student in computer science (AKD), assisted in creating the visual guidelines, which serve as a benchmark for RoB text span extraction. Finally, JPTH provided feedback to shape the visual annotation guidelines (Multimedia Appendix 1).

### Ethical Considerations

In accordance with the Swiss Federal Act on Research involving Human Beings (Human Research Act) of September 30, 2011 (SR 810.30), formal ethical approval by a Cantonal Ethics Committee was not required as the research did not concern human diseases or the structure and function of the human body. The experts who undertook the visual placards development and the annotation process for this corpus were informed about the purpose of the annotation project and agreed to voluntarily participate in the study. Even though they agreed to participate in the study, they can withdraw their participation at any time without consequences of any kind. They were informed of the purpose and nature of the study via a presentation and had an opportunity to ask questions. They were also fully informed about the eventual publication of the findings. Each expert provided consent for the publication of the annotated corpus, along with the understanding that any identifying information would be appropriately anonymized to protect their privacy. The expert annotators volunteered their time and received no financial compensation.

### Annotation Scheme

Creating a new annotated corpus requires defining or adopting an annotation scheme. To our knowledge, the only existing RoB span annotation scheme is from our previous work [28]. Rather than developing a new one, we adapted and enhanced this scheme, addressing prior limitations. The scheme aligns with the RoB 2 assessment, which organizes bias into 5 domains reflecting different trial design aspects. Each risk domain decomposes into several signaling questions totaling 22 (Table 1). Each question prompts the assessor to look for relevant text evidence in the trial and judge risk response for that signaling question (SQ; Table 2). For detailed explanations of the SQs, see Cochrane RoB 2.0 guidelines as the original document and Multimedia Appendix 1 [6].

**Table 1.** The table lists signaling questions from each bias domain from the revised Cochrane Risk of Bias (RoB) tool.

Question number	Signaling question
RoB 1	
RoB 1.1	Was the allocation sequence random?
RoB 1.2	Was the allocation sequence concealed until participants were enrolled and assigned to interventions?
RoB 1.3	Did baseline differences between intervention groups suggest a problem with the randomization process?
RoB 2	
RoB 2.1	Were participants aware of their assigned intervention during the trial?
RoB 2.2	Were carers and people delivering the interventions aware of participants’ assigned intervention during the trial?
RoB 2.3	Were there deviations from the intended intervention that arose because of the trial context?
RoB 2.4	Were these deviations likely to have affected the outcome?
RoB 2.5	Were these deviations from the intended intervention balanced between groups?
RoB 2.6	Was an appropriate analysis used to estimate the effect of assignment to intervention?
RoB 2.7	Was there potential for a substantial impact (on the result) of the failure to analyze participants in the group to which they were randomized?
RoB 3	
RoB 3.1	Were data for this outcome available for all, or nearly all, participants randomized?
RoB 3.2	Is there evidence that the result was not biased by missing outcome data?
RoB 3.3	Could missingness in the outcome depend on its true value?
RoB 3.4	Is it likely that missingness in the outcome depended on its true value?
RoB 4	
RoB 4.1	Was the method of measurement of the outcome inappropriate?
RoB 4.2	Could measurement or ascertainment of the outcome have differed between intervention groups?
RoB 4.3	Were outcome assessors aware of the intervention received by study participants?
RoB 4.4	Could the assessment of the outcome have been influenced by knowledge of the intervention received?
RoB 4.5	Is it likely that the assessment of the outcome was influenced by knowledge of the intervention received?
RoB 5	
RoB 5.1	Were the data that produced this result analyzed in accordance with a pre-specified analysis plan that was finalized before unblinded outcome data were available for analysis?
RoB 5.2	Is the numerical result being assessed likely to have been selected, on the basis of the results, from multiple eligible outcome measurements (eg, scales, definitions, time points) within the outcome domain?
RoB 5.3	Is the numerical result being assessed likely to have been selected, on the basis of the results, from multiple eligible analyses of the data?

**Table 2.** The table lists bias domains from the revised Cochrane Risk of Bias (RoB) tool and the number of signaling questions (SQs) per domain.

Class	Domain	SQ
RoB 1	Biases arising from the *randomization process*	3
RoB 2	Biases due to *deviations from intended interventions*	7
RoB 3	Bias due to *missing outcome data*	4
RoB 4	Bias in the *measurement of the outcome*	5
RoB 5	Bias in the *selection of the reported result*	3

The response options for the RoB judgment include “Yes,” “Probably yes,” “No,” “Probably no,” or “No information.” Reviewers assess each SQ by examining the factual evidence in the RCT. For example, the SQ “Was the allocation sequence random?” is assessed by checking the randomization method. A well-executed method results in a “Yes” response (low risk), while a poorly executed method leads to a “No” (high risk) [11].

**Figure 2.**
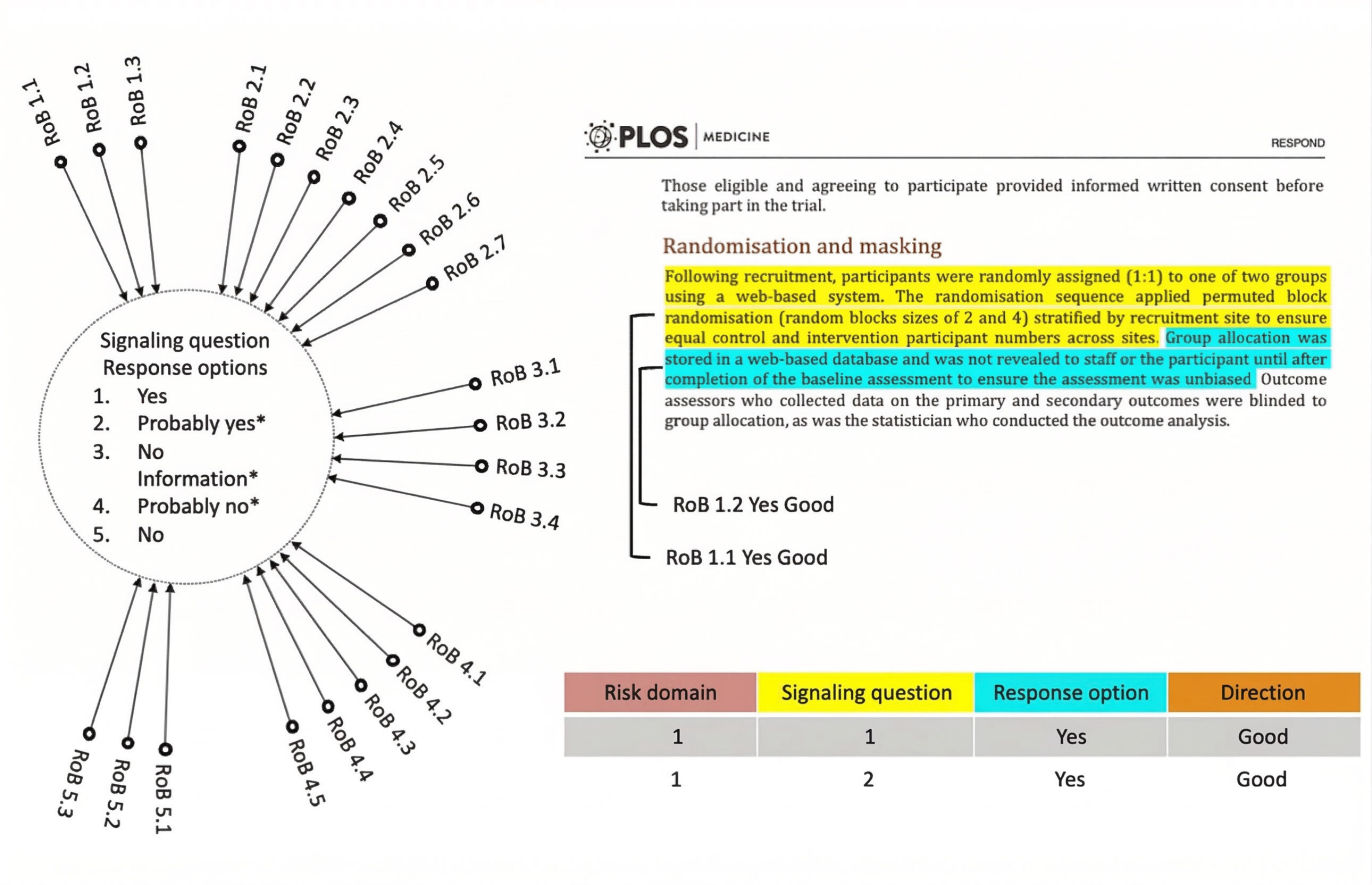
Algorithm for suggested judgment of risk of bias (RoB) arising from the randomization process. The figure is recreated from the revised Cochrane RoB 2 tool (RoB 2) [6].

**Figure 3.**
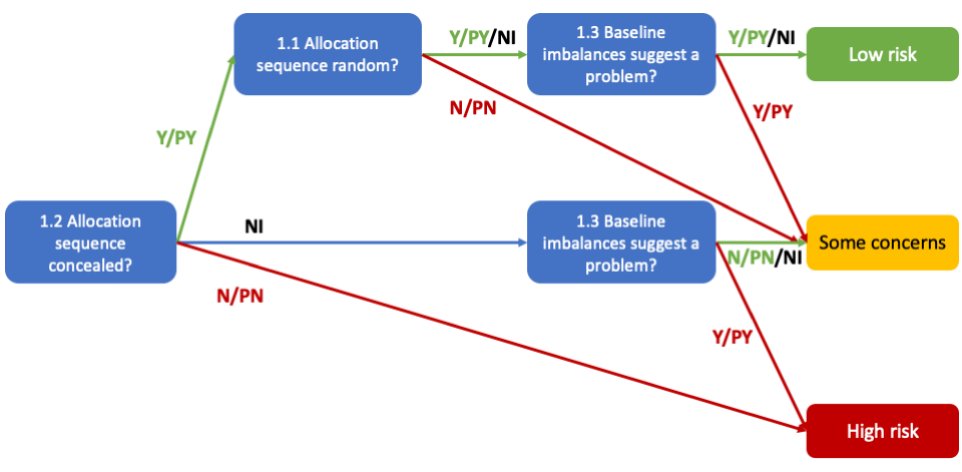
Algorithm for suggested judgment of risk of bias arising from the randomization process. The figure is recreated from the revised Cochrane Risk of Bias 2 tool. N: no; NI: no information; PN: probably no; PY: probably yes; Y: yes [6].

For RoB span annotation, we mimic the assessment process by considering evidence text spans in the RCT as the main units of annotation. Each span corresponds to an answer for a specific SQ and is annotated with a label. Multiple spans (sentence and paragraph) across the RCT can be annotated to answer a single SQ if needed. The annotation label incorporates information about the SQ number and its domain (for the above example, “1.1” for the first domain and first SQ of the domain). The response option for risk judgment is incorporated in the label, such as “1.1 Yes Good” for a well-executed randomization procedure and “1.1 No Bad” otherwise (Figure 2). To improve interannotator agreement (IAA), we collapse “Yes” and “Probably Yes” to a single “Yes,” and similarly for “No” and “Probably No” [28]. As shown in Figure 3, these collapsed responses do not affect the final risk judgment for a domain (low, high, or some concerns). Therefore, except for some special case SQs, we collapse these response options in this work. Our scheme includes 22 entities for the 22 SQs, each with 2 response options (“Yes” or “No”) and 2 risk judgments (“Good” or “Bad”); “Good” implies low risk and “Bad” implies high risk. The “No Information” option is removed unless there’s truly no text evidence, though it remains for specific SQs like SQ 2.1. For instance, if a trial describes a “random number generator and sealed envelopes” but lacks details on envelope opacity, “No Information” is considered an appropriate label.

### Data Collection

A dataset of 41 RCTs from the physiotherapy and rehabilitation domains was compiled by RH. The RCTs included in the corpus were carefully curated from a selection of high-quality journals in physiotherapy and rehabilitation, as recommended by our institute’s librarian (eg, PLoS). To ensure consistency with modern reporting practices, we included only RCTs published after 2010. To facilitate open sharing and publication of the annotated corpus, we included only articles available under the CC-BY-0 license. Additional details are provided in Multimedia Appendix 1.

To create this corpus, PDFs of full-text RCTs were extracted, and each article was paired with its trial registry whenever available. Each PDF was renamed with the primary outcome to be assessed using the RoB 2 tool before being uploaded to the annotation software. To ensure that various primary outcome types were represented in the corpus, we included 17, 17, and 7 RCTs addressing objective, subjective, and mortality primary outcomes, respectively, following the rationale that RoB assessment results are related to the outcomes [30-33]. The rationale behind this is described in Multimedia Appendix 1.

### Visual Placards Development

Although RoB 2 guidelines are widely used for bias assessment, there has been some research on their reliability. Minozzi et al [26,34] addressed this issue by creating an ID that reduces subjectivity in the RoB 2 guidelines, providing clearer instructions for assessment. Before implementing the ID, the agreement among 4 expert RoB assessors in the Minozzi et al’s [26,34] study was zero, but it improved after adopting the ID. Several other papers explored the subjectivity and reliability of Cochrane RoB 1 and 2 tools [35,36]. To enhance consistency and reliability, we developed precise annotation instructions using the RoB 2 tool and collaborated with experts to format these into visual placards. Each placard, structured as a flowchart, guides annotators in answering SQs and labeling text with risk judgments. While RoB 2 SQs are mainly factual, they allow for subjective judgments, which the placards help standardize.

### Annotation

The annotation process for the 41 RCTs (see “Data Collection” section) began after developing visual placards. Annotators used the complete RoB 2 guidance alongside these placards, following instructions closely for each SQ. For each SQ, the placards guided annotators to relevant sections within the RCTs, to identify and highlight pertinent text to answer each question, selecting labels as defined in the annotation scheme. Domain 2 of RoB 2 was assessed with respect to the effect of assignment to the intervention (intention-to-treat estimand). Signaling questions related to the “effect of adhering to the intervention” were not annotated.

**Figure 4.**
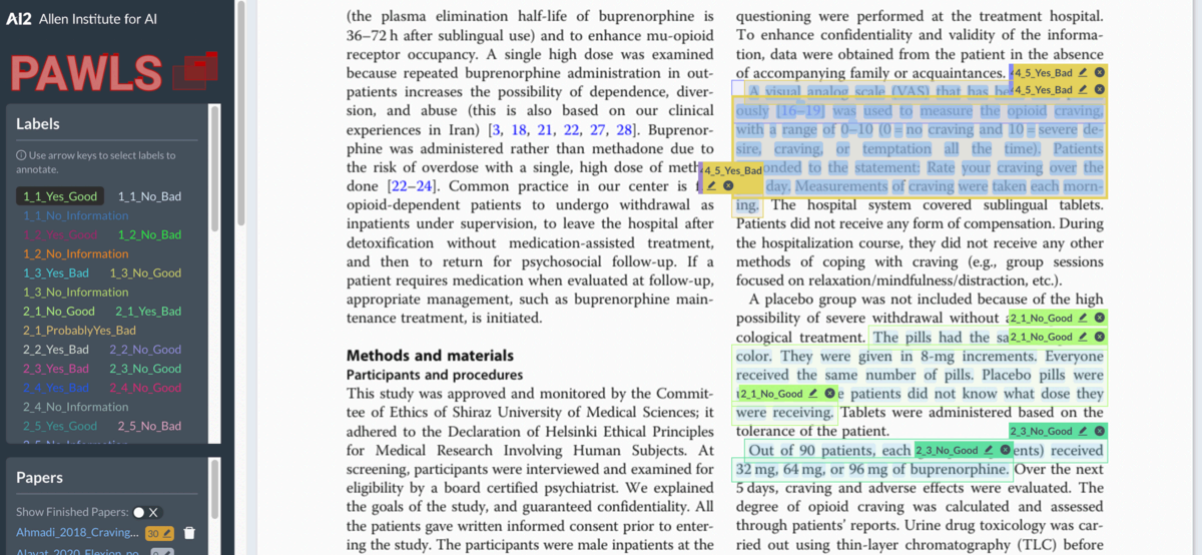
A screenshot of PAWLS (PDF Annotation With Labels and Structure; Allen Institute for Artificial Intelligence) interface with an example PDF and risk of bias (RoB) annotations.

The annotation was done in Tagtog (tagtog Sp. z o.o.), a commercial PDF annotation tool [37]. Of the 41 RCTs, 9 were doubly annotated by RH and KMS to calculate IAA, with the remaining (n=32) singly annotated by RH. Conflict resolution on the doubly annotated RCTs helped refine the visual placards before annotating the rest. After annotating the 9 RCTs, we transitioned to the PAWLS (PDF Annotation With Labels and Structure) annotation tool (Allen Institute for Artificial Intelligence; Figure 4), a free PDF annotation platform [38]. Annotating PDFs preserves the structure of sections, tables, and figures, improving annotation speed and quality and ease of annotation for our experts who volunteered for annotation. Feedback from them is detailed in Multimedia Appendix 2.

### IAA Measure

We report IAA on the doubly annotated RCTs. The IAA was calculated at 2 levels, assessing annotator agreement on text spans for SQs using pairwise F1, which excludes unannotated tokens and is well-suited for token-level annotation tasks. Pairwise F1 is measured as shown below for each pair of annotators by treating one annotator’s labels as “true” and the other’s as “predicted” [39,40]. In this study, IAA was measured after incorporating visual placards into the annotation process. To evaluate the impact of these placards on annotation quality, we also compare the F1 IAA with results from our previous work, where n=10 RCTs were annotated without the use of placards (Figure 1).


$$F1-measure=\frac{2\times True\;Positive}{2\times True\;Positive+False\;Positive+False\;Negative}$$

We also check annotator agreement on risk judgments for each SQ using prevalence and bias-adjusted κ (PABAK) and observed percent agreement. PABAK (κ_PABAK_), an extension of Cohen κ that accounts for prevalence and bias, is commonly used for classification tasks and is ideal for evaluating reliability at the risk judgment level. Interpretation guidelines for both IAA measures are shown in Table 3 [41-44].

**Table 3.** Interpretation of pairwise F1-measure, κ_PABAK_, and observed agreement.

Metric	Value
Pairwise F1	
Poor	0‐0.99
Slight	1‐20.99
Fair	21‐40.99
Good	41‐60.99
Substantial	61‐80.99
Almost perfect	81‐99.99
Perfect	100
κ_PABAK_	
No agreement	≤0
None to slight	0.0-0.20
Minimal	0.21‐0.39
Weak	0.40‐0.59
Moderate	0.60‐0.79
Strong	0.80‐0.90
Almost perfect	≥0.90
Perfect	100
Observed agreement	
None	0%
Very low	1%‐10%
Low	11%‐30%
Moderate	31%‐50%
High	51%‐70%
Very high	71%‐90%
Perfect	>90%

### LLM Evaluation

Our annotation guidelines were initially adapted for benchmarking traditional ML approaches rather than LLMs. This meant we restricted certain annotations, assuming the PDFs would be converted into text via optical character recognition, thus losing table and figure structures that classical ML models cannot interpret without significant adjustments [45,46]. Recent advancements with LLMs have offered a better alternative and have made us rethink the evaluation. The bar for clinical applications is high, and it is necessary to evaluate LLMs for the challenging clinical tasks like RoB span extraction [47]. ChatPDF allows direct interaction between LLMs and PDFs, negating the clumsy PDF-to-text conversion [48]. Therefore, we consider it essential to evaluate LLMs instead of forcefully adapting the evaluation to a classical ML problem.

We formulated the task as a zero-shot RoB text span extraction task with an aim to gauge whether an LLM encodes knowledge related to assessing trial biases. We used simple prompt constructs of the structure “Answer the {*SQ*} + Action item to extract sentence supporting the answer” (Textbox 1). ChatPDF used these prompts to identify relevant paragraphs and generate answers to the SQs using GPT-3.5, mirroring the annotators’ task.

Textbox 1. **Example prompt used for large language model evaluation.**

Question 4.3: Were outcome assessors aware of the intervention received by study participants? Provide an answer and extract the supporting sentences that you write your answer based on. Extract the sentences in JSON format.

LLM performance was measured by agreement on response options and extracted text span or evidence. If the LLM’s response matched the expert annotator’s selection, it was considered correct. If the text extracted by LLMs as evidence for answering the SQ fuzzy matches the text selected by the expert annotator, it is considered a correct answer. Both skills were evaluated using observed agreement metrics, $P_{O}\left(Extraction\right)$ for measuring agreement over extraction and $P_{O}\left(Response\right)$for measuring agreement over response judgments and interpreted as per Table 3. Observed agreement is essentially the number of documents for an RoB SQ where LLM responses align with those of the human expert, divided by the total number of documents assessed [49]. For cases where annotators found no information in RCT, ChatPDF’s ability to recognize this absence was also evaluated. We set the temperature to 0 to ensure a deterministic setting for span extraction and response generation. This ensures exact text spans are extracted from the input RCT. This evaluation was done manually for 10 out of the 41 annotated RCTs. Details of the RCTs used for LLM evaluation are in Multimedia Appendix 1.

## Results

### Visual Placards

**Figure 5.**
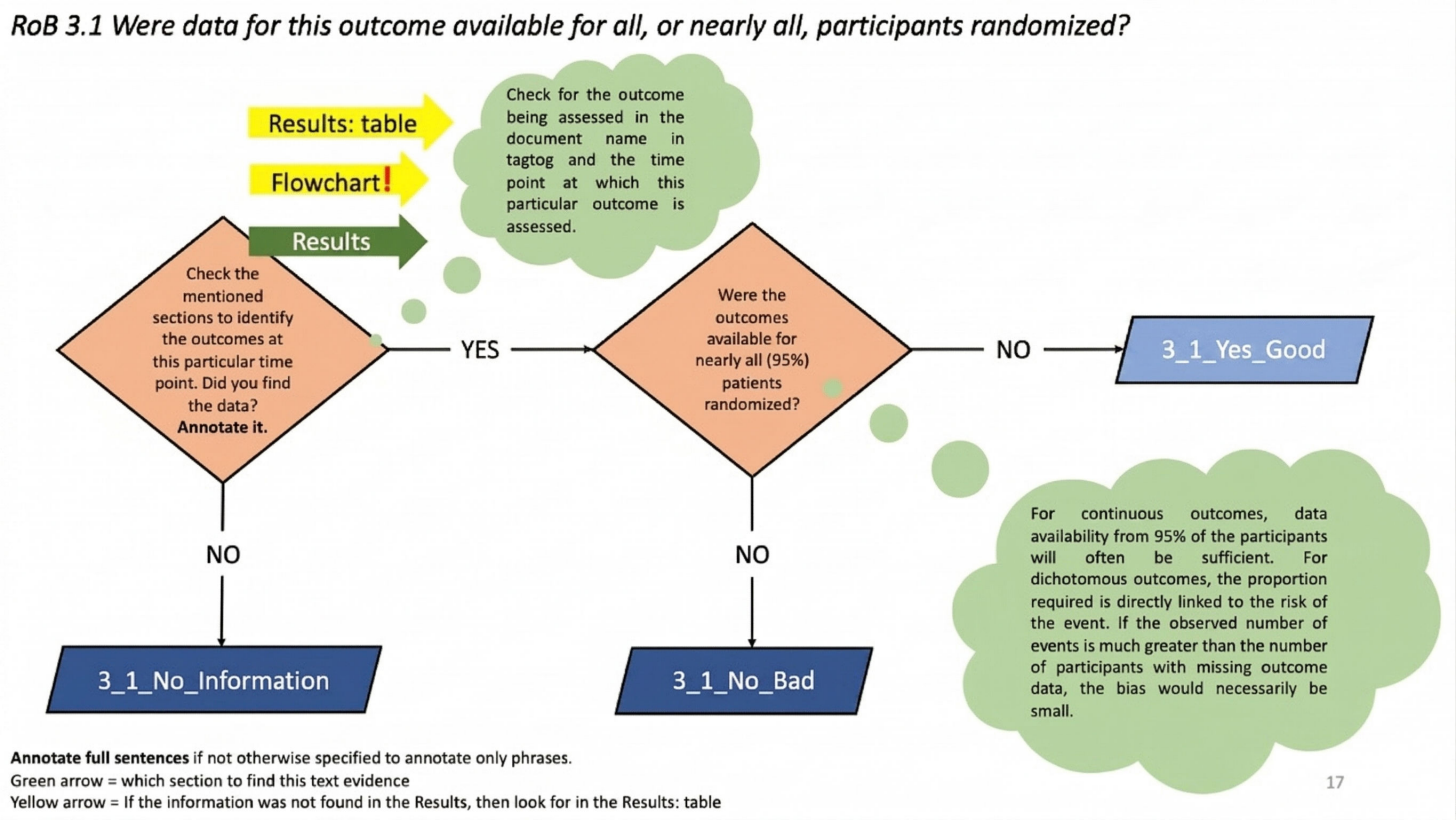
Sample annotation instruction placard for the signaling question (SQ) 3.1 designed and adapted using the Cochrane Risk of Bias (RoB) 2 tool [6].

A total of 27 placards were developed to address the 22 SQs. Details of the annotation guidelines and visual placards are available in MultimediaAppendices 1  3. Figure 5 shows an example placard for annotating SQ 3.1 (“Were data for this outcome available for all, or nearly all participants randomized?”), which assesses the completeness of outcome data in an RCT. Missing outcomes data can compromise statistical power and treatment effect estimates. The first diamond on the placard instructs annotators to check the “Results” section (priority indicated by green arrow) and the flowchart and table within the “Results” section (second priority) to identify outcome data at the specified time point. If outcomes data were available for at least 95**%** of participants, annotators mark relevant text descriptions as “3.1 Yes Good,” indicating a low bias. If data were available for fewer than 95**%**, they mark it as “3.1 No Bad,” indicating a high bias. The placard includes visual cues to guide the annotation process efficiently.

### The Corpus: RoBuster

**Figure 6.**
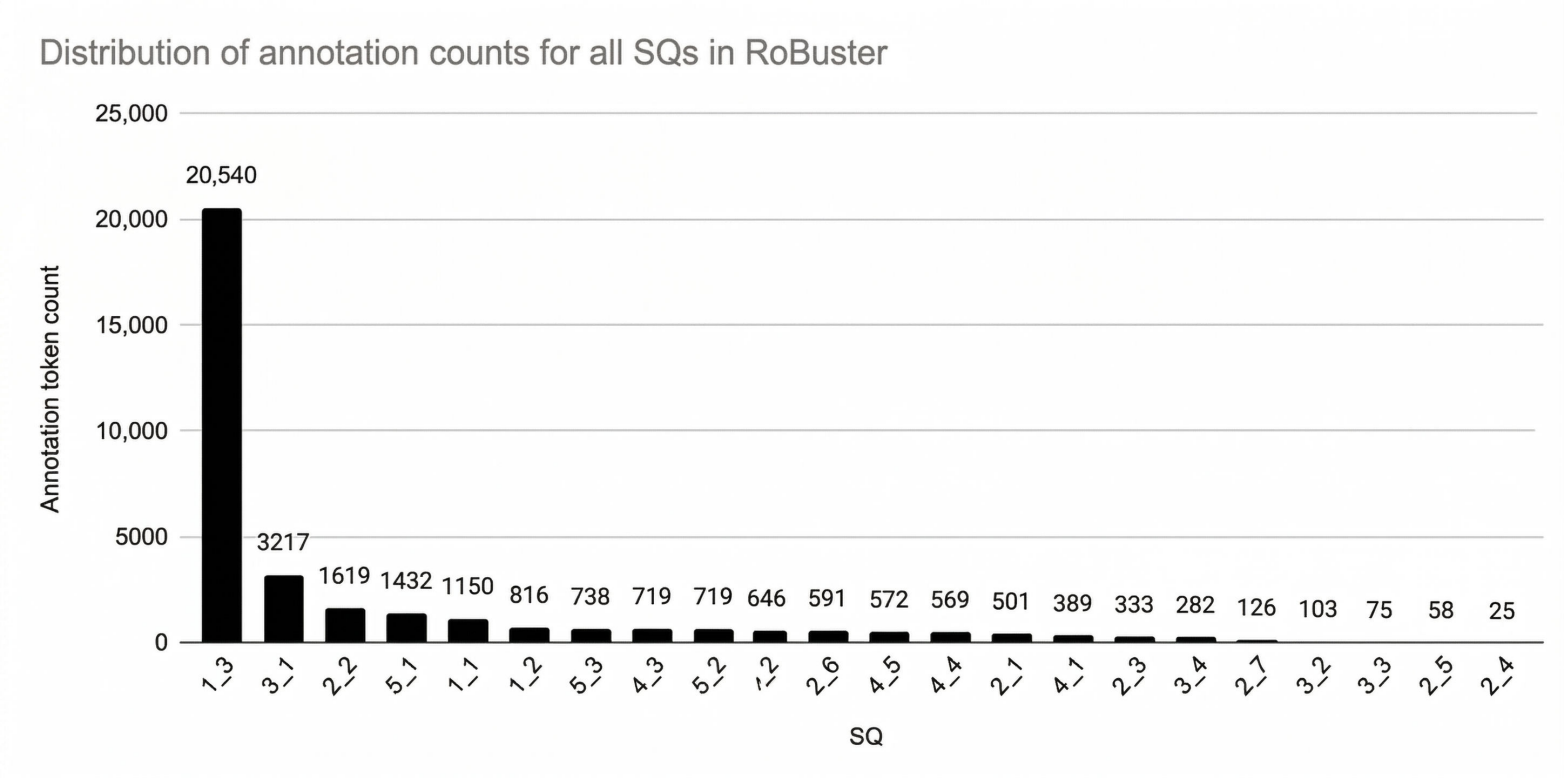
Total number of token annotations in RoBuster for each risk of bias (RoB) signaling question (SQ).

Figure 6 shows that SQ 1.3 had a much higher number of annotated tokens (n=16,446), compared to fewer than 2600 tokens for other SQs. This is because SQ 1.3 required annotating the entire baseline patient characteristics table, as instructed by the visual placards. For other questions, the number of annotated tokens depended on the amount of detail provided on study design, methods, and results, which affected both annotation count and assessment subjectivity. Most other SQs had fewer than 2000 annotated tokens, with SQ 3.1 slightly exceeding this threshold. SQ 2.4 had the fewest tokens, with only 25 tokens.

**Figure 7.**
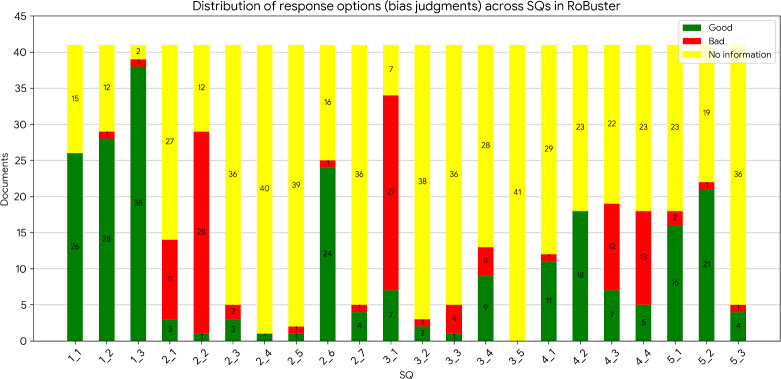
Distribution of bias judgment across risk of bias (RoB) signaling questions (SQs) in RoBuster.

Figure 7, which shows the distribution of risk judgments across RoBuster, highlights that, for most SQs, no information was available (yellow bars) for answering the SQs. Exceptions included SQs 1.1, 1.2, 1.3, 2.2, 2.6, 3.1, 4.3, 4.4, and 5.2, where over 50% of documents had relevant information. In cases where even some information was available, bias tended to be low (green bar) with an exception for the SQs 2.1, 2.2, 3.1, 4.3, and 4.4, where bias was high (red bar). Studies with comprehensive information made evaluation easier, whereas those lacking key details made it challenging. Annotator feedback indicated that questions with fewer than 100 annotated tokens were consistently rated as “(very) low” information availability, whereas the top 5 SQs shown in Figure 6 were rated as “high” or “normal” availability. All study references are listed in Multimedia Appendix 1.

### IAA Measure

Table 4 presents the F1-measure (IAA) between 2 annotators, both before and after the development of the visual placards. The F1-measures before and after the guideline improvement were calculated on a different set of documents. To reiterate, the F1 IAA results before the visual placard development were drawn from our previous work, where 10 RCTs were annotated in the absence of the placards [28]. Initially, the average agreement across the corpus was 10.87%, which rose by 17.14% points to reach 28.01% with placard use. For the “randomization” domain, agreement improved from a low 31.72% to 63.30%. In the “deviations from intended interventions” domain, it increased from 12.76% to 27.02%. Agreement in the “missing outcomes” domain rose from 5.89% to 9.92%, while “missing outcome measurement” increased from 4.07% to 17.29%. For “selection of reported results,” agreement increased from 0% to 16.49%.

**Table 4.** The table displays the F1-measure at the text span annotation level before and after visual placard development, with changes in absolute interannotator agreement (IAA) points.

Domain and SQ^a^		F1-measure IAA		Change
		Before guideline improvement	After guidelines improvement	
Domain 1				
RoB^b^ 1.1		24.44	55.02	+30.58
RoB 1.2		50.28	44	–6.28
RoB 1.3		20.44	90.9	+70.46
Domain 2				
RoB 2.1		1.34	67.26	+65.92
RoB 2.2		7.23	38.66	+31.43
RoB 2.3		5.42	0	–5.42
RoB 2.4		—^c^	0	0
RoB 2.5		0	0	0
RoB 2.6		68.85	83.25	+14.4
RoB 2.7		6.52	0	–6.52
Domain 3				
RoB 3.1		23.57	39.68	+16.11
RoB 3.2		0	0	0
RoB 3.3		0	0	0
RoB 3.4		0	0	0
Domain 4				
RoB 4.1		6.51	61.71	+55.2
RoB 4.2		0	0	0
RoB 4.3		13.85	30.21	+16.36
RoB 4.4		0	56.25	+56.25
RoB 4.5		0	0	0
Domain 5				
RoB 5.1		0	0	0
RoB 5.2		0	49.49	+49.49
RoB 5.3		0	0	0

a SQ: signaling question.

b RoB: risk of bias.

c Dash (—) indicates that one of the annotators did not annotate any text for a particular SQ.

Substantial gains of over 50% points were seen in SQs 1.3, 2.1, 4.1, and 4.4. However, for 11 out of 22 questions, agreement remained at zero, and for nine of these, the lack of agreement persisted postguideline update. In contrast, agreement improved for SQs 4.4 and 5.2 from 0% to 56.25% and 49.49%, respectively, while SQs 2.3, 2.7, and 1.2 saw slight agreement declines. Across the 22 SQs, 11 had poor agreement, 3 fair, 4 good, 2 substantial, and 2 near-perfect (>81%); none of the SQs achieved a perfect F1-measure.

Table 5 presents the κ_PABAK_ agreement, observed agreement, and the percentage of κ_PABAK_ agreements stemming from “No Information” judgments. κ_PABAK_ measures agreement at the SQ risk judgment level. Overall κ_PABAK_ between annotators shows weak IAA at 0.412. Agreement for “randomization” (domain 1) averaged a moderate 0.629, “deviations due to intended interventions” (domain 2) was 0.64, and “missing outcome data” (domain 3) was minimal at 0.388. Domains 4 (“outcome measurement”) and 5 (“selection of reported result”) had slight or no agreement at 0.166 and 0.092, respectively.

**Table 5.** κ_PABAK_ and observed agreement between annotator pairs at the risk judgment level for each signaling question (SQ).

Domain and SQ		κ_PABAK_ agreement	Observed agreement (%)	Contribution from “No information” (%)
Domain 1				
RoB^a^ 1.1		0.8333	88.90	22.22
RoB 1.2		0.5	66.70	33.33
RoB 1.3		0.5556	77.80	11.11
Domain 2				
RoB 2.1		0.7037	77.80	5.56
RoB 2.2		0.5	66.70	38.89
RoB 2.3		0.5	66.70	77.78
RoB 2.4		0.5556	77.80	88.89
RoB 2.5		0.6667	77.80	83.33
RoB 2.6		1	100	55.56
RoB 2.7		0.5556	77.80	77.78
Domain 3				
RoB 3.1		0.8333	88.90	11.11
RoB 3.2		—^b^	100	100
RoB 3.3		0	33.3	55.56
RoB 3.4		0.3333	55.60	33.33
Domain 4				
RoB 4.1		0.5556	77.80	11.11
RoB 4.2		–0.5556	22.20	38.89
RoB 4.3		0.6667	77.80	27.78
RoB 4.4		0.1667	44.40	22.22
RoB 4.5		0	33.30	66.67
Domain 5				
RoB 5.1		–0.5556	22.20	61.11
RoB 5.2		0.5	66.70	22.22
RoB 5.3		0.3333	55.60	72.22

a RoB: risk of bias.

b Dash (—) indicates that one of the annotators did not annotate any text for a particular SQ.

The highest agreement, 1.0, occurred for SQ 2.6, largely due to “No Information” judgments. No SQs showed almost perfect or strong agreement; moderate agreements (0.60‐0.80) were observed for SQs 1.1, 2.1, 2.5, 3.1, and 4.3, with “No Information” judgments impacting only SQs 1.1, 2.1, 3.1, and 4.3. Two SQs (4.2 and 5.1) had agreements worse than chance, with one annotator marking no text in SQ 5.1, resulting in negative IAA, and both annotators marking different parts of the text in SQ 4.2. SQs 3.3 and 4.5 had zero κ_PABAK_ due to mutually exclusive document annotations, causing no consensus.

### LLM Evaluation

Table 6 shows the observed agreement between the LLM and expert assessments for extracting and responding to SQs on a subset (n=10) of RoBuster. In domain 1, GPT-3.5 had high agreement with experts (66.6% for extraction and 55.3% for response judgment), with no reliance on “No Information” responses, indicating good information availability. For domain 2, observed agreements were 64.3% (extraction) and 60.0% (response), but 40% of these were “No Information” responses, showing lower reporting quality. Domain 3 had moderate agreement at 47.5% for both extraction and response, while domain 4 had lower agreement (28% for response and 26% for extraction). To enhance transparency, we have included both the LLM-generated responses (via ChatPDF) and the corresponding annotator responses as Multimedia Appendix 4.

**Table 6.** Large language models (LLMs) evaluation—observed agreements between LLM and experts over a subset of RoBuster.

Domain and SQ^a^		P_O_(extraction; %)	P_O_ (response; %)	Contribution from “No information” (%)
Domain 1				
RoB^b^ 1.1		90	70	0
RoB 1.2		70	60	0
RoB 1.3		40	30	0
Domain 2				
RoB 2.1		50	40	0
RoB 2.2		30	30	10
RoB 2.3		60	60	50
RoB 2.4		90	90	100
RoB 2.5		90	90	100
RoB 2.6		80	50	10
RoB 2.7		50	60	40
Domain 3				
RoB 3.1		30	40	0
RoB 3.2		60	40	40
RoB 3.3		30	30	30
RoB 3.4		70	80	70
Domain 4				
RoB 4.1		40	30	10
RoB 4.2		40	30	20
RoB 4.3		10	10	10
RoB 4.4		10	20	10
RoB 4.5		40	40	40
Domain 5^c^				
RoB 5.1		22.22	77.77	0
RoB 5.2		33.33	44.44	33.33
RoB 5.3		55.55	55.55	44.44

a SQ: signaling question.

b RoB: risk of bias.

c The LLM evaluation for domain 5 was conducted on 9 randomized controlled trials (RCTs), as one lacked a trial registry.

## Discussion

### Visual Placards

According to a study by Dhrangadhariyaet et al [28], 2 factors contributed to low F1 IAA in annotating text spans for SQs: a lack of guidance on annotation granularity and inconsistent annotation locations. Some annotators marked entire paragraphs, while others selected only the most informative text. Our placards address this by specifying whether to annotate a phrase, sentence, or sentences. Additionally, annotators often drew evidence from different parts of the text for the same SQ, lowering agreement. Our placards now restrict annotations for certain SQs to specific sections, such as “Methods,” “Results,” or “Flowcharts.” Flowcharts and tables are designated as the lowest priority for all SQs except 1.3 due to ML models’ difficulty in interpreting them. For example, although SQ 3.1 information appears in the flowchart, annotators are directed to the “Results” section, which better supports ML training.

The visual placards aimed to reduce RoB 2 subjectivity, particularly for SQs with insufficient information for risk judgment annotation. For example, in one trial, both annotators selected “71 allocated routine services, 67 allocated intervention service, 69 assessed at 8 weeks, 64 assessed at 8 weeks” from the PRISMA (Preferred Reporting Items for Systematic Reviews and Meta-Analyses) flowchart to answer SQ 3.1 [50], but one responded “Yes” while the other chose “No.” This question asks if outcome data were available for nearly all randomized participants but lacks a clear threshold. To standardize responses, we introduced a 95% threshold in the placard (Figure 5).

### IAA Measure

#### Text-Span Agreement

F1 agreement at the text span level improved for 10 of the 22 SQs after implementing visual placards. The agreement increased as placards clarified whether to annotate a phrase, sentence, paragraph, or entire table. SQ 1.3 saw the largest increase (70.46 percentage points) due to instructions to mark the entire patient characteristics table. Previously, annotators only marked portions, leading to variability based on what they noticed first. For SQ 1.1, F1 improved by 30 points with instructions to prioritize marking comprehensive information on the randomization method in the Methods section, which offers a detailed description. Previously, annotators marked evidence variably, only including “randomized controlled trial” phrases without checking the randomization method. Placards clarified that “Yes Good” required detailed evidence of randomization. Similarly, SQs 2.1 and 2.2 saw increased IAA due to clear guidance on marking both intervention and placebo descriptions, resolving prior inconsistencies where only one of the two was marked.

While the agreement drastically increased for certain SQs, it remained at 0 for some SQs (2.3, 2.4, 2.5, 2.7, 3.2, 3.3, 3.4, 4.2, 4.5, 5.1, and 5.3), aligning with feedback on these SQs, which indicated high subjectivity, difficulty, and limited information. This suggests that subjectivity and data scarcity in RCTs challenge consistent annotation.

#### Response Option Agreement

Disagreements in response judgments stemmed from 2 main issues. Most disagreements (82.85%) occurred when one annotator provided a response while the other marked “No Information” due to no annotation. Agreements were also split between cases where both annotators labeled a text span similarly and cases where neither annotated, defaulting to “No Information.” Therefore, a considerable chunk of both agreements and disagreements came from “No Information.” This highlights the impact of “No Information” judgments, emphasizing the potential benefit of using high-quality journals to reduce these instances and improve annotation comprehensiveness.

SQs (1.1, 2.1, 2.5, 2.6, 3.1, and 4.3) with adequate agreement (>0.60 κ_PABAK_) showed fewer “No Information” judgments, aligning with feedback that these questions had “normal” or “very high” information availability and low subjectivity. In contrast, SQs with low or negative agreement had more disagreements, leading to lower κ_PABAK_ scores.

**Figure 8.**
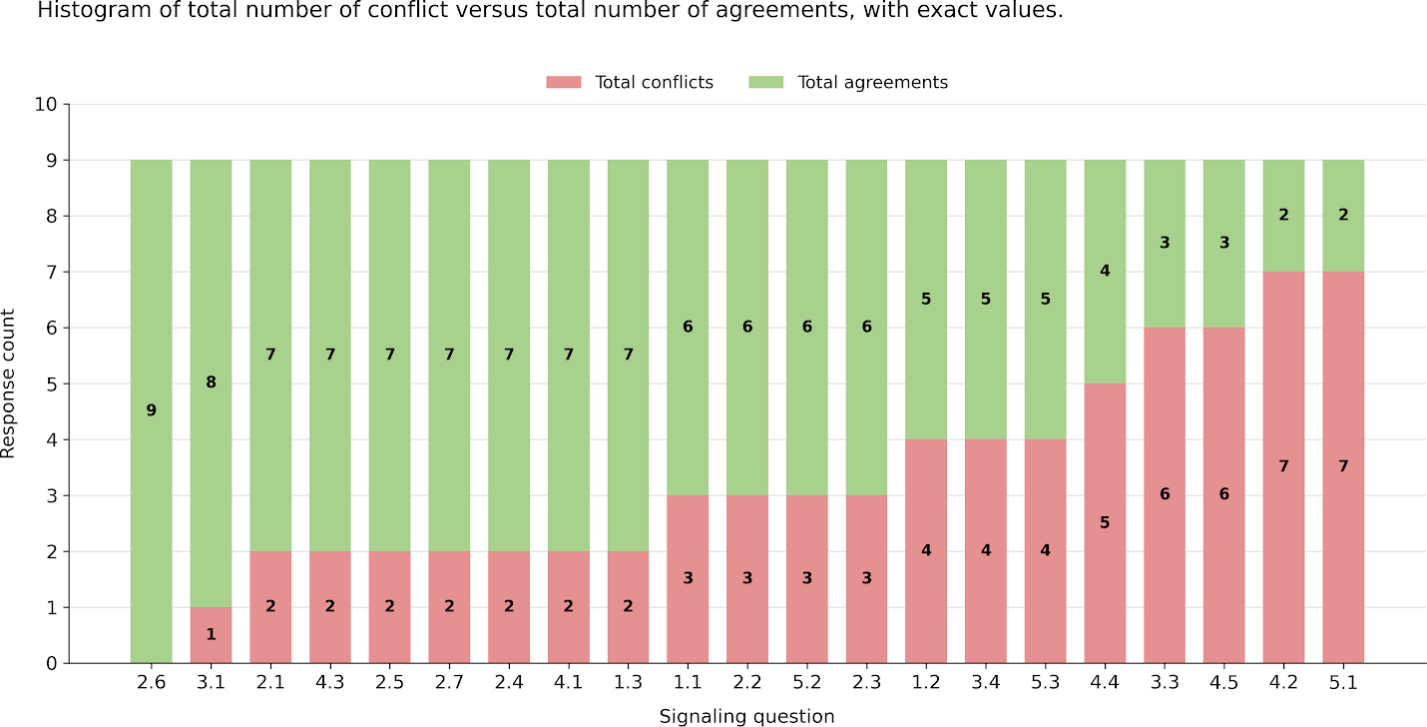
The histogram of total number of conflicts and total number of agreements in the subset of randomized controlled trials (RCTs) used to calculate prevalence and bias adjusted κ.

Negative κ are unlikely to occur in practice, but two SQs (4.2 and 5.1) had negative κ_PABAK_ values due to frequent disagreements (in 7 of 9 documents), indicating strong disagreement among raters. The reason for the lower κ_PABAK_ for these questions was disagreements over 7 of the 9 annotated documents, which were the highest number of disagreements in the subset RCTs used for κ calculation (Figure 8). To note that these agreements came from “No Information” judgments. Though the κ_PABAK_ values for these SQs are considerably smaller than –0.10. Such values (<–0.10) suggest that the collected data may not be meaningful for these questions [41].

Domain 5 had nearly zero agreement, likely due to the complexity of assessing it, which requires annotators to reference both the RCT and its trial registry and then summarize the information. Zero IAA also stems from theoretical questions like SQ 3.4, which evaluates whether missing outcome data is linked to true outcome values, and SQ 4.5, which assesses whether outcome assessment was influenced by knowledge of assigned intervention. These questions require hypothetical judgments rather than direct evidence, increasing subjectivity. Since these aspects are often not explicitly covered in trial documentation, annotators were instructed to mark outcome and outcome measurement descriptions to provide a foundation for judgments and ensure relevant annotations for evaluating LLM.

There were certain aspects where question subjectivity led to low agreement. For SQ 2.4, which asks if deviations from intended intervention might affect outcomes, responses are inherently subjective, as assessors may differ on what they consider impactful. Limited information on deviations further complicates accurate judgment. Only 2 of the 9 RCTs had SQ 2.4 annotations, and only 1 annotator marked them, resulting in no text span agreement. SQ 3.3 requires assessors to judge whether missing data are related to the true outcome, which can be challenging to determine objectively. Annotating evidence is further complicated by the need to link missing data reasons to outcome values, such as “fatigue” in physiotherapy studies. If, for example, patients missed follow-up due to fatigue, this connection should be annotated. However, RCTs rarely include this level of detail. Placards instead advised annotators to mark outcome descriptions to support judgment, but 1 annotator did not follow this guidance, leading to zero text span agreement. Such cases indicate a need for extended training, conflict resolution rounds, and placard improvement.

### LLM Evaluation

#### Direct Versus Indirect Responses

LLM gave direct responses (“Yes,” “No,” or “No Information”) for some questions and responded indirectly for others. For instance, in an RCT by Gleason et al [51], it directly answered SQ 1.1, stating, “Yes, the allocation sequence was random,” and extracted relevant supporting text. However, for SQ 5.2, instead of stating “No information was present to make a judgment,” the LLM extracted a candidate paragraph along with the following text: “However, the study authors did not provide information on whether the numerical result being assessed was selected based on the results from multiple eligible outcome measurements within the outcome domain.” This response implied that it found “No Information” for SQ 5.2.

#### Domain-Specific Findings

The LLM performed well in extracting information on participant randomization and allocation concealment for SQs 1.1 and 1.2, resulting in good $P_{O}\left(Extraction\right)$ agreement. However, it sometimes reached the correct response judgment, leading to a higher $P_{O}\left(Response\right)$, without fully extracting the required evidence. In an RCT by Stuck et al [52], the LLM extracted an irrelevant passage to answer SQ 1.1 with “Yes Good” (low risk of randomization bias) even though the extracted sentence did not contain information about the randomization method. For SQ 1.3, lower agreement was observed between the expert and LLM, as the LLM often relied on text rather than tables, where critical data were found. Text in tables is read by ChatPDF, but it might have problems correlating the correct rows and columns, leading to a distorted understanding of the tables. For Stuck et al’s [52] RCT, both the expert and LLM answered SQ 1.3 with “No Good,” but the expert used a table as the evidence to answer the question, while the LLM used the text evidence not found in the table.

In domain 2, approximately 40% of $P_{O}\left(Response\right)$ agreement was due to “No Information,” indicating a lack of information in RCTs for SQs 2.4 and 2.5. In an instance, LLM evaluation led to the identification of an incorrect label from the expert. To elaborate, for SQ 2.6 in Hassett et al’s [53] study, the expert had annotated an incorrect part of the text to answer the question with “Yes Good.” LLM correctly extracted information about the intention-to-treat analysis, which led to correcting the final annotation in RoBuster.

In domain 3, bias assessment subjectivity emerged. A lenient assessor will judge a risk of bias as low in comparison to a stringent assessor judging a bias risk as high for any SQ, but it is more pronounced in subjective SQs. In Thorndike et al’s [54] RCT, both the LLM and the expert used the same evidence, “...104 (82%) were randomized in January 2011. Five residents withdrew during Phase 1, and 99 continued participation in Phase 2...,” to answer SQ 3.1. However, the LLM rated it as “Yes Good,” whereas the expert rated it as “No Bad,” following a strict rule that any outcome data missing for over 5% of participants indicates high bias risk, regardless of other study details. For SQ 3.2 in RCTs [55,56], the LLM was more lenient than the experts, extracting information about the sensitivity analysis carried out by the study authors to account for missing outcome data. However, the RCT authors were not explicit that there was no bias due to missing outcomes data. For SQ 3.3, LLM judged “No Information” 9 out of 10 times. It questions whether the missingness in outcomes depended on its true value and is quite subjective because the assessor needs to contemplate the reasons for missingness for a particular outcome and whether the true value could have affected the missingness. For example, they need to assess whether the fact that data on falls is missing can be attributed to the actual occurrence of falls in the study.

The agreement was lower in domain 4, despite outcome measurement data being present, as it often extracted nontargeted outcomes due to simple prompts. The annotators assess bias pertaining to the predecided target outcome and not all the outcomes reported in the trial. However, the information about the target outcome being assessed was not available in the simple prompts used to test LLMs. Consequently, the LLMs extracted information that did not necessarily pertain to the target outcome but rather to other outcomes reported in the trial. Enhancing prompt specificity, potentially with chain-of-thought or tree-of-thought styles, could improve the LLM’s performance in RoB assessment [57].

In sum, while the LLM shows potential for automating RoB span extraction, further optimization is needed for handling subjective SQs, lack of information, and tailoring prompts to specific bias contexts.

### Limitations

The scale of our annotations is limited to 41 RCTs due to financial constraints, which restricted our ability to hire expert annotators. Furthermore, we acknowledge that performing double-coding on a subset of 9 out of 41 RCTs (about 22% of the corpus) restricts the statistical precision of our reliability estimation. While this subset was key in identifying and resolving discrepancies during the annotation process, the resulting IAA metrics should be interpreted as indicative rather than exhaustive.

Our focus is limited to physiotherapy and rehabilitation as we relied on voluntary experts only from these domains. This limitation was necessary to have annotators proficient in the domain, as expanding to other domains could compromise the validity of expert judgments [6]. Though our approach restricts the broader applicability of the corpus, the annotations provided are robust, reflecting thorough assessment by experts within available resources. The sampling was restricted to articles published after 2010 to ensure consistency with modern CONSORT (Consolidated Standards of Reporting Trials) reporting practices. To facilitate open sharing and publication of the annotated corpus, we included only articles available under the CC0 license. While these criteria were necessary for methodological consistency and licensing requirements, they may limit generalizability to RCTs published prior to 2010, to other clinical domains, or to studies published in non–open-access venues.

The low IAA observed stems from the subjective nature of RoB 2, which requires evaluators to interpret nuanced details across various domains. While we have implemented visual placards to aid standardization, these improvements cannot fully resolve the underlying subjectivity in the tool’s design. RoB 2, despite offering more structured guidance than its predecessor, still relies on subjective judgment in answering SQs, particularly in complex domains like deviations from intended interventions. This subjectivity is not unique to our study, but is a fundamental challenge in RoB assessments and is recognized in previous research. Given the interpretative nature of RoB assessments, it is not feasible to entirely eliminate these differences. While we continue to explore methods for improving standardization, it is crucial to recognize that the inherent subjectivity in RoB 2 will always impact IAA to some degree.

Our LLM evaluation at that time was restricted to using PDFs, limiting platform compatibility and model selection. Specifically, Google Bard is a freely available tool that interacts with PDFs, but we observed that the Bard results were less deterministic than ChatPDF. Another drawback was associated with the prompts used. Our prompts were also relatively simple and lacked specificity regarding target outcomes, resulting in general responses rather than targeted bias assessments. In retrospect, we should have attached the detailed trial protocol and the statistical analysis plan to each clinical trial before annotation and LLM evaluation. This limitation will be addressed in future expansions of the corpus by adding both the protocol and analysis plan to the RCTs for annotation.

While we have provided a detailed account of our LLM evaluation methodology, we acknowledge the importance of adopting structured reporting frameworks, such as the recently published STAGER (Standards for Transparent And Generative Evaluation and Reporting) checklist, for studies involving generative artificial intelligence. Future studies could benefit from adhering to such guidelines to ensure greater methodological rigor and transparency in reporting [58].

### Conclusions

We present RoBuster, a new publicly available corpus of 41 full-text annotated RCTs with detailed RoB spans across 22 bias questions. This corpus and our rigorously developed annotation guidelines address a gap in resources for evaluating RoB text span extraction using ML. RoBuster includes fine-grained bias spans and annotator decisions, providing a benchmark for assessing LLM performance against human bias assessment. Created through collaboration between bias assessors and natural language processing experts, RoBuster can enhance automated bias assessment and support systematic literature review systems.

Our work reaffirms the complexity of bias assessment and the need for robust guidelines to improve IAA in both assessment and annotation. Challenges stemmed from variation in RCT reporting standards, leading to low information availability and the subjectivity inherent in RoB 2. Future work will focus on refining our visual placards, expanding RoBuster with more annotated texts, and using the placards to train new bias assessors.

## Supplementary material

10.2196/55127 Multimedia Appendix 1 Documentation of signaling questions and corresponding annotation instructions used in this study.

10.2196/55127 Multimedia Appendix 2 Annotators’ feedback on the risk of bias assessment and annotation for the randomized controlled trials in RoBuster corpus.

10.2196/55127 Multimedia Appendix 3 The visual instruction guidelines in PPTX format. These placards are accompanied by Multimedia Appendix 1.

10.2196/55127 Multimedia Appendix 4 An .xlsx file containing the large language model (LLM) evaluation, detailing the text extraction and response of the LLM and the corresponding text span and response judgment labeled by the expert annotator for all the signaling questions.

10.2196/55127 Multimedia Appendix 5 The RoBuster dataset in .tsv format.

## Abbreviations

AMSTAR: A Measurement Tool to Assess Systematic Reviews
CONSORT: Consolidated Standards of Reporting Trials
EPOC: Effective Practice and Organization of Care
IAA: interannotator agreement
LLM: large language model
ML: machine learning
PABAK: prevalence and bias adjusted κ
PAWLS: PDF Annotation With Labels and Structure
PRISMA: Preferred Reporting Items for Systematic Reviews and Meta-Analyses
RCT: randomized controlled trial
RoB: risk of bias
SQ: signaling question
SR: systematic review
STAGER: Standards for Transparent And Generative Evaluation and Reporting
