# Supplementary material for: RoBuster—Corpus Annotated With Risk of Bias Text Spans in Randomized Controlled Trials in Physiotherapy and Rehabilitation: Corpus Development and Annotation Study
Source: JMIR Form Res. 2026 Apr 27;10:e55127. doi: 10.2196/55127 (PMC13120535; doi:10.2196/55127)
Supplement: Multimedia Appendix 1 [file formative-v10-e55127-s001.docx]

# Appendix 1 for “RoBuster: A Corpus Annotated with Risk of Bias Text Spans in Randomized Controlled Trials”

This document is accompanied by the file containing visual instruction placards (RoB_guidelines_placards.pptx). We will refer this file as placards.

## Generic Annotation Guidelines

This section provides a comprehensive explanation of the structure of the visual instructional placards and the general guidelines for using them effectively. The visual placards serve as a guide for annotators and are structured in a specific way to ensure consistent and accurate annotation of the Risk of Bias (RoB) questions. Each placard begins with a title that clearly indicates which signalling question it provides annotation instructions for. Following the title, a flowchart is provided to guide annotators through the process of annotating each RoB question based on the revised Cochrane Risk of Bias tool 2 for assessment of Randomized Controlled Trials (RCTs) (referred to as RoB 2 hereafter). The flowcharts consist of several components, including:

1. Diamond: Each flowchart contains diamond-shaped symbols that prompt annotators to search for specific information and ask decision-based questions. If the required information is found, instructions are provided on how to annotate it or guidance is given to proceed to the next diamond.
2. Parallelogram: The output answer to the decision-based question is represented by a parallelogram-shaped symbol. Annotators are instructed to assign a label associated with this symbol if the requested information is found. These labels align with the labels in the annotation scheme described in the main paper.
3. Arrows: Color-coded arrows unilaterally flank the diamonds. These arrows provide instructions to annotators to first search for the required information in the full-text section suggested by the green arrow (e.g., Results or Methods section). If the information is not found in the green-coded arrow, they are instructed to check for it in the section suggested by the yellow-coded arrow (e.g., a table or a flowchart). The sections suggested in red arrows with a cross mark denote the information requested by the diamond should not be found in these sections.
4. Thought bubble: Bubble-shaped text boxes, referred to as thought bubbles, contain comments that provide additional information, explanations, or instructions about specific steps or elements in the flowchart. These boxes are primarily colored green but may also be red.

The placards also include additional instructions in the bottom left corner, specifying whether annotators should annotate full sentences or only phrases. In the following subsections, detailed annotation guidelines are provided for each signalling question (SQ), using the instructional placards developed in consultation with RoB assessment and natural language processing experts. It's important to emphasize that these visual instructional placards should be used in conjunction with the original detailed RoB 2 guidelines. The aim of our placards is to aid the text annotation rather than replace the original RoB 2 guidelines [1].

## Annotation Guidelines for RoB Domain 1

The first risk domain in the RoB 2 tool assists assessment of the “bias stemming from the randomization process” focusing on the three major aspects: I. random sequence generation, II. allocation concealment of the enrolled participants, and III. was the randomized adequately implemented in practice. Proper randomization is essential to RCTs and it ensures that participants are assigned to intervention groups in an unbiased manner enabling an unbiased comparison of intervention outcomes across all groups [2].

## Signalling Question 1.1

Signalling question 1.1 “Was the allocation sequence random?” assesses whether the allocation of participants to different interventions groups was done in a random manner. If the allocation sequence is generated randomly, this reduces the bias risk, as it ensures that the allocation is not influenced by the researchers' preferences or the participants' characteristics. Some of the good random allocation methods include simple randomization (coin flipping, random number generator), stratified randomization, blocked randomization, cluster randomization, and adaptive randomization [1]. If the allocation sequence was not generated using a proper randomization method, this increases the bias risk in the study. For e.g., if the allocation sequence was generated using a non-random method, such as alternating assignment or assignment based on participant characteristics, this could introduce bias into the study. In fact, improperly generated allocation sequence or inadequately concealed randomization sequence were associated with larger intervention estimates in RCTs [3].

Follow the Flowchart in placards slide 4 for annotation instructions of the SQ 1.1: “Was the allocation sequence random?”. Following the above explanation, the first diamond in the flowchart instructs the annotators to identify the randomization method for allocation sequence, and if a proper randomization method is found, mark the method description with “1.1 Yes Good”. If the text describes a bad or improper method of randomization, mark the text with “1.1 No Bad”. This description should be a few words or a phrase and not a complete sentence. If you did not find any information about the randomization methodology, but if the system finds relating terms like “random”, “randomized trial” or phrases like “we did random allocation” are automatically detected, then such phrases will be marked with the label “1.1 No Information”. The information about random sequence allocation can be found in the methods section (priority) or the abstract (second priority). Notice that for this signalling question, the annotators are required to mark phrases rather than full sentences [1, 4].

## Signalling Question 1.2

The signalling question “Was the allocation sequence concealed until participants were enrolled and assigned to interventions?” focuses on assessing the bias risk related to allocation concealment in a study. In RCTs, the allocation sequence determines the order in which participants are assigned to each intervention group. Adequate concealment of the allocation sequence refers to the process of keeping the sequence hidden from those who are responsible for enrolling and assigning participants to intervention groups. It helps prevent the possibility of selection bias, which can occur if those enrolling participants in the trial have knowledge of the allocation sequence and can manipulate the assignment of participants to intervention groups based on that knowledge[3]. For example, if the allocation sequence is known to the investigators or coordinators, they may selectively enroll participants who are more likely to benefit from a particular treatment, or they may assign participants to one treatment group over another based on personal biases.

Follow the Flowchart in placards slide 5 for annotation instructions of SQ 1.2: “Was the allocation sequence concealed until participants were enrolled and assigned to interventions?”.

Based on the explanation given earlier, the first diamond in the flowchart guides annotators to identify the method of allocation concealment. If a proper allocation concealment method is identified but lacks certain details necessary for making a judgment, it should be marked with “1.2 No Information”. For instance, if the description mentions the use of sealed envelopes but does not specify whether they were opaque, it would be marked as “1.2 No Information”. On the other hand, if enough information is provided to make a judgment, it should be marked with “1.2 Yes Good”. Good methods of allocation concealment include central randomization, sealed opaque envelopes, and using online randomization tools that offer to securely generate and conceal the allocation sequence [1]. If an improper method of allocation concealment is utilized, then mark the method with “1.2 No Bad”. The improper methods of allocation concealment include using an open list, a predictable sequence like alternating using the date of birth or other characteristics or using the participant's choice. The primary sources for finding information about allocation concealment are the methods section (first priority, as shown in the green arrow) and the abstract (second priority, as shown in the yellow arrow) [1, 5]. For this signalling question, annotate a whole sentence if the whole sentence corresponds to answering this question and annotate a phrase if only a part of the sentence does concern answering the question. For example, annotate the whole sentence “A computer generated random sequence list was generate with two strata and random block size with sizes of two to eight” because annotating the whole sentence is necessary to answer the question. For this next example, annotate only the second part of the sentence, “A random sequence was generated with a software (stratified for site and with random block sizes), and the allocation was concealed with sealed opaque envelopes.”

## Signalling Question 1.3

The signalling question “Did baseline differences between intervention groups suggest a problem with the randomization process?” of the RoB 2 tool is related to the potential for bias arising from differences in baseline characteristics between intervention groups that are not related to chance. This suggests that there may be a problem with the randomization process. Even with good randomization, chance imbalances in baseline characteristics can occur leading to an ineffective randomization. For example, if the treatment group has significantly more female participants, older participants, or participants with more severe disease at baseline compared to the control group, this may suggest that the randomization process was not effective or that there was a problem with the allocation sequence. This can lead to biased estimates of treatment effects and decrease the internal validity and reliability of the study[6].

Follow the Flowchart in placards slide 6 for annotation instructions of the SQ, “Did baseline differences between intervention groups suggest a problem with the randomization process?”. The information to assess this question could be found in the table describing the demographic and clinical baseline characteristics of study participants. At this point, the first diamond requests the annotators to mark the full table instead of one or two characteristics, as extracted table text from PDFs lack the inherent structure necessary for training machine learning models. Therefore, it will be difficult to use the extracted table text to train the machine learning models effectively. If the table for baseline patient characteristics shows excessive similarity or dissimilarity in the baseline characteristics that ARE NOT compatible with chance, then mark the full table as well as table caption with the label “1.3 Yes Bad” otherwise, mark the full table and the table caption with the label “1.3 No Good”. In some cases, this table is missing, but there could be text descriptions that could hint towards baseline differences that could act as confounding factors. For instance, consider the following sentence, “Because baseline ODI differences were a potential confounding factor, an adjusted multiple linear and logistic regression analysis was performed for each continuous and categorical outcome measure...” [7]. If you find any such information in the full text, mark it with the label “1.3 No Information”. The goal is to capture any relevant information in the full text or table that can help determine if baseline differences between intervention groups indicate a problem with randomization.

## Annotation Guidelines for RoB Domain 2

The second risk domain in the RoB 2 tool is referred to as “bias due to deviations from intended interventions”. In RCTs, it is essential to ensure that participants in a study are assigned to the intended interventions accurately and that the interventions are administered as planned. However, there can be deviations from the intended interventions when participants do not receive the assigned treatment. This can happen for various reasons, such as non-compliance by participants, crossovers between treatment groups, or missing data due to dropout or loss to follow-up. In fact, trials that deviated from the intention to treat analysis showed larger intervention effects [8].

Before detailing the annotation instructions for this domain, we clarify the difference between deviations from intended interventions and dropouts from the trial. Deviations from intended interventions are any unintentional changes that could have occurred in the treatment under investigation in the trial. For example, participants may not have complied with the study protocol, or there could have been problems with the delivery of the intervention. In contrast, dropouts refer to participants who withdrew from the study or were lost to follow-up. Dropouts can potentially introduce bias into a study if the characteristics of the participants who dropped out are different from those who completed the study. High dropout rates can also affect the statistical power of the study and its ability to draw valid conclusions. Although both deviations from the intended intervention and dropout rates can potentially introduce bias into a study, they are not the same thing and should be evaluated separately when assessing in a study [9, 10].

## Signalling Question 2.1

The SQ 2.1, which evaluates whether participants were aware of their assigned intervention during the trial assesses whether the clinical study performed proper blinding or masking of the trial participants. Blinding or masking of participants is important to minimize the bias risk due to participants' knowledge of their assigned intervention. When participants are not blinded, they could potentially modify their behavior, expectations, or reports of outcomes, consciously or unconsciously, based on their knowledge of the assigned intervention, leading to a biased estimate of the treatment effect. Proper blinding of participants involves concealing the intervention to ensure that the participants do not know which intervention they received [10, 11, 12]. Proper blinding of participants in physiotherapy and rehabilitation domains is challenging due to the nature of the interventions. In these domains, participant blinding may not be possible because the treatment and control pairs involve a clear difference in the type, administration or frequency of therapy being delivered, such as exercise interventions, behavioural interventions, educational interventions, and mind-body interventions [13, 14].

Follow the Flowcharts in placards slide 8 and 9 for annotation instructions of the SQ 2.1. Following the above explanation, the first diamond in the flowchart asks the annotators to find text descriptions stating that the participants could not distinguish (unaware/blinded) between the assigned interventions (the main intervention and the control intervention). If such description is found, then annotate it and follow the connector line towards the decision “yes”, find the sentences that describe intervention and placebo administration and annotate both the descriptions – the first part where intervention administration is described, and the second part where proper participant blinding via a placebo is described. Annotate these parts with the label “2.1 No Good”. Both these administration descriptions can be found either in adjacent sentences or far apart in the same or different paragraphs within the same section. Annotators are likely to find this information in the methods section and not in the Results section. If text parts describing the intervention and placebo administration are not found, but there are phrases in the title or abstract indicating that the study is “placebo-controlled”, “single-blind”, “double-blind”, then mark these phrases with the label “2.1 No Information”.

Going back to the first diamond, if the annotators did not find any information stating that the participants were unaware of the assigned intervention, they are asked to go to part II of the placards (see Flowchart 5). If the annotators find that the study is “open label” or found the sentence(s) mentioning that there was no blinding, or participants were aware, or could not be blinded, or something along the lines of the sentences mentioned below, then annotate such sentences with the label “2.1 Yes Bad”. For example, 1) “Blinding of trial participants and the trial coordinator was not possible due to the nature of the interventions…”, and 2) “...we wanted to evaluate the commonly used methods that often involve parents’ actions, and blinding of parents to participants, care providers, or assessors was not possible due to the nature of the intervention.” [14, 13]. If the information about the study being “open-label” or participants being aware is not found, but the annotator thinks that for the intervention-comparator (IC) pair, it is not possible to achieve blinding using the IC pairs then annotate the phrases or words that give information about IC (names of intervention and comparator) with the label “2.1 Probably Yes Bad”. Blinding information can be found either in the methods section or the abstract.

## Signalling Question 2.2

While the previous signalling question assessed participant blinding, this SQ, “Were the carers and people delivering the interventions aware of the participants' assigned intervention during the trial?” evaluates whether the carers and individuals administering the interventions were aware of the participants' assigned interventions during the trial. If carers and treatment administrators are not blinded, them being aware of the assigned interventions could influence their behavior, interactions with participants, and delivery of the interventions. This can potentially introduce bias and alter the study outcomes [15].

To provide clearer guidance, please follow Flowchart 6 for annotation instructions related to the signalling question, "Were the carers and people delivering the interventions aware of the participants' assigned intervention during the trial?" In the explanation above, the first diamond directs annotators to search for sentences that describe the procedure for administering the intervention under investigation and the placebo. If such sentences are found, the annotators should annotate both parts of the text: the section describing intervention administration and the section explaining how carers and administrators were blinded using a placebo or sham. If the sham is considered a good blinding procedure, label these text parts with “2.2 No Good”. For example, [16] describes intervention administration as “This RCT used a 5-week, double-blind, parallel groups design”, and carer blinding as “Treatment was administered by an RA who was blinded to assessment results and study hypotheses”. If sentences of this nature are not found, go to the connecting line indicating “No” and move to the next diamond. In this diamond, annotators are instructed to search for sentences that either explicitly state or strongly imply that the carers and administrators could not differentiate between the intervention and placebo. Additionally, check if the study's title or abstract mentions terms like “single blind”, “double blind”, or “placebo-controlled”. Mark such sentences or phrases and label them as “2.2 No Information”. In cases where proper carer blinding was not described or it is explicitly mentioned that carer blinding was not possible, the sentences identified using the first diamond should be marked with the label “2.2 Yes Bad”. An example of this is, “...we wanted to evaluate the commonly used methods that often involve parents' actions, and blinding of parents to participants, care providers, or assessors was not possible due to the nature of the intervention” [13].

## Signalling Question 2.3

The signalling question 2.3, “Were there deviations from the intended intervention that arose because of the trial context?” aims to assess whether any deviations that occurred during the trial were due to factors related to the trial context. The intended intervention refers to the specific treatment or intervention that is planned to be delivered to participants. Deviations from the intended intervention can occur for a variety of reasons, such as practical challenges, implementation issues, or unexpected circumstances within the trial context [17]. This question focuses on assessing whether these deviations from the intended intervention were due to factors directly related to the trial rather than factors outside the trial. It helps evaluate the extent to which the trial protocol was followed and whether the trial results could have been influenced by these deviations.

Follow the Flowchart in placards slide 11 for annotation instructions for this SQ. In the flowchart, the first diamond prompts annotators to search for information regarding the number of deviations from the intended intervention. If no percentage or absolute number of deviations is identified in the text, no annotation is necessary, and the document will be automatically marked as “2.3 No Information” for this signalling question, following the “No” arrow. If the percentage of deviations is identified, annotators should annotate the full sentence where this information is found. They should then proceed to the next diamond in the flowchart, following the “yes” flow, and the next diamond instructs the annotator to assess whether these deviations occurred due to the trial context. If the deviations are determined to be due to the trial context, the annotated sentence(s) should be marked as “2.3 Yes Bad”. If the deviations are not attributed to the trial context, they should be marked as “2.3 No Good”. For example, “Two patients in the control group receiving physiotherapy (usual care) erroneously attended the PT-led education and exercise programme after...” [18]. Additionally, if annotators identify the reasons for their choices of “no good” or “yes bad”, they should mark the sentences explaining their rationale. It is important to note that a second annotation may be required if there are supporting sentences for the reason behind the choice. This second annotation allows for capturing additional information. This information can be found in the text of the Results section, specifically within the allocation or recruitment paragraphs (indicated by the green-coded arrow on the diamonds). Only if the information is not found in the Results section, should the annotators check the flowcharts or their captions for any relevant details. The judgment regarding the trial context and deviations can be subjective, and it is left to the reviewers' discretion once they have access to the deviation information. Ultimately, the review of deviations and trial context is subjective, but the annotated information (percentage or number of deviations) can be used to train machine learning models.

## Signalling Question 2.4

In RCTs, deviations from the planned study protocol may occur for various reasons, such as the participant not following the intervention as intended, the trial site not adhering to the study procedures, external constraints, or trial discontinuation. These deviations are known as “protocol deviations” [19, 20]. The question “Were these deviations likely to have affected the outcome?” refers to whether the protocol deviations had the potential to influence the study outcomes. If the identified deviations (identified using the previous questions) are determined to have a substantial likelihood of affecting the study outcomes, it suggests a higher risk of bias [21]. In contrast, if the deviations are unlikely to have influenced the outcomes significantly, it indicates a lower bias risk. Assessing the impact of deviations on the outcomes requires a thorough understanding of the study design, interventions, and potential sources of bias. It may involve considering factors such as the timing, frequency, and magnitude of the deviations, as well as their relevance to the study objectives and population under investigation and can ultimately be a subjective judgment.

For annotation instructions for this signalling question follow the Flowchart in placards slide 12. Before proceeding with the annotation, ensure that the previous question was not marked as “2.3 No Good” (in this case either no deviations were identified, or they were not significant). If it was, no annotation is required for this question. Otherwise, follow the instructions below. The first diamond prompts the annotators to identify the reasons for deviations and, if found, they mark it, then follow the connector path of “yes”and judge whether these deviations could affect the outcome. Using the annotated reasons hypothesize whether these could cause some deviation, then mark them as “2.4 Yes Bad” and “2.4 No Good” otherwise. Annotate full sentences for this question. The reasons could be found either in the Results section (main priority) or the Methods section (second priority). If the reasons for deviations are not found, then the question is assumed to have “No Information”.

## Signalling Question 2.5

This signalling question 2.5, “Were these deviations from the intended intervention balanced between groups?” aims to assess whether any deviations from the intended intervention were similar in frequency and severity across the different intervention groups (i.e., intervention group and control group) or whether one group had a greater number or severity of deviations than the other. Balanced deviations between groups are important for maintaining the internal validity of the study. If one group had a higher number (or severity) of deviations from the intended intervention than the other, it could potentially introduce bias and confound the results of the study. Therefore, it is important to assess whether deviations from the intended intervention were balanced between the groups when evaluating the risk of bias in an RCT [22].

For annotation instructions for this signalling question, “Were these deviations from the intended intervention balanced between groups?” follow the Flowchart in placards slide 13. The first diamond in the flowchart instructs annotators to search for quantities or numbers indicating deviations from the intended intervention. It is important to distinguish between deviations and dropout rates. Focus on identifying any numerical information that demonstrates deviations from the intended intervention.

If the deviations are balanced between the groups, mark them as “2.5 Yes Good”. If they are not balanced, mark them as “2.5 No Bad” [23]. In cases where no quantification of deviations is found, but the Discussion section mentions that the deviations were not balanced, mark that text in the discussion as “2.5 No Bad”. For example, “In the standard care group, the use of corticosteroids in 14 patients (9.4%) was considered a protocol deviation, and the...” should be marked as “2.5 No Bad. In contrast, “There were only few missing data because of premature withdrawal, and they were balanced in numbers across the 2 groups with similar reasons for missing data.” should be marked as “2.5 Yes Good” [24]. If no text is marked, the document will receive a “No Information” label for this signalling question. Ensure that the annotation reflects the presence or absence of balanced deviations and consider both quantitative information and relevant discussion text to make an accurate annotation.

## Signalling Question 2.6

The signalling question “Was an appropriate analysis used to estimate the effect of assignment to intervention?” evaluates whether the analysis performed to estimate the effect of assigning participants to interventions in the study was reliable and valid.

Participants' assignment to intervention is key to unbiasedly determining the causal relationship between the intervention and outcome. To ensure the validity and reliability of the study results, it is essential to use appropriate statistical methods and analytical techniques when estimating the effect of assignment to interventions.

So, this question assesses the risk of bias associated with the analysis methodology applied. An appropriate analysis involves using valid statistical tests, appropriate control for confounding variables, and properly considering potential sources of bias or confounding [25].

For annotation instructions regarding the signalling question “Was an appropriate analysis used to estimate the effect of assignment to intervention?” please refer to Flowchart in placards slide 14. To clarify, the first diamond in the flowchart guides annotators to identify whether the study used ITT (Intent-to-Treat) or modified ITT analysis. ITT analysis is a method used to assess the effectiveness of an intervention by analyzing outcomes based on participants' initial assigned treatment group, regardless of whether they received the assigned treatment or not. If the annotators find information indicating the use of ITT analysis, they should mark and label the corresponding sentences as “2.6 Yes Good”. Alternatively, if the study did not use ITT analysis and employed methods like naive “per-protocol” analysis or “as treated" analysis to handle deviations from intended interventions, annotators should mark and label the relevant text as “2.6 No Bad”. To locate this information, annotators should prioritize checking the Methods section, followed by the Results or Discussion section. If no information regarding ITT analysis, naive “per-protocol” analysis, or “as treated” analysis is found, it is assumed that no information is available to answer this question in the paper, and it should be labelled as “No Information” [25].

## Signalling Question 2.7

The question “Was there potential for a substantial impact (on the result) of the failure to analyze participants in the group to which they were randomized?” refers to the risk of bias associated with deviations from the intended analysis plan in an RCT. In an RCT, participants are usually randomized to receive either the intervention being tested or a control intervention. The analysis plan is typically based on the assumption that all participants will be analyzed according to the group to which they were randomized. However, there may be instances where participants are not analyzed in the group to which they were randomized, for example, if they dropped out of the study or if they switched groups [26, 27]. This is known as a deviation from the intended analysis plan (not to be confused with deviation from the intended intervention). The question is asking whether this deviation from the intended analysis plan could have had a substantial impact on the study results. If there is a potential for a substantial impact, then there is a high risk of bias. For example, if a large proportion of participants in the intervention group dropped out of the study, but all participants in the control group were analyzed, this could lead to an overestimation of the treatment effect. Similarly, if participants switched groups, this could lead to an underestimation or overestimation of the treatment effect [25].

For annotation instructions for this signalling question, “Was there potential for a substantial impact (on the result) of the failure to analyze participants in the group to which they were randomized?” follow the Flowchart in placards slide 15. The analysis we focus on this question is ITT or intent-to-treat analysis. The first diamond asks the annotators to find the number of people included in the (modified) ITT analysis vs. the number of people randomized to each of the intervention or comparator groups. If this information is found, annotate it and follow the “yes” flow in the flowchart. Now examine whether the percentage of participants excluded from the ITT analysis could have had a negative impact. We also include a hint here, and if more than 5% of randomized participants were excluded from ITT analysis, the annotators mark the text as “2.7 Yes Bad” and “2.7 No Good” otherwise. The first preference to find this information is the Results section. This information is usually found in the flowcharts, but using our current annotation software, we cannot label the text found in images, and we keep labelling flowcharts as a second priority. Therefore, for the sake of simplicity and good agreement, the annotators are requested to mark the flowchart captions. If the information is not found in the results section, mark the text in the table. If the information is to be found nowhere except in a flowchart, mark the caption of the flowchart. However, ensure that the flowchart annotation is your last resort.

## Annotation Guidelines for RoB Domain 3

Domain 3 of the Revised Cochrane Risk of Bias (RoB) 2 tool addresses the potential “Bias due to missing outcome data” in a study. Bias due to missing outcome data refers to situations where the data for some participants are not available for analysis, and this missingness may be related to the outcomes being measured. This domain aims to evaluate whether there is a potential bias in how missing data is handled and reported, which can affect the validity and reliability of the RCT's findings.

## Signalling Question 3.1

The question “Were data for this outcome available for all, or nearly all participants randomized?” assesses the completeness of outcome data in a study. It aims to determine if data for the outcome of interest^[[1]](#footnote-1)^ were collected and available for analysis for a high proportion of the participants who were initially randomized into the study. By evaluating this question, you can assess the risk of bias associated with missing or incomplete outcome data. If the data for the outcome of interest were not available for a substantial number of participants or if there was a high degree of missing data, it raises concerns about the potential impact on the validity and reliability of the study's findings. Missing data can lead to reduced statistical power and imprecise estimates of treatment effects. Adequate availability of outcome data for most randomized participants enhances confidence in the study's results and reduces the risk of bias in this domain [28, 29].

For annotation instructions for this signalling question, “Were data for this outcome available for all, or nearly all Participants randomized?” follow the Flowchart in placards slide 17. Following the above explanation, the first diamond instructs the annotators to check the Results section (priority) and the flowchart (second priority) to identify outcome data at the specific time point of interest. If the annotators find that the outcomes data were available for all or nearly all (at least 95% of the total) participants, mark the text descriptions mentioning it and label it with “3.1 Yes Good”. If the outcomes data were available for less than 95% of participants randomized, then mark this information and label it with “3.1 No Bad”. If no information regarding the availability of outcome data at the specified time point is found, consider the question as having “No Information”. This information is likely found in the Results section of the RCT under consideration, but if not found there, then mark the information in results table. If the information is to be found nowhere except in a flowchart, mark the caption of the flowchart. However, ensure that the flowchart annotation is your last resort.

## Signalling Question 3.2

The signalling question “Is there evidence that the result was not biased by missing outcome data?” evaluates the potential bias introduced by missing data in a study. It aims to evaluate whether there is sufficient evidence to conclude that the study's results were not influenced or biased due to missing outcome data. When answering this question, researchers evaluate whether the missing outcome data “could” have influenced the study's results in a biased manner. Missing outcome data can occur when participants drop out of a study, fail to provide data for certain outcomes, or when data is not collected as planned. The concern is that the missing data may introduce bias if it is related to the treatment being studied or if it differs systematically between the treatment groups [30]. To determine if there is evidence that the result was not biased by missing outcome data, researchers typically assess the amount of missing data and whether it is evenly distributed across different study groups. If there is a significant amount of missing data, especially if it is related to the outcomes being measured, it may increase the risk of bias. Researchers evaluate the strategies employed to handle missing data. This may involve techniques such as imputation (replacing missing values with estimated values) or sensitivity analyses to assess the impact of missing data on the results. To annotate for this signalling question, the annotators should specifically look for the analysis method that was used to handle or correct for the missing outcomes data. This analysis method could be, for example, Multiple Imputation (MI), Inverse Probability Weighting (IPW), Weighted Estimating Equations (WEE), and Pattern Mixture Models (PMM) [31, 32].

For annotation instructions for this signalling question, “Is there evidence that the result was not biased by missing outcome data?” follow the Flowchart in placards slide 18. The first diamond asks the annotators to look for the use of one of these methods: Multiple Imputation (MI), Inverse Probability Weighting (IPW), Weighted Estimating Equations (WEE), and Pattern Mixture Models (PMM). If the use of the analysis method is found, go to the next diamond, which instructs the annotator to identify any method that was used to conduct sensitivity analysis [33]. Did the study do some sensitivity analysis that shows that this change is due to missing outcomes data, or did the different analysis methods used have a minor effect on how the intervention affects an outcome? Then annotate these sentences containing the analysis method and sensitivity analysis and mark them with “3.2 Yes Good”. By conducting sensitivity analyses, researchers can gain insight into the potential impact of missing data on the study's findings. This information helps to address concerns regarding bias and strengthens the overall validity and reliability of the study results. If no sensitivity analysis was done, then mark the sentence containing the method used to handle the missing data and label it as “3.2 No Bad”. The information to answer this signalling question is likely found in the Method section of the RCTs.

## Signalling Question 3.3

The question “Could the missingness in the outcome depend on its true value?” assesses whether the missingness of data is related to the true values of the outcome being measured in a study. By considering this question, you are examining whether there is a possibility that the missingness of outcome data is not random but instead depends on the actual values of the outcome variable. If missingness is related to the true values, it can introduce bias and impact the validity of the study's results [31, 34]. To answer this question, reviewers need to assess whether there is a potential relationship between the missing data and the true value of the outcome. This can be done by examining the reasons for missing data and analyzing whether the missing data is related to other participant characteristics or study factors. For example, if the study assesses fatigue as the outcome of interest and if participants experience fatigue or drop out of the study due to fatigue, the missingness can potentially be related to missing outcome data in a study. If the answer to RoB 3.3 is judged as “low risk of bias” it means that there is little risk that the missing data could have biased the study findings. If the answer is “high risk of bias” it means that there is a significant risk of bias due to missing outcome data, and the study results should be interpreted with caution.

For annotation instructions for this signalling question, “RoB 3.3 Could missingness in the outcome depends on its true value?” follow the Flowchart in placards slide 19. In order for the annotator to answer this signalling question, the first diamond request the annotators to find the reasons for missing outcomes data. If found the reason for missing outcomes data or not, go to the next diamond and which requests you to find, and if found, mark the sentences that mention this outcome in the context of missingness. If this is an outcome that leads to missingness, then label the marked text as “3.3 Yes Bad”. Otherwise, if no such sentences with missingness context were identified, then mark the sentence describing the outcomes assessed in the Methods section and mark the sentence. If this is an outcome that leads to missingness, then label the marked text as “3.3 Yes Bad”. This is a theoretical question, the answer to which could be basically obtained just by knowing what outcomes are being assessed in the RCT.

## Signalling Question 3.4

The question “Is it likely that the missingness in the outcome depended on its true value?” assesses the likelihood of missing outcome data to be related to the actual values of the outcome variable. For annotation instructions for this signalling question, “RoB 3.4 Is it likely that missingness in the outcome depended on its true value?” follow the Flowcharts in placards slide 20, 21, 22, 23, and 24. To answer this question, the annotator should first find the numbers for missing outcome data in both the intervention and control arms at the specified time point. If these numbers are found, check if the percentage of missing outcome data is greater than 5% between randomization and the analysis time point. If the difference exceeds 5%, mark the corresponding description where these numbers are mentioned and label it as “3.4 Yes Bad”, and otherwise label it as “3.4 No Good”. Ideally, a randomized controlled trial (RCT) should mention if the difference between the patients randomized and those whose outcomes were analyzed is greater than 5%. If such information is found, mark it. If there is no explicit text mentioning this, mark the proportions. This information is usually located in the Results section text or possibly in a table. It may also be identified in the flowchart, but the flowchart should be used as a last resort. If the numbers regarding missing outcomes data are not found then, go to the Flowchart 15.

The Flowchart 15 find the reported reasons for missing outcome data, if available, and annotate them. If these reasons indicate that missingness in the outcome DOES NOT depend on its true value, follow the "no" route and label the reason as “3.4 No Good”. If the missingness in the outcome DOES depend on its true value, then follow the connector route “yes” and label the reason with “3.4 Yes Bad”. Again, this information is likely to be found in the Results section text or probably in the table. It can also be identified in the flowchart, but flowchart should be the last resort.

Even if the reported reasons for missingness are found, proceed to Flowchart 16, and determine if these reported reasons differ between the intervention groups. If the reported reasons are different across the intervention groups, mark them and label them as “3.4 Yes Bad”, and if the reported reasons across the groups are similar then mark them and label with “3.4 No Good”. Next, go to Flowchart 17 and search for text descriptions related to the outcome that suggest the true value could influence missingness. If such text is found, highlight it and label it as “3.4 Yes Bad”. If the text suggests that the true value could not influence missingness, mark it and label it as “3.4 No Good”. Regardless of the label choice, go to the next Flowchart 18. The flowchart asks whether any information about switching interventions that led to dropout in the study was found. Annotate the text related to missing outcomes, information about switching interventions, and reasons for missing outcomes or dropouts. If the missing outcome data due to switching interventions exceeds 5%, mark the description and follow the “yes” connector, labeling it as “3.4 Yes Bad”. If the missing outcome data due to switching interventions is less than 5%, mark the description and follow the "no" connector, labeling it as “3.4 No Good”.

## Annotation Guidelines for RoB Domain 4

The fourth risk domain in the RoB 2 tool is referred to as “Bias in the measurement of the outcome” for the outcome of interest. This domain aims to evaluate whether there are potential sources of bias in how the outcome is assessed, including issues such as the inadequate blinding of outcome assessors, the reliability and validity of outcome measurement methods, and inconsistent outcome reporting [35, 36].

## Signalling Question 4.1

The signalling question 4.1, “Was the method of outcome measurement inappropriate?” aims to evaluate the appropriateness, validity, and reliability the outcome measurement method employed to measure the outcomes of interest from the study. The validity of an outcome measurement method refers to its ability to effectively capture the construct being investigated, while reliability refers to the consistency and reproducibility of the measurement. Using an appropriate method of outcomes measurement ensures the accurate assessment of intervention effects. Outcomes measurement method should not only be appropriate but also reliable and valid for the chosen outcome as it impacts the interpretability and applicability of the trial results [37]. For example, the relevance of outcome measures to the specific patient population under study is essential for ensuring that the trial results are meaningful and applicable to the target population for which intervention is tested [38, 39, 37].

For annotation instructions for this signalling question, “RoB 4.1 Was the method of outcome measurement inappropriate?” follow the Flowchart in placards slide 26. As per the explanation above, the flowchart requests the annotators to identify the measurement method for the outcome of interest. If you find information about the validity of the measurement method, mark the sentences that discuss this along with the name of the measurement tool. Despite the validity being clarified, assess whether you consider this outcome measurement as appropriate and label the marked sentences as “4.1 Yes Bad” if you believe the method was inappropriate or as “4.1 No Good” if you believe it was appropriate. If there is no information about the validity of the measurement tool or method, mark the name of the outcome measurement and label it as “4.1 No Information”. This information is found in the Methods section of the RCTs.

## Signalling Question 4.2

The signalling question 4.2 “Could measurement or ascertainment of the outcome have differed between intervention groups?” assesses whether outcome measurement methods could have differed between the intervention groups in a study. This question examines whether the methods used for outcome measurement were valid, consistent, and applied equally to all intervention groups. For example, if one group is assessed using a more sensitive or specific measurement tool, it may lead to overestimation or underestimation of treatment effects compared to the other group leading to systematic biases. In general, unequal, or inconsistent outcome measurement methods between the groups can undermine the internal validity of the study [40].

For annotation instructions for this signalling question, “RoB 4.2 Could measurement or ascertainment of the outcome have differed between intervention groups?” follow the Flowchart in placards slide 27. Following the explanation above, the annotators are asked to find the outcome measurement methods for all intervention groups (or intervention and control). Mark the sentences describing outcome measurement methods from all intervention groups. If the annotator identifies disparity between the outcome measurement methods, then label the sentences with “4.2 Yes Bad”, and “4.2 No Good” otherwise. For example, “The control group's gait speed was measured using a standard stopwatch-based manual timing method. The intervention group's gait speed was measured using an advanced motion capture system. This significant differences in measurement methods raises concerns regarding potential biases and systematic differences in outcome assessment. In contrast, “Both the control group and the experimental group utilized a validated visual analog scale (VAS) to assess pain intensity”. The uniformity in the measurement methods increases the internal validity as well as comparability of the outcomes.

## Signalling Question 4.3

The signalling question 4.3 “Were outcome assessors aware of the intervention received by the study participants?” assesses the blinding or masking of outcome assessors in a study. If the outcome assessors are unblinded, they may unintentionally or inadvertently favor one intervention over another based on preconceived notions about the interventions. Blinding of outcome assessors is particularly important in studies where subjective outcomes are assessed which is the case in physiotherapy and rehabilitation. For instance, a systematic review and meta-analysis on acupuncture for the treatment of peripheral neuropathy highlighted the potential bias introduced if outcome assessors are not blinded to treatment assignment [41]. Originally, the impetus on blinding patients, administrators and outcome assessor came into clinical trials to reduce bias due expectations or preferences. Therefore, blinding the outcome assessors helps minimize the risk of bias and ensures that the assessments are conducted objectively and independently from knowledge of the intervention [42, 43].

For annotation instructions for this signalling question, “RoB 4.3 Were outcome assessors aware of the intervention received by the study participants?” follow the Flowchart in placards slide 28. Following the explanation above, the first diamond in the flowchart instructs the annotators to identify information about assessor blinding. If assessor blinding information is not found, it will be automatically marked as “2.3 No Information” for this signalling question, following the “No” arrow. If proper assessor blinding description is found or the sentences describing that the outcome assessors were unaware of the assigned intervention, then annotate it and mark the sentence with “4.3 No Good” label. In certain cases, outcome assessors are not blinded or could not be blinded, then mark such descriptions with label “4.3 Yes Bad”. For example, “The outcome assessors were aware of the intervention received by participants, as the study aimed to evaluate the impact of a behavioral intervention on self-reported anxiety levels.” This information if not found from the Methods sections, could also be annotated in the study abstract.

## Signalling Question 4.4

The signalling question 4.4 “Could assessment of the outcome have been influenced by knowledge of intervention received?” evaluates the potential for bias resulting from outcome assessors' awareness of the intervention received by the participants. This question aims to assess whether outcome assessments “could” have been influenced or biased by the assessors' knowledge of the intervention assignment. As explained in the signalling question 4.3, that assessor’s awareness of assigned intervention could cause biases [42, 43, 41].

For annotation instructions for this signalling question, “RoB 4.4 Could assessment of the outcome have been influenced by knowledge of intervention received?” follow the Flowchart in placards slide 29. The first diamond in the flowchart asks the annotators to look for descriptions of outcomes assessed. If the outcome of interest is subjective outcome, then annotate the sentence describing the outcome measurement in context of its assessment and label the description as “4.4 Yes Bad”. If the outcome of interest is objective outcome or mortality outcome, then annotate the sentence describing the outcome measurement in context of its assessment, but this time around label the description as “4.4 No Good”. For the domains of physiotherapy and rehabilitation, the question is quite theoretical and solely depends on whether the outcome assessed was subjective, objective or mortality.

## Signalling Question 4.5

The signalling question 4.5 “Is it likely that the assessment of the outcome was influenced by knowledge of the intervention received?” evaluates the likelihood that the outcome assessment was biased due to assessors' knowledge of the intervention received by the participants. The question is like the question 4.4, but it directly asks about the likelihood of the assessment being influenced by knowledge of the intervention. It seeks a determination of the probability or likelihood of such an influence being present [42, 43, 41].

For annotation instructions for this signalling question, “RoB 4.5 Is it likely that the assessment of the outcome was influenced by knowledge of the intervention received?” follow the Flowchart in placards slide 30. The first diamond in the flowchart asks the annotators to find text that hints if the outcome assessment was influenced by the knowledge of intervention used. If such a text is identified, then is it likely that the outcome assessment was influenced by the knowledge of the intervention received, if not the mark the identified description with the label “4.5 No Good”, and otherwise as “4.5 Yes Bad”. The information is likely found in the Methods section.

## Annotation Guidelines for RoB Domain 5

Risk domain 5 in RoB 2 refers to the “Bias in the selection of reported results”. This domain assesses the bias risk that may arise from the selective reporting of outcomes or analyses within a study. Selective reporting can lead to biased or incomplete reporting of results, which may distort the overall findings and affect the interpretation of the study's conclusions. The assessment of risk domain 5 helps determine whether the study has adequately reported all the intended outcomes and analyses as specified in the study protocol or pre-specified plans. This ensures transparency and reduces the risk of bias arising from selective reporting. Assessment of this domain requires trial protocol document in addition to the RCT itself [44].

## Signalling Question 5.1

The signalling question 5.1, “Were the data that produced this result analyzed in accordance with a pre-specified analysis plan that was finalized before unblinded outcome data were available for analysis?” evaluates whether results were analyzed according to a pre-defined analysis plan that was established before the researchers had access to the unblinded outcome data. If the analysis plan was established before accessing the unblinded outcome results and was followed correctly after obtaining the results; then it suggests that the analysis was conducted independently of the researcher's knowledge of the outcome, reducing the potential for bias in the interpretation of the results. If an analysis plan was established after accessing the results of the outcome, then some risk of bias could be introduced by knowledge of the results [45].

The annotation instructions for SQ 5.1 are provided in Flowchart in placards slide 32. The first diamond in the flowchart asks the annotators to check whether a trial protocol or registry or statistical analysis plan is to be found attached to the RCT. If a protocol is not found, then the study will be automatically considered as “5.1 No Information” for this signalling question. If a trial protocol is found, the annotator proceeds to the second diamond in the flowchart. The second diamond asks the annotator to find the outcome measure used to assess the target outcome in the trial protocol. If this information is not found, then the study will be automatically considered as “5.1 No Information”. If the outcome measure is found in the trial protocol, the flowchart directs annotators to compare it with the outcome measures for the target listed in the clinical trial. This information should be found in the methods section of the clinical trial. If found, is the outcome measure used to measure target outcome in the trial protocol the same as outcome measures as listed in the clinical trial? Annotate the sentences describing this information. If the outcome measures as listed in the protocol and the clinical study are same, then label the annotated information in both trial protocol and the trial as “5.1 Yes Good” and otherwise “5.1 No Bad”.

## Signalling Question 5.2

The signalling question 5.2, “Is the numerical result being assessed likely to have been selected, on the basis of the results, from multiple eligible outcome measurements (e.g., scales, definitions, time points) within the outcome domain?” assesses whether the trial authors selectively chose a specific outcome measurement from various possible outcome measurements such as choosing from different scales, definitions, or time points, based on the findings obtained. If a specific outcome measure was selectively chosen out of many, it could mean it was chosen as it yielded favorable result thereby introducing bias risk in the study. The reported result should be genuinely chosen based on a predetermined protocol plan and not selectively chosen based on the obtained results because it could introduce a bias in favour of a particular outcome measurement [46, 47].

For annotation instructions of this signalling question 5.2 follow the Flowchart in placards slide 33. The first diamond in the flowchart asks the annotators to check whether a trial protocol or registry or statistical analysis plan is to be found attached to the RCT. If a protocol is not found, then the study will be automatically considered as “5.2 No Information” for this signalling question. If a trial protocol is found, the annotator proceeds to the second diamond in the flowchart. If a trial protocol is found, follow to identify the outcome measures used to assess the target outcome in the trial protocol and annotate it. This information should be found in the trial protocol and if not found then the study will be automatically considered as “5.2 No Information”. If the information is found, the annotator moves to the third diamond in the flowchart which asks if some of these outcome measures found in the protocol omitted in the clinical trial? This information should be found in the methods section of the clinical trial and if found annotate it. If all the outcome measures as listed in the protocol are reported in the clinical study, then label the trial as “5.2 Yes Good” and otherwise “5.2 No Bad”.

## Signalling Question 5.3

This signalling question 5.3, “Is the numerical result being assessed likely to have been selected, based on the results, from multiple eligible analyses of the data?” aims to determine whether the researchers selectively chose a specific numerical result among various possible data analyses based on the findings they obtained. When researchers have flexibility in conducting multiple analyses on the same dataset and selecting and reporting the analysis that yields favorable results and supports some hypothesis, it could potentially introduce some risk of bias [48]. Hence this question aims to analyze whether a predefined analysis plan was in place in the trial protocol and whether it was genuinely followed for results analysis and reporting to ensure transparency and mitigate the risk of biased reporting.

For annotation instructions for the SQ 5.3 follow the Flowchart in placards slide 34. In accordance with the explanation above, the first diamond in the flowchart asks the annotators if a trial protocol is found. If a trial protocol is not found, then the study will be automatically considered as “5.3 No Information”. If a trial protocol is found, the annotators are asked to find all the prespecified methods of analysis used to assess effect of intervention on the target outcome in the trial protocol? Mark the sentence that mentions them. Check if all the outcomes analysis methods found in the trial protocol were also found in the RCT. If some of these methods of analysis found in the protocol were omitted in the clinical trial or in case there are additional analysis methods not reported in the protocol, then annotate the sentence(s) that mentions these methods in context of intervention effect assessment. If some of the methods were missing or some additional methods were conducted for outcomes analysis, then label these descriptions as “5.3 No Bad” and otherwise “5.3 Yes Good”. This information should be found in the methods section (statistical analysis) of the clinical trial.

## The Corpus Documents

Table 1 lists down all the RCTs in RoBuster along with the outcome being assessed for bias risk. The corpus comprised 41 RCTs with 17 subjective outcomes, 17 objective outcomes and 7 focusing on mortality outcomes, respectively. All these RCT PDFs have CC-BY-0 licenses [49].

Table 1: List of RCTs annotated in this project along with the type of target outcome assessed for risk of bias. ^a-c^

|  | Name | Outcome | Type |
| --- | --- | --- | --- |
| 1 | *Hassett 2020* [50] | Upright time | Objective |
| 2 | Mills 2019 [51] | Very low density lipo protein | Objective |
| 3 | *Wyke 2019* [52] | Steps per day | Objective |
| 4 | Cameron 2013 [53] | Frailty | Objective |
| 5 | *Taylor 2016* [54] | Chronic pain grade | Subjective |
| 6 | *Stuck 2015* [55] | All cause mortality | Mortality |
| 7 | Gallagher 2014 [56] | Mortality | Mortality |
| 8 | Tanaka 2020 [57] | Carotid atherosclerosis | Objective |
| 9 | Oyama 2016 [58] | Carotid Artery Intima-Media Thickness (IMT) 24 month | Objective |
| 10 | *Zhang 2020* [59] | Frequency HIV testing | Objective |
| 11 | Moholdt 2012 [60] | Peak oxygen uptake | Objective |
| 12 | Hardeman 2020 [61] | Activity volume | Objective |
| 13 | Garnaes 2016 [62] | Gestational diabetes mellitus | Objective |
| 14 | Gajados 2010 [63] | 8 hour without oxygen | Objective |
| 15 | Menant 2018 [64] | Dizziness handicap inventory | Subjective |
| 16 | Perlman 2012 [65] | WOMAC total 8 weeks | Subjective |
| 17 | Lawlor 2018 [66] | Alzheimer's Disease Assessment Scale-Cognitive Subscale 12 (ADAS Cog12) | Subjective |
| 18 | Barker 2019 [67] | Falls per person year | Subjective |
| 19 | +Bjerre 2019 [68] | Quality of life (QoL) | Subjective, Objective |
| 20 | *Myer 2018* [69] | Infant mortality | Mortality |
| 21 | *Lockman 2012* [70] | Death | Mortality |
| 22 | *Pasha 2013 [71] | Mean perinatal mortality | Mortality |
| 23 | *Darlow 2019 [72] | Roland Morris Disability Questionnaire 6 Months | Subjective |
| 24 | *Legro 2022 [73] | Healthy live birth | Objective |
| 25 | *Osteras 2019 [18] | Patient reporting quality of care 6 months | Subjective |
| 26 | *Friedli 2020 [74] | Mortality within 120 days | Mortality |
| 27 | *Becker 2020 [75] | Restriction muscle strength at 3rd Followup | Objective |
| 28 | Solomons 2020 [76] | Victorian Institute of Sports Assessment - Achilles (VISA A) | Subjective |
| 29 | Rossetti 2020 [77] | Risk Ratio Death 6 months | Mortality |
| 30 | Roman 2016 [78] | Timed Up and Go Test (TUG) | Objective |
| 31 | Raeissadat 2020 [79] | Visual analog scale (VAS) Pain 12 month | Subjective |
| 32 | Tagalidou 2019 [80] | Coping Humour Scale | Subjective |
| 33 | *Eskilsson 2017* [81] | Burnout T2 | Subjective |
| 34 | Ahmadi 2018 [82] | Craving Score Day 5 | Subjective |
| 35 | *Bai 2020 [83] | Generalized Anxiety Disorder 7-item (GAD 7) at 3 months | Subjective |
| 36 | +Bjerre 2019 [68] | Bone Mineral Density (BMD) | Objective |
| 37 | *Thorndike 2014* [84] | Mean steps per day | Objective |
| 38 | Liu 2018 [85] | Short Physical Performance Battery (SPPB) | Subjective |
| 39 | Rodrigo-Claverol 2019 [86] | WOMAC Post | Subjective |
| 40 | *Gleason 2015* [87] | Modified Mini-Mental State Examination (3ms) 48 months | Subjective |
| 41 | *An 2020 [88] | Peak oxygen uptake (VO2peak) at 6 months | Objective |

^a^ *Italics* = The documents marked in italics were the ones used for the manual LLM evaluation.

^b^ (Asterisk *) = The documents marked with Asterisk were the doubly annotated RCTs (n=9) used for inter-annotator agreement calculation.

^C^ +The study was assessed for two target outcomes- one objective and one subjective

## Conflict Resolution

Of 41 RCTs in RoBuster, inter-annotator agreement was calculated over nine doubly annotated documents. We conducted a face-to-face conflict resolution session to address every case of conflict that occurred in these doubly annotated RCTs. Our aim for conflict resolution between the annotators was to improve the instructions in the visual placards. For multiple conflicting cases, the instructions were either unclear, interpreted differently, or the annotators forgot to annotate. However, when the annotators went through the conflicts, they were able to resolve them. Three conflict groups were identified and resolved.

*Disparate regions conflict* was when both the annotators select different parts of text to answer the same SQ using the same response option. For example, in [72] to answer the SQ 2.2, one of the annotators selected the text “GPs were aware of group allocation (but not intervention content) when they completed their baseline measures” and another annotators selected the text “Practices and GPs were necessarily unblinded post randomization to facilitate planning and delivery of intervention workshops and related data collection.” and came to the same response option judgment of “Yes Bad”. Even though the annotators were restricted to annotating this SQ in the Methods section of the paper, they selected different parts of the text within the same section leading to this conflict. To resolve this conflict, we annotated both the parts of text as consensus annotation in the final version.

*Text span conflict* was when one annotator selected a part of text and another the same text but a longer span. For instance, in [76] to answer the SQ number 4.3, one annotator selected the complete sentence, “*The primary outcome for which the study was powered was the change from baseline in 12 week VISA-A (Victorian Institute of Sports Assessment–Achilles), a valid and reliable disease-specific outcome measure [37] which also includes an activity-related pain scale.*” while the other annotators only selected the text in CAPS. It was easier to resolve this conflict and we selected the longer annotation as the representative and used it as a consensus label. It's crucial to emphasize that while both text span conflicts and disparities in the region do not impact Cohen’s $\kappa$, they could result in a lower F1-measure score.

*Polarity conflict* was when the annotators marked same part of text to answer the same SQ in the same document being annotated but labelled them with polar opposite judgment options (“yes” vs. “No”). For instance, in [76] to answer the SQ 1.2, both the annotators select the text “Only AS and a research assistant not involved in participant intervention or assessments had access to the random allocation sequence.” but one labels the text with response judgement “No Bad” and another with “Yes Good”. Both annotators agreed that it was a badly formulated sentence in the RCT and agreed to judge the SQ with “Yes Good” giving the study authors a benefit of doubt.

## Prompts for LLM Evaluation

The list below enumerates the prompts used for LLM evaluation for all the signalling questions [1].

1. RoB 1.1: Was the allocation sequence random? Provide an answer and extract the supporting sentences that you write your answer based on. Extract the sentences in JSON.
2. RoB 1.2 Was the allocation sequence concealed until participants were enrolled and assigned to interventions? Provide an answer and extract the supporting sentences that you write your answer based on. Extract the sentences in JSON.
3. RoB 1.3 Did baseline differences between intervention groups suggest a problem with the randomization process? Provide an answer and extract the supporting sentences that you write your answer based on. Extract the sentences in JSON.
4. RoB 2.1: Were participants aware of their assigned intervention during the trial? Provide an answer and extract the supporting sentences that you write your answer based on. Extract the sentences in JSON.
5. RoB 2.2: Were carers and people delivering the interventions aware of participants' assigned intervention during the trial? Provide an answer and extract the supporting sentences that you write your answer based on. Extract the sentences in JSON.
6. RoB 2.3: Were there deviations from the intended intervention that arose because of the trial context? Provide an answer and extract the supporting sentences that you write your answer based on. Extract the sentences in JSON.
7. RoB 2.4 Were these deviations likely to have affected the outcome? Provide an answer and extract the supporting sentences that you write your answer based on. Extract the sentences in JSON.
8. RoB 2.5: Were these deviations from the intended intervention balanced between groups? Provide an answer and extract the supporting sentences that you write your answer based on. Extract the sentences in JSON.
9. RoB 2.6: Was an appropriate analysis used to estimate the effect of assignment to intervention? Provide an answer and extract the supporting sentences that you write your answer based on. Extract the sentences in JSON.
10. RoB 2.7 Was there potential for a substantial impact (on the result) of the failure to analyse participants in the group to which they were randomized? Provide an answer and extract the supporting sentences that you write your answer based on. Extract the sentences in JSON.
11. RoB 3.1 Were data for this outcome available for all, or nearly all, Participants randomized? Provide an answer and extract the supporting sentences that you write your answer based on. Extract the sentences in JSON.
12. RoB 3.2 Is there evidence that the result was not biased by missing outcome data? Provide an answer and extract the supporting sentences that you write your answer based on. Extract the sentences in JSON.
13. RoB 3.3 Could missingness in the outcome depend on its true value? Provide an answer and extract the supporting sentences that you write your answer based on. Extract the sentences in JSON.
14. RoB 3.4 Is it likely that missingness in the outcome depended on its true value? Provide an answer and extract the supporting sentences that you write your answer based on. Extract the sentences in JSON.
15. RoB 4.1 Was the method of measurement of the outcome inappropriate? Provide an answer and extract the supporting sentences that you write your answer based on. Extract the sentences in JSON.
16. RoB 4.2 Could measurement or ascertainment of the outcome have differed between intervention groups? Provide an answer and extract the supporting sentences that you write your answer based on. Extract the sentences in JSON.
17. RoB 4.3 Were outcome assessors aware of the intervention received by study participants? Provide an answer and extract the supporting sentences that you write your answer based on. Extract the sentences in JSON.
18. RoB 4.4 Could the assessment of the outcome have been influenced by knowledge of the intervention received? Provide an answer and extract the supporting sentences that you write your answer based on. Extract the sentences in JSON.
19. RoB 4.5 Is it likely that the assessment of the outcome was influenced by knowledge of the intervention received? Provide an answer and extract the supporting sentences that you write your answer based on. Extract the sentences in JSON.
20. RoB 5.1 Were the data that produced this result analyzed in accordance with a pre-specified analysis plan that was finalized before unblinded outcome data were available for analysis? Provide an answer and extract the supporting sentences that you write your answer based on. Extract the sentences in JSON.
21. RoB 5.2 Is the numerical result being assessed likely to have been selected, on the basis of the results, from multiple eligible outcome measurements (e.g., scales, definitions, time points) within the outcome domain? Provide an answer and extract the supporting sentences that you write your answer based on. Extract the sentences in JSON.
22. RoB 5.3 Is the numerical result being assessed likely to have been selected, on the basis of the results, from multiple eligible analyses of the data? Provide an answer and extract the supporting sentences that you write your answer based on. Extract the sentences in JSON.

# Bibliography

| [1] | J. A. Sterne, J. Savović, M. J. Page, R. G. Elbers, N. S. Blencoew, I. Boutron, C. J. Cates, H.-Y. Cheng, M. S. Corbett, S. M. Eldridge, J. R. Emberson, H. A. Miguel, H. Sally and Hróbjartsso, "RoB 2: a revised tool for assessing risk of bias in randomised trials," *BMJ,* vol. 366, 2019. |
| --- | --- |
| [2] | M. David , S. Hopewell, K. F. Schulz, V. Montori , P. C. Gøtzsche, P. J. Devereaux, D. Elbourne, M. Egger and D. G. Altman, "CONSORT 2010 Explanation and Elaboration: updated guidelines for reporting parallel group randomised trials," *BMJ,* vol. 340, p. c869, 2010. |
| [3] | S. Bello, "Contemporary methodological issues in drug development: the functionality of blinding is poorly studied," *Journal of Evidence-Based Medicine,* vol. 10, no. 1, pp. 61--65, 2017. |
| [4] | H. Saltaji, S. Armijo-Olivo, G. G. Cummings, M. Amin and C. Flores-Mir, "Randomized clinical trials in dentistry: Risks of bias, risks of random errors, reporting quality, and methodologic quality over the years 1955--2013," *PloS one,* vol. 12, no. 12, p. e0190089, 2017. |
| [5] | A. F. A. Rahim, M. N. Norhayati and A. M. Zainudin, "The effect of a brown-rice diets on glycemic control and metabolic parameters in prediabetes and type 2 diabetes mellitus: a meta-analysis of randomized controlled trials and controlled clinical trials," *PeerJ,* vol. 9, p. e11291, 2021. |
| [6] | C. Roberts and D. J. Torgerson, "Baseline imbalance in randomised controlled trials," *BMJ,* vol. 319, no. 7203, p. 185, 1999. |
| [7] | S. P. Cohen, R. W. Hurley, C. C. Buckenmaier, C. Kurihara, B. Morlando and A. Dragovich, "Randomized placebo-controlled study evaluating lateral branch radiofrequency denervation for sacroiliac joint pain," *The Journal of the American Society of Anesthesiologists,* vol. 109, no. 2, pp. 279--288, 2008. |
| [8] | I. Abraha, A. Cherubini, F. Cozzolino, R. De Florio, M. L. Luchetta, J. M. Rimland, I. Folletti, M. Marchesi, A. Germani, M. Orso, P. Eusebi and A. Montedori, "Deviation from intention to treat analysis in randomised trials and treatment effect estimates: meta-epidemiological study," *BMJ,* vol. 350, 2015. |
| [9] | Z. E. Imel, K. Laska, M. Jakupcak and T. L. Simpson, "Meta-analysis of dropout in treatments for posttraumatic stress disorder.," *Journal of consulting and clinical psychology,* vol. 81, no. 3, p. 394, 2013. |
| [10] | N. R. Cedenilla, J. I. C. Arenillas, S. A. Valero and A. S. Guzmán, "Psychosocial Interventions for the Treatment of Cancer-Related Fatigue: An Umbrella Review," *Current Oncology,* vol. 30, no. 3, pp. 2954--2977, 2023. |
| [11] | G. E. Swan, L. M. Jack, S. Curry, M. Chorost, H. Javitz, T. McAfee and S. Dacey, "Bupropion SR and counseling for smoking cessation in actual practice: Predictors of outcome," *Nicotine & Tobacco Research,* vol. 5, no. 6, pp. 911--921, 2003. |
| [12] | N. E. O'Connell, J. Cossar, L. Marston, B. M. Wand, D. Bunce, G. L. Moseley and L. H. De Souza, "Rethinking clinical trials of transcranial direct current stimulation: participant and assessor blinding is inadequate at intensities of 2mA," vol. 7, no. 10, p. e47514, 2012. |
| [13] | S. Andersson-Marforio, A. Lundkvist Josenby, E. Ekvall Hansson and C. Hansen, "The effect of physiotherapy including frequent changes of body position and stimulation to physical activity for infants hospitalised with acute airway infections. Study protocol for a randomised controlled trial," *Trials,* vol. 21, pp. 1--10, 2020. |
| [14] | S. C. Buttery, W. Banya, R. Bilancia, E. Boyd, J. Buckley, N. J. Greening, K. Housley, S. Jordan, S. V. Kemp, A. J. Kirk, L. Latimer, K. Lau, R. Lawson, A. Lewis, J. Moxham, S. Rathinam and Steine, "Lung volume reduction surgery versus endobronchial valves: a randomised controlled trial," *European Respiratory Journal,* vol. 61, no. 4, 2023. |
| [15] | A. Hróbjartsson and I. Boutron, "Blinding in randomized clinical trials: imposed impartiality," *Clinical Pharmacology & Therapeutics,* vol. 90, no. 5, pp. 732--736, 2011. |
| [16] | N. D. Chiaravalloti, N. B. Moore and J. DeLuca, "The efficacy of the modified Story Memory Technique in progressive MS," *Multiple Sclerosis Journal,* vol. 26, no. 3, pp. 354--362, 2020. |
| [17] | C. Paterson, T. Karatzias, S. Harper, N. Dougall, A. Dickson and P. Hutton, "A feasibility study of a cross-diagnostic, CBT-based psychological intervention for acute mental health inpatients: Results, challenges, and methodological implications," *British Journal of Clinical Psychology,* vol. 58, no. 2, pp. 211--230, 2019. |
| [18] | N. Østerås, T. Moseng, L. van Bodegom-Vos, K. Dziedzic, I. Mdala, B. Natvig, J. H. Røtterud, U.-B. Schjervheim, T. V. Vlieland, Ø. Andreassen, J. N. Hansen and K. B. Hagen, "Implementing a structured model for osteoarthritis care in primary healthcare: a stepped-wedge cluster-randomised trial," *PLoS medicine,* vol. 16, no. 10, p. e1002949, 2019. |
| [19] | M. O. Folayan, M. O. Alade and E. O. Oziegbe, "Challenges with study procedure fidelity when conducting household survey: reports from the field," *BMC Research Notes,* vol. 12, pp. 1--5, 2019. |
| [20] | M. Merli, M. Merli, G. Mariotti, U. Pagliaro, M. Moscatelli and M. Nieri, "Immediate versus early non-occlusal loading of dental implants placed flapless in partially edentulous patients: A 10-year randomized clinical trial," *Journal of Clinical Periodontology,* vol. 47, no. 5, pp. 621--629, 2020. |
| [21] | S. Dodd, I. R. White and P. Williamson, "Nonadherence to treatment protocol in published randomised controlled trials: a review," *Trials,* vol. 13, no. 2012, pp. 1--16. |
| [22] | Y. Rozenfeld and J. S. Hunt, "Effect of patient withdrawal on a study evaluating pharmacist management of hypertension," *Pharmacotherapy: The Journal of Human Pharmacology and Drug Therapy,* vol. 26, no. 11, pp. 1565--1571, 2006. |
| [23] | B. M. Tomazini, I. S. Maia, A. B. Cavalcanti, O. Berwanger, R. G. Rosa, V. C. Veiga, A. Avezum, R. D. Lopes, F. R. Bueno, M. V. A. Silva and O. , "Effect of dexamethasone on days alive and ventilator-free in patients with moderate or severe acute respiratory distress syndrome and COVID-19: the CoDEX randomized clinical trial," *JAMA,* vol. 324, no. 13, pp. 1307--1316, 2020. |
| [24] | A. Prodeus, V. Niborski, J. Schrezenmeir, A. Gorelov, A. Shcherbina and A. Rumyantsev, "Fermented milk consumption and common infections in children attending day-care centers: a randomized trial," *Journal of pediatric gastroenterology and nutrition,* vol. 63, no. 5, p. 534. |
| [25] | S. K. Gupta, "Intention-to-treat concept: a review," *Perspectives in clinical research,* vol. 2, no. 3, p. 109, 2011. |
| [26] | C. Lewis, N. P. Roberts, S. Gibson and J. I. Bisson, "Dropout from psychological therapies for post-traumatic stress disorder (PTSD) in adults: Systematic review and meta-analysis," *European Journal of Psychotraumatology,* vol. 11, no. 1, p. 1709709, 2020. |
| [27] | C. L. Kaufmann, H. C. Schulberg and N. R. Schooler, "Chapter 14: Self-help group participation among people with severe mental illness," *Prevention in Human Services,* vol. 11, no. 2, pp. 315--331, 1995. |
| [28] | J. Gnang, Y. Kim, Y. Ren, J. Travis and Y. Kim, "An Empirical Comparison of Statistical Methods for Missing Data in Randomized, Double-Blind, Placebo-Controlled, Phase 3 Clinical Trials for Chronic Pain and Lipid-Lowering Products," *Therapeutic Innovation & Regulatory Science,* vol. 54, pp. 1416--1427, 2020. |
| [29] | N. Shara, S. Yassin, E. Valaitis, H. Wang, B. V. Howard, W. Wang, E. T. Lee and J. G. Umans, "Randomly and non-randomly missing renal function data in the strong heart study: a comparison of imputation methods," *PloS one,* vol. 10, no. 9, p. e0138923, 2015. |
| [30] | E. A. Akl, M. Briel, J. J. You, SunqXin, J. C. Bradley, J. W. Busse, S. Mulla, F. Lamontagne, D. Bassler, C. Vera, A. Mohamad, C. M. Katsios, Q. Zhou, T. Cukierman-Yaffe, A. Gangji and E. Mills, "Potential impact on estimated treatment effects of information lost to follow-up in randomised controlled trials (LOST-IT): systematic review," *BMJ,* vol. 344, 2012. |
| [31] | M. L. Bell, M. Fiero, N. J. Horton and C.-H. Hsu, "Handling missing data in RCTs; a review of the top medical journals," *BMC medical research methodology,* vol. 14, no. 1, pp. 1--8, 2014. |
| [32] | N. A. Khan, K. D. Torralba and F. Aslam, "Missing data in randomised controlled trials of rheumatoid arthritis drug therapy are substantial and handled inappropriately," *RMD open,* vol. 7, no. 2, p. e001708, 2021. |
| [33] | S. B. Goldberg, D. M. Bolt and R. J. Davidson, "Data missing not at random in mobile health research: Assessment of the problem and a case for sensitivity analyses," *Journal of medical Internet research,* vol. 23, no. 6, p. e26749, 2021. |
| [34] | M. Calvert, J. Blazeby, D. G. Altman, D. A. Revicki, D. Moher, M. D. Brundage and C. P. G. , "Reporting of patient-reported outcomes in randomized trials: the CONSORT PRO extension," *JAMA,* vol. 309, no. 8, pp. 814--822, 2013. |
| [35] | C. Heneghan, B. Goldacre and K. R. Mahtani, "Why clinical trial outcomes fail to translate into benefits for patients," *Trials,* vol. 18, no. 1, pp. 1--7, 2017. |
| [36] | R. Dadouch, M. Faheim, O. Susini, S. Sedra, M. Showell, R. D'Souza and C. i. , "Variation in outcome reporting in studies on obesity in pregnancy—A systematic review," *Clinical Obesity,* vol. 9, no. 6, p. e12341, 2019. |
| [37] | T. Rubin, J. Clayton, D. Adams, H. Jou and S. Vohra, "Systematic review of outcome measures in trials of pediatric anaphylaxis treatment," *BMC pediatrics,* vol. 14, no. 1, pp. 1--8, 2014. |
| [38] | P. R. Williamson, D. G. Altman, J. M. Blazeby, M. Clarke, D. Devane, E. Gargon and P. Tugwell, "Developing core outcome sets for clinical trials: issues to consider," *Trials,* vol. 13, pp. 1--8, 2012. |
| [39] | J. D. Grill, M. M. Nuño, D. L. Gillen and A. D. N. I. , "Which MCI patients should be included in prodromal Alzheimer’s disease clinical trials?," *Alzheimer disease and associated disorders,* vol. 33, no. 2, p. 104, 2019. |
| [40] | H. P. K. Enwald and M.-L. A. Huotari, "Preventing the obesity epidemic by second generation tailored health communication: an interdisciplinary review," *Journal of medical Internet research,* vol. 12, no. 2, p. e24, 2010. |
| [41] | A. Dimitrova, C. Murchison and B. Oken, "Acupuncture for the treatment of peripheral neuropathy: a systematic review and meta-analysis," *The Journal of Alternative and Complementary Medicine,* vol. 23, no. 3, pp. 164--179, 2017. |
| [42] | J. H. Watanabe, G. E. Simon, M. Horberg, R. Platt, A. Hernandez and R. M. Califf, "When are treatment blinding and treatment standardization necessary in real-world clinical trials?," *Clinical Pharmacology & Therapeutics,* vol. 111, no. 1, pp. 116--121, 2022. |
| [43] | A. Hróbjartsson, A. S. S. Thomsen, F. Emanuelsson, B. Tendal, J. Hilden, I. Boutron, P. Ravaud and S. Brorson, "Observer bias in randomized clinical trials with measurement scale outcomes: a systematic review of trials with both blinded and nonblinded assessors," *CMAJ,* vol. 185, no. 4, pp. E201--E211, 2013. |
| [44] | K. Dwan, C. Gamble, P. R. Williamson, J. J. Kirkham and R. B. G. , "Systematic review of the empirical evidence of study publication bias and outcome reporting bias—an updated review," *PloS one,* vol. 8, no. 7, p. e66844, 2013. |
| [45] | A. Riiser, E. Bere, L. B. Andersen and S. Nordengen, "E-cycling and health benefits: A systematic literature review with meta-analyses," *Frontiers in sports and active living,* vol. 4, p. 1031004, 2022. |
| [46] | J. Rankin, A. Ross, J. Baker, M. O'Brien, C. Scheckel and M. Vassar, "Selective outcome reporting in obesity clinical trials: a cross-sectional review," *Clinical obesity,* vol. 7, no. 4, pp. 245--254, 2017. |
| [47] | L. Bouter, L. Horn and S. Kleinert, "Research integrity and societal trust in research," *SA Heart,* vol. 18, no. 2, pp. 80--81, 2021. |
| [48] | M. J. Swanson, J. L. Johnston and J. S. Ross, "Registration, publication, and outcome reporting among pivotal clinical trials that supported FDA approval of high-risk cardiovascular devices before and after FDAAA," *Trials,* vol. 22, no. 1, p. 817, 2021. |
| [49] | "Creative Commons CC0," [Online]. Available: https://creativecommons.org/public-domain/cc0/).. [Accessed April 2023]. |
| [50] | L. Hassett, M. van den Berg, R. I. Lindley, M. Crotty, A. McCluskey, H. P. van der Ploeg, S. T. Smith, K. Schurr, H. Kirsten, M. L. Hackett and O. , "Digitally enabled aged care and neurological rehabilitation to enhance outcomes with Activity and MObility UsiNg Technology (AMOUNT) in Australia: A randomised controlled trial," *PLoS medicine,* vol. 17, no. 2, p. e1003029, 2020. |
| [51] | H. L. Mills, N. Patel, S. L. White, D. Pasupathy, A. L. Briley, D. L. Santos Ferreira, P. T. Seed, S. M. Nelson, N. Sattar, K. Tilling and O. , "The effect of a lifestyle intervention in obese pregnant women on gestational metabolic profiles: findings from the UK Pregnancies Better Eating and Activity Trial (UPBEAT) randomised controlled trial}," *BMC medicine,* vol. 17, pp. 1--12, 2019. |
| [52] | S. Wyke, C. Bunn, E. Andersen, M. N. Silva, F. Van Nassau, P. McSkimming, S. Kolovos, J. M. Gill, K. Hunt and O. , "The effect of a programme to improve men’s sedentary time and physical activity: The European Fans in Training (EuroFIT) randomised controlled trial," *PLoS medicine,* vol. 16, no. 2, p. e1002736, 2019. |
| [53] | I. D. Cameron, N. Fairhall, C. Langron, K. Lockwood, N. Monaghan, C. Aggar, C. Sherrington, S. R. Lord and S. E. Kurrle, "A multifactorial interdisciplinary intervention reduces frailty in older people: randomized trial," *BMC medicine,* vol. 11, no. 1, pp. 1--10, 2013. |
| [54] | S. J. Taylor, D. Carnes, K. Homer, B. C. Kahan, N. Hounsome, S. Eldridge, A. Spencer, T. Pincus, A. Rahman and M. Underwood, "Novel three-day, community-based, nonpharmacological group intervention for chronic musculoskeletal pain (COPERS): a randomised clinical trial," *PLoS medicine,* vol. 13, no. 6, p. e1002040, 2016. |
| [55] | A. E. Stuck, A. Moser, U. Morf, U. Wirz, J. Wyser, G. Gillmann, S. Born, M. Zwahlen, S. Iliffe, D. Harari, C. Swift, J. Beck and M. Egger, "Effect of health risk assessment and counselling on health behaviour and survival in older people: a pragmatic randomised trial," *PLoS medicine,* vol. 12, no. 10, p. e1001889, 2015. |
| [56] | G. Martin , C. Alan, B. Rinaldo, F. Simon, G. David, L. Joanne, L. Seringne, M. Shay, M. John, P. Rachael and . D. Rajbhandari, "Long-term survival and dialysis dependency following acute kidney injury in intensive care: extended follow-up of a randomized controlled trial," *PLoS medicine,* vol. 11, no. 2, p. e1001601, 2014. |
| [57] | A. Tanaka, I. Taguchi, H. Teragawa, N. Ishizaka, Y. Kanzaki, H. Tomiyama, M. Sata, A. Sezai, K. Eguchi, T. Kato, S. Toyoda, R. Ishibashi, K. Kario, T. Ishizu and S. Ueda, "Febuxostat does not delay progression of carotid atherosclerosis in patients with asymptomatic hyperuricemia: a randomized, controlled trial," *PLoS medicine,* vol. 17, no. 4, p. e1003095, 2020. |
| [58] | J.-i. Oyama, T. Murohara, M. Kitakaze, T. Ishizu, Y. Sato, K. Kitagawa, H. Kamiya, M. Ajioka, M. Ishihara, K. Dai, M. Nanasato, M. Sata, K. Maemura and H. Tomiyama, "The effect of sitagliptin on carotid artery atherosclerosis in type 2 diabetes: the PROLOGUE randomized controlled trial," *Plos medicine,* vol. 13, no. 6, p. e1002051, 2016. |
| [59] | C. Zhang, D. Koniak-Griffin, H.-Z. Qian, L. A. Goldsamt, H. Wang, M.-L. Brecht and X. Li, "Impact of providing free HIV self-testing kits on frequency of testing among men who have sex with men and their sexual partners in China: A randomized controlled trial," *PLoS medicine,* vol. 17, no. 10, p. e1003365, 2020. |
| [60] | T. Moholdt, M. B. Vold, J. Grimsmo, S. A. Slørdahl and U. Wisløff, "Home-based aerobic interval training improves peak oxygen uptake equal to residential cardiac rehabilitation: a randomized, controlled trial," *PloS one,* vol. 7, no. 7, p. e41199, 2012. |
| [61] | W. Hardeman, J. Mitchell, S. Pears, M. Van Emmenis, F. Theil, V. S. Gc, J. C. Vasconcelos, K. Westgate, S. Brage, M. Suhrcke, M. Suhrcke, S. J. Griffin, A. L. Kinmonth and E. Wilson, "Evaluation of a very brief pedometer-based physical activity intervention delivered in NHS Health Checks in England: The VBI randomised controlled trial," *PLoS medicine,* vol. 17, no. 3, p. e1003046, 2020. |
| [62] | K. K. Garnæs, S. Mørkved, Ø. Salvesen and T. Moholdt, "Exercise training and weight gain in obese pregnant women: a randomized controlled trial (ETIP trial)," *PLoS medicine,* vol. 13, no. 7, p. e1002079, 2016. |
| [63] | V. Gajdos, S. Katsahian, N. Beydon, V. Abadie, L. de Pontual , S. Larrar, R. Epaud, B. Chevallier, S. Bailleux, A. Mollet-Boudjemline , J. Bouyer, S. Chevret and P. Labrune, "Effectiveness of chest physiotherapy in infants hospitalized with acute bronchiolitis: a multicenter, randomized, controlled trial," *PLoS medicine,* vol. 7, no. 9, p. e1000345, 2010. |
| [64] | J. C. Menant, A. A. Migliaccio, D. . L. Sturnieks, C. Hicks, J. Lo, M. Ratanapongleka, J. Turner, K. Delbaere, N. Titov, D. Meinrath, C. McVeigh, J. C. T. Close and S. R. Lord, "Reducing the burden of dizziness in middle-aged and older people: a multifactorial, tailored, single-blind randomized controlled trial," *PLoS Medicine,* vol. 15, no. 7, p. e1002620, 2018. |
| [65] | A. I. Perlman, A. Ali, V. Y. Njike, D. Hom , A. Davidi, S. Gould-Fogerite, C. Milak and D. L. Katz, "Massage therapy for osteoarthritis of the knee: a randomized dose-finding trial," *PLoS One,* vol. 7, no. 2, p. e30248, 2012. |
| [66] | B. Lawlor, R. Segurado, S. Kennelly, M. G. M. O. Rikkert, R. Howard, F. Pasquier, A. Börjesson-Hanson, M. Tsolaki, U. Lucca, D. W. Molloy, R. Coen, M. W. Riepe and J. Kálmán, "Nilvadipine in mild to moderate Alzheimer disease: a randomised controlled trial," *PLoS medicine,* vol. 15, no. 9, p. e1002660, 2018. |
| [67] | A. Barker, P. Cameron, L. Flicker, G. Arendts, C. Brand, C. Etherton-Beer, A. Forbes, T. Haines, A.-M. Hill, P. Hunter, J. Lowthian, S. R. Nyman, J. Redfern, D. V. Smit and Wa, "Evaluation of RESPOND, a patient-centred program to prevent falls in older people presenting to the emergency department with a fall: A randomised controlled trial," *PLoS medicine,* vol. 16, no. 5, p. e1002807, 2019. |
| [68] | E. D. Bjerre, T. H. Petersen, A. B. Jørgensen, C. Johansen, P. Krustrup, B. Langdahl, M. H. Poulsen, S. S. Madsen, P. B. Østergren, M. Borre, M. Rørth, K. Brasso and Mid, "Community-based football in men with prostate cancer: 1-year follow-up on a pragmatic, multicentre randomised controlled trial," *PLoS Medicine,* vol. 16, no. 10, p. e1002936, 2019. |
| [69] | L. Myer, T. K. Phillips, A. Zerbe, K. Brittain, M. Lesosky, N.-Y. Hsiao, R. H. Remien, C. A. Mellins, J. A. McIntyre and E. J. Abrams, "Integration of postpartum healthcare services for HIV-infected women and their infants in South Africa: a randomised controlled trial," *PLoS medicine,* vol. 15, no. 3, p. e1002547, 2018. |
| [70] | S. Lockman, M. Hughes, F. Sawe, Y. Zheng, J. McIntyre, T. Chipato, A. Asmelash, M. Rassool, S. Kimaiyo, D. Shaffer, M. Hosseinipour, L. Mohapi, F. Ssali, M. Chibowa and F. Amod, "Nevirapine-versus lopinavir/ritonavir-based initial therapy for HIV-1 infection among women in Africa: a randomized trial," *PLoS medicine,* vol. 9, no. 6, p. e1001236, 2012. |
| [71] | O. Pasha, E. M. McClure, L. L. Wright, S. Saleem, S. S. Goudar, E. Chomba, A. Patel, F. Esamai, A. Garces, F. Althabe, B. Kodkany, H. Mabeya, A. Manasyan and W. A. Carlo, "A combined community-and facility-based approach to improve pregnancy outcomes in low-resource settings: a Global Network cluster randomized trial," *BMC medicine,* vol. 11, no. 1, pp. 1--12, 2013. |
| [72] | B. Darlow, J. Stanley, S. Dean, J. H. Abbott, S. Garrett, R. Wilson, F. Mathieson and A. Dowell, "The Fear Reduction Exercised Early (FREE) approach to management of low back pain in general practice: a pragmatic cluster-randomised controlled trial," *PLoS medicine,* vol. 16, no. 9, p. e1002897, 2019. |
| [73] | R. S. Legro, K. R. Hansen, M. P. Diamond, A. Z. Steiner, C. Coutifaris, M. I. Cedars, K. M. Hoeger, R. Usadi, E. B. Johnstone, D. J. Haisenleder, R. A. Wild, K. T. Barnhart, J. Mersereau and Trussel, "Effects of preconception lifestyle intervention in infertile women with obesity: The FIT-PLESE randomized controlled trial," *PLoS Medicine,* vol. 19, no. 1, p. e1003883, 2022. |
| [74] | N. Friedli, J. Baumann, R. Hummel, M. Kloter, J. Odermatt, R. Fehr, S. Felder, V. Baechli, M. Geiser, M. Deiss and O. , "Refeeding syndrome is associated with increased mortality in malnourished medical inpatients: secondary analysis of a randomized trial". |
| [75] | A. Becker, P. Angerer, J. Weber and A. Müller, "The prevention of musculoskeletal complaints: long-term effect of a work-related psychosocial coaching intervention compared to physiotherapy alone—a randomized controlled trial," *International archives of occupational and environmental health,* vol. 93, pp. 877--889, 2020. |
| [76] | L. Solomons, J. J. Lee, M. Bruce, L. D. White and A. Scott, "Intramuscular stimulation vs sham needling for the treatment of chronic midportion Achilles tendinopathy: a randomized controlled clinical trial," *PLoS One,* vol. 15, no. 9, p. e0238579, 2020. |
| [77] | A. O. Rossetti, K. Schindler, R. Sutter, S. Rüegg, F. Zubler, J. Novy, M. Oddo, L. Warpelin-Decrausaz and V. Alvarez, "Continuous vs routine electroencephalogram in critically ill adults with altered consciousness and no recent seizure: a multicenter randomized clinical trial," *JAMA neurology,* vol. 77, no. 10, pp. 1225--1232, 2020. |
| [78] | E. Román, C. García-Galcerán , T. Torrades, S. Herrera, A. Marín, M. Doñate, E. Alvarado-Tapias , J. Malouf, L. Nácher, R. Serra-Grima , C. Guarner, J. Cordoba and G. Soriano, "Effects of an exercise programme on functional capacity, body composition and risk of falls in patients with cirrhosis: a randomized clinical trial," *PLoS One,* vol. 11, no. 3, p. e0151652, 2016. |
| [79] | S. A. Raeissadat , A. Gharooee Ahangar , S. M. Rayegani, M. M. Sajjadi, A. Ebrahimpour and P. Yavari, "Platelet-rich plasma-derived growth factor vs hyaluronic acid injection in the individuals with knee osteoarthritis: a one year randomized clinical trial," *Journal of Pain Research,* pp. 1699--1711, 2020. |
| [80] | N. Tagalidou, E. Distlberger, V. Loderer and A.-R. Laireiter, "Efficacy and feasibility of a humor training for people suffering from depression, anxiety, and adjustment disorder: a randomized controlled trial," *BMC psychiatry,* vol. 19, no. 1, pp. 1--13, 2019. |
| [81] | T. Eskilsson, L. S. Järvholm, H. M. Gavelin, A. S. Neely and C.-J. Boraxbekk, "Aerobic training for improved memory in patients with stress-related exhaustion: a randomized controlled trial," *BMC psychiatry,* vol. 17, no. 1, pp. 1--10, 2017. |
| [82] | J. Ahmadi, M. S. Jahromi, D. Ghahremani and E. D. London, "Single high-dose buprenorphine for opioid craving during withdrawal," *Trials,* vol. 19, no. 1, pp. 1--7, 2018. |
| [83] | Y. Bai, X. Wu, R. C. Tsang, R. Yun, Y. Lu, E. Dean and A. Y. Jones, "A Randomised Controlled Trial to Evaluate the Administration of the Health Improvement Card as a Health Promotion Tool: A Physiotherapist-Led Community-Based Initiative," *International Journal of Environmental Research and Public Health,* vol. 17, no. 21, p. 8065, 2020. |
| [84] | A. N. Thorndike, S. Mills, L. Sonnenberg, D. Palakshappa, T. Deepak, C. T. Pau and S. Regan, "Activity monitor intervention to promote physical activity of physicians-in-training: randomized controlled trial," *PloS one,* vol. 9, no. 6, p. e100251, 2014. |
| [85] | Z. Liu, F.-C. Hsu, A. Trombetti, A. C. King, C. K. Liu, T. M. Manini, R. A. Fielding, M. Pahor, A. B. Newman, S. Kritchevsky and O. , "Effect of 24-month physical activity on cognitive frailty and the role of inflammation: the LIFE randomized clinical trial," *BMC medicine,* vol. 16, pp. 1--10, 2018. |
| [86] | M. Rodrigo-Claverol, C. Casanova-Gonzalvo, B. Malla-Clua, E. Rodrigo-Claverol , J. Jové-Naval and M. Ortega-Bravo , "Animal-assisted intervention improves pain perception in polymedicated geriatric patients with chronic joint pain: A clinical trial," *International journal of environmental research and public health,* vol. 16, no. 16, p. 2843, 2019. |
| [87] | C. E. Gleason, N. M. Dowling, W. Wharton, J. E. Manson, V. M. Miller, C. S. Atwood, E. A. Brinton, M. I. Cedars, R. A. Lobo, G. R. Merriam and O. , "Effects of hormone therapy on cognition and mood in recently postmenopausal women: findings from the randomized, controlled KEEPS--cognitive and affective study," *PLoS medicine,* vol. 12, no. 6, p. e1001833, 2015. |
| [88] | K.-Y. An, , D.-W. Kang, A. R. Morielli, C. M. Friedenreich, R. D. Reid, D. C. McKenzie, K. Gelmon, J. R. Mackey and K. S. Courneya, "Patterns and predictors of exercise behavior during 24 months of follow-up after a supervised exercise program during breast cancer chemotherapy," *International Journal of Behavioral Nutrition and Physical Activity,* vol. 17, no. 1, pp. 1--11, 2020. |

1. Outcome of interest is the target outcome assessed for bias risk. [↑](#footnote-ref-1)
