# Supplementary material for: RoBuster—Corpus Annotated With Risk of Bias Text Spans in Randomized Controlled Trials in Physiotherapy and Rehabilitation: Corpus Development and Annotation Study
Source: JMIR Form Res. 2026 Apr 27;10:e55127. doi: 10.2196/55127 (PMC13120535; doi:10.2196/55127)
Supplement: Multimedia Appendix 2 [file formative-v10-e55127-s002.pdf]

# *Difficulty: RoB assessment (Annotator 1)*

This section measures the **difficulty, subjectivity, and availability of information for assessing a RoB question** (RoB 1.1, RoB 1.2, ... RoB 5.3) in general. Select one of the five checkboxes based on the difficulty of that RoB question in the manual assessment. On a scale from 1-5, 1 is the easiest to assess, and 5 is the toughest to assess.

## *Risk Domain 1*

### *RoB 1.1 Was the allocation sequence random?*

| **Ease scale** | **Subjectivity scale** | **Availability of information** |
|---|---|---|
| 1 ☐ Very easy | 1 ☐ Very low | 1 ☐ Very good |
| 2 ☒ Easy | 2 ☒ Low | 2 ☒ Good |
| 3 ☐ Normal | 3 ☐ Normal | 3 ☐ Normal |
| 4 ☐ Difficult | 4 ☐ High | 4 ☐ Bad |
| 5 ☐ Very difficult | 5 ☐ Very High | 5 ☐ Very bad |

### RoB 1.2 *Was the allocation sequence concealed until participants were enrolled and assigned to interventions?*

| **Ease scale** | **Subjectivity scale** | **Availability of information** |
|---|---|---|
| 1 ☐ Very easy | 1 ☐ Very low | 1 ☐ Very good |
| 2 ☒ Easy | 2 ☒ Low | 2 ☒ Good |
| 3 ☐ Normal | 3 ☐ Normal | 3 ☐ Normal |
| 4 ☐ Difficult | 4 ☐ High | 4 ☐ Bad |
| 5 ☐ Very difficult | 5 ☐ Very difficult | 5 ☐ Very bad |

### RoB 1.3 *Did baseline differences between intervention groups suggest a problem with the randomization process?*

| **Ease scale** | **Subjectivity scale** | **Availability of information** |
|---|---|---|
| 1 ☐ Very easy | 1 ☐ Very low | 1 ☐ Very good |
| 2 ☒ Easy | 2 ☐ Low | 2 ☐ Good |
| 3 ☐ Normal | 3 ☒ Normal | 3 ☒ Normal |
| 4 ☐ Difficult | 4 ☐ High | 4 ☐ Bad |
| 5 ☐ Very difficult | 5 ☐ Very difficult | 5 ☐ Very bad |

## *Risk Domain 2*

RoB 2.1: *Were* ***participants*** *aware of their assigned intervention during the trial?*

**Ease scale**

1 ☐ Very easy
2 ☐ Easy
3 ☒ Normal
4 ☐ Difficult
5 ☐ Very difficult

**Subjectivity scale**

1 ☐ Very low
2 ☐ Low
3 ☒ Normal
4 ☐ High
5 ☐ Very difficult

**Availability of information**

1 ☐ Very good
2 ☐ Good
3 ☒ Normal
4 ☐ Bad
5 ☐ Very bad

RoB 2.2: *Were carers and people delivering the interventions aware of participants' assigned intervention during the trial?*

**Ease scale**

1 ☐ Very easy
2 ☐ Easy
3 ☒ Normal
4 ☐ Difficult
5 ☐ Very difficult

**Subjectivity scale**

1 ☐ Very low
2 ☐ Low
3 ☒ Normal
4 ☐ High
5 ☐ Very difficult

**Availability of information**

1 ☐ Very good
2 ☐ Good
3 ☒ Normal
4 ☐ Bad
5 ☐ Very bad

*RoB 2.3: Were there deviations from the intended intervention that arose because of the trial context?*

**Ease scale**

1 ☐ Very easy
2 ☐ Easy
3 ☐ Normal
4 ☒ Difficult
5 ☐ Very difficult

**Subjectivity scale**

1 ☐ Very low
2 ☐ Low
3 ☒ Normal
4 ☐ High
5 ☐ Very difficult

**Availability of information**

1 ☐ Very good
2 ☐ Good
3 ☐ Normal
4 ☒ Bad
5 ☐ Very bad

*RoB 2.4 Were these deviations likely to have affected the outcome?*

**Ease scale**

1 ☐ Very easy
2 ☐ Easy
3 ☐ Normal
4 ☒ Difficult
5 ☐ Very difficult

**Subjectivity scale**

1 ☐ Very low
2 ☐ Low
3 ☐ Normal
4 ☒ High
5 ☐ Very difficult

**Availability of information**

1 ☐ Very good
2 ☐ Good
3 ☐ Normal
4 ☒ Bad
5 ☐ Very bad

*RoB 2.5: Were these deviations from the intended intervention balanced between groups?*

| **Ease scale** | **Subjectivity scale** | **Availability of information** |
|---|---|---|
| 1 ☐ Very easy | 1 ☐ Very low | 1 ☐ Very good |
| 2 ☐ Easy | 2 ☐ Low | 2 ☐ Good |
| 3 ☐ Normal | 3 ☐ Normal | 3 ☐ Normal |
| 4 ☒ Difficult | 4 ☒ High | 4 ☒ Bad |
| 5 ☐ Very difficult | 5 ☐ Very high | 5 ☐ Very bad |

*RoB 2.6: Was an appropriate analysis used to estimate the effect of assignment to intervention?*

| **Ease scale** | **Subjectivity scale** | **Availability of information** |
|---|---|---|
| 1 ☐ Very easy | 1 ☐ Very low | 1 ☐ Very good |
| 2 ☒ Easy | 2 ☒ Low | 2 ☒ Good |
| 3 ☐ Normal | 3 ☐ Normal | 3 ☐ Normal |
| 4 ☐ Difficult | 4 ☐ High | 4 ☐ Bad |
| 5 ☐ Very difficult | 5 ☐ Very difficult | 5 ☐ Very bad |

*RoB 2.7 Was there potential for a substantial impact (on the result) of the failure to analyse participants in the group to which they were randomized?*

| **Ease scale** | **Subjectivity scale** | **Availability of information** |
|---|---|---|
| 1 ☐ Very easy | 1 ☐ Very low | 1 ☐ Very good |
| 2 ☐ Easy | 2 ☐ Low | 2 ☐ Good |
| 3 ☒ Normal | 3 ☒ Normal | 3 ☒ Normal |
| 4 ☐ Difficult | 4 ☐ High | 4 ☐ Bad |
| 5 ☐ Very difficult | 5 ☐ Very difficult | 5 ☐ Very bad |

## *Risk Domain 3*

*RoB 3.1 Were data for this outcome available for all, or nearly all, Participants randomized?*

| **Ease scale** | **Subjectivity scale** | **Availability of information** |
|---|---|---|
| 1 ☐ Very easy | 1 ☐ Very low | 1 ☐ Very good |
| 2 ☒ Easy | 2 ☒ Low | 2 ☐ Good |
| 3 ☐ Normal | 3 ☐ Normal | 3 ☒ Normal |
| 4 ☐ Difficult | 4 ☐ High | 4 ☐ Bad |
| 5 ☐ Very difficult | 5 ☐ Very difficult | 5 ☐ Very bad |

*RoB 3.2 Is there evidence that the result was not biased by missing outcome data?*

| **Ease scale** | **Subjectivity scale** | **Availability of information** |
|---|---|---|
| 1 ☐ Very easy | 1 ☐ Very low | 1 ☐ Very good |
| 2 ☐ Easy | 2 ☐ Low | 2 ☐ Good |
| 3 ☒ Normal | 3 ☒ Normal | 3 ☒ Normal |
| 4 ☐ Difficult | 4 ☐ High | 4 ☐ Bad |
| 5 ☐ Very difficult | 5 ☐ Very difficult | 5 ☐ Very bad |

*RoB 3.3 Could missingness in the outcome depend on its true value?*

| **Ease scale** | **Subjectivity scale** | **Availability of information** |
|---|---|---|
| 1 ☐ Very easy | 1 ☐ Very low | 1 ☐ Very good |
| 2 ☐ Easy | 2 ☐ Low | 2 ☐ Good |
| 3 ☐ Normal | 3 ☐ Normal | 3 ☐ Normal |
| 4 ☒ Difficult | 4 ☒ High | 4 ☒ Bad |
| 5 ☐ Very difficult | 5 ☐ Very difficult | 5 ☐ Very bad |

*RoB 3.4 Is it likely that missingness in the outcome depended on its true value?*

| **Ease scale** | **Subjectivity scale** | **Availability of information** |
|---|---|---|
| 1 ☐ Very easy | 1 ☐ Very low | 1 ☐ Very good |
| 2 ☐ Easy | 2 ☐ Low | 2 ☐ Good |
| 3 ☐ Normal | 3 ☐ Normal | 3 ☐ Normal |
| 4 ☒ Difficult | 4 ☐ High | 4 ☐ Bad |
| 5 ☐ Very difficult | 5 ☒ Very difficult | 5 ☒ Very bad |

## *Risk Domain 4*

*RoB 4.1 Was the method of measurement the outcome inappropriate?*

| **Ease scale** | **Subjectivity scale** | **Availability of information** |
|---|---|---|
| 1 ☐ Very easy | 1 ☐ Very low | 1 ☐ Very good |
| 2 ☐ Easy | 2 ☐ Low | 2 ☐ Good |
| 3 ☒ Normal | 3 ☒ Normal | 3 ☒ Normal |
| 4 ☐ Difficult | 4 ☐ High | 4 ☐ Bad |
| 5 ☐ Very difficult | 5 ☐ Very difficult | 5 ☐ Very bad |

*RoB 4.2 Could measurement or ascertainment of the outcome have differed between intervention groups?*

| **Ease scale** | **Subjectivity scale** | **Availability of information** |
|---|---|---|
| 1 ☐ Very easy | 1 ☐ Very low | 1 ☐ Very good |
| 2 ☐ Easy | 2 ☐ Low | 2 ☐ Good |
| 3 ☒ Normal | 3 ☒ Normal | 3 ☒ Normal |
| 4 ☐ Difficult | 4 ☐ High | 4 ☐ Bad |
| 5 ☐ Very difficult | 5 ☐ Very difficult | 5 ☐ Very bad |

*RoB 4.3 Were outcome assessors aware of the intervention received by study participants?*

| **Ease scale** | **Subjectivity scale** | **Availability of information** |
|---|---|---|
| 1 ☐ Very easy | 1 ☐ Very low | 1 ☐ Very good |
| 2 ☒ Easy | 2 ☒ Low | 2 ☒ Good |
| 3 ☐ Normal | 3 ☐ Normal | 3 ☐ Normal |
| 4 ☐ Difficult | 4 ☐ High | 4 ☐ Bad |
| 5 ☐ Very difficult | 5 ☐ Very difficult | 5 ☐ Very bad |

*RoB 4.4 Could assessment of the outcome have been influenced by knowledge of intervention received?*

| **Ease scale** | **Subjectivity scale** | **Availability of information** |
|---|---|---|
| 1 ☐ Very easy | 1 ☐ Very low | 1 ☐ Very good |
| 2 ☐ Easy | 2 ☐ Low | 2 ☐ Good |
| 3 ☒ Normal | 3 ☒ Normal | 3 ☒ Normal |
| 4 ☐ Difficult | 4 ☐ High | 4 ☐ Bad |
| 5 ☐ Very difficult | 5 ☐ Very difficult | 5 ☐ Very bad |

*RoB 4.5 Is it likely that the assessment of the outcome was influenced by knowledge of the intervention received?*

| **Ease scale** | **Subjectivity scale** | **Availability of information** |
|---|---|---|
| 1 ☐ Very easy | 1 ☐ Very low | 1 ☐ Very good |
| 2 ☐ Easy | 2 ☐ Low | 2 ☐ Good |
| 3 ☐ Normal | 3 ☐ Normal | 3 ☐ Normal |
| 4 ☒ Difficult | 4 ☐ High | 4 ☐ Bad |
| 5 ☐ Very difficult | 5 ☒ Very difficult | 5 ☒ Very bad |

## *Risk Domain 5*

*RoB 5.1 Were the data that produced this result analysed in accordance with a pre-specified analysis plan that was finalized before unblinded outcome data were available for analysis?*

| **Ease scale** | **Subjectivity scale** | **Availability of information** |
|---|---|---|
| 1 ☐ Very easy | 1 ☐ Very low | 1 ☐ Very good |
| 2 ☐ Easy | 2 ☐ Low | 2 ☒ Good |
| 3 ☒ Normal | 3 ☒ Normal | 3 ☐ Normal |
| 4 ☐ Difficult | 4 ☐ High | 4 ☐ Bad |
| 5 ☐ Very difficult | 5 ☐ Very difficult | 5 ☐ Very bad |

*RoB 5.2 Is the numerical result being assessed likely to have been selected, on the basis of the results, from multiple eligible outcome measurements (e.g. scales, definitions, time points) within the outcome domain?*

| **Ease scale** | **Subjectivity scale** | **Availability of information** |
|---|---|---|
| 1 ☐ Very easy | 1 ☐ Very low | 1 ☐ Very good |
| 2 ☐ Easy | 2 ☐ Low | 2 ☒ Good |
| 3 ☒ Normal | 3 ☒ Normal | 3 ☐ Normal |
| 4 ☐ Difficult | 4 ☐ High | 4 ☐ Bad |
| 5 ☐ Very difficult | 5 ☐ Very difficult | 5 ☐ Very bad |

*RoB 5.3 Is the numerical result being assessed likely to have been selected, on the basis of the results, from multiple eligible analyses of the data?*

| **Ease scale** | **Subjectivity scale** | **Availability of information** |
|---|---|---|
| 1 ☐ Very easy | 1 ☐ Very low | 1 ☐ Very good |
| 2 ☐ Easy | 2 ☐ Low | 2 ☐ Good |
| 3 ☒ Normal | 3 ☒ Normal | 3 ☒ Normal |
| 4 ☐ Difficult | 4 ☐ High | 4 ☐ Bad |
| 5 ☐ Very difficult | 5 ☐ Very difficult | 5 ☐ Very bad |

# *Difficulty: RoB annotation (Annotator 2)*

This section measures the **difficulty, subjectivity, and availability of information for assessing a RoB question** (RoB 1.1, RoB 1.2, ... RoB 5.3) in general. Select one of the five checkboxes based on the difficulty of that RoB question in the manual assessment. On a scale from 1-5, 1 is the easiest to assess, and 5 is the toughest to assess.

## *Risk Domain 1*

*RoB 1.1 Was the allocation sequence random?*

| **Ease scale** | **Subjectivity scale** | **Availability of information** |
|---|---|---|
| 1 ☐ Very easy | 1 ☐ Very easy | 1 ☐ Very easy |
| 2 ☐ Easy | 2 ☒ Easy | 2 ☒ Easy |
| 3 ☐ Normal | 3 ☐ Normal | 3 ☐ Normal |
| 4 ☐ Difficult | 4 ☐ Difficult | 4 ☐ Difficult |
| 5 ☐ Very difficult | 5 ☐ Very difficult | 5 ☐ Very difficult |

RoB 1.2 *Was the allocation sequence concealed until participants were enrolled and assigned to interventions?*

| **Ease scale** | **Subjectivity scale** | **Availability of information** |
|---|---|---|
| 1 ☒ Very easy | 1 ☐ Very easy | 1 ☐ Very easy |
| 2 ☐ Easy | 2 ☒ Easy | 2 ☒ Easy |
| 3 ☐ Normal | 3 ☐ Normal | 3 ☐ Normal |
| 4 ☐ Difficult | 4 ☐ Difficult | 4 ☐ Difficult |
| 5 ☐ Very difficult | 5 ☐ Very difficult | 5 ☐ Very difficult |

RoB 1.3 *Did baseline differences between intervention groups suggest a problem with the randomization process?*

| **Ease scale** | **Subjectivity scale** | **Availability of information** |
|---|---|---|
| 1 ☐ Very easy | 1 ☐ Very easy | 1 ☐ Very easy |
| 2 ☐ Easy | 2 ☐ Easy | 2 ☐ Easy |
| 3 ☐ Normal | 3 ☐ Normal | 3 ☒ Normal |
| 4 ☒ Difficult | 4 ☐ Difficult | 4 ☐ Difficult |
| 5 ☐ Very difficult | 5 ☒ Very difficult | 5 ☐ Very difficult |

## *Risk Domain 2*

RoB 2.1: *Were* ***participants*** *aware of their assigned intervention during the trial?*

| **Ease scale** | **Subjectivity scale** | **Availability of information** |
|---|---|---|
| 1 ☐ Very easy | 1 ☐ Very easy | 1 ☐ Very easy |
| 2 ☒ Easy | 2 ☒ Easy | 2 ☒ Easy |
| 3 ☐ Normal | 3 ☐ Normal | 3 ☐ Normal |
| 4 ☐ Difficult | 4 ☐ Difficult | 4 ☐ Difficult |
| 5 ☐ Very difficult | 5 ☐ Very difficult | 5 ☐ Very difficult |

RoB 2.2: *Were carers and people delivering the interventions aware of participants' assigned intervention during the trial?*

| **Ease scale** | **Subjectivity scale** | **Availability of information** |
|---|---|---|
| 1 ☐ Very easy | 1 ☐ Very easy | 1 ☐ Very easy |
| 2 ☒ Easy | 2 ☒ Easy | 2 ☒ Easy |
| 3 ☐ Normal | 3 ☐ Normal | 3 ☐ Normal |
| 4 ☐ Difficult | 4 ☐ Difficult | 4 ☐ Difficult |
| 5 ☐ Very difficult | 5 ☐ Very difficult | 5 ☐ Very difficult |

*RoB 2.3: Were there deviations from the intended intervention that arose because of the trial context?*

| **Ease scale** | **Subjectivity scale** | **Availability of information** |
|---|---|---|
| 1 ☐ Very easy | 1 ☐ Very easy | 1 ☐ Very easy |
| 2 ☐ Easy | 2 ☐ Easy | 2 ☐ Easy |
| 3 ☐ Normal | 3 ☐ Normal | 3 ☐ Normal |
| 4 ☐ Difficult | 4 ☐ Difficult | 4 ☐ Difficult |
| 5 ☒ Very difficult | 5 ☒ Very difficult | 5 ☒ Very difficult |

*RoB 2.4 Were these deviations likely to have affected the outcome?*

| **Ease scale** | **Subjectivity scale** | **Availability of information** |
|---|---|---|
| 1 ☐ Very easy | 1 ☐ Very easy | 1 ☐ Very easy |
| 2 ☐ Easy | 2 ☐ Easy | 2 ☐ Easy |
| 3 ☐ Normal | 3 ☐ Normal | 3 ☐ Normal |
| 4 ☐ Difficult | 4 ☐ Difficult | 4 ☐ Difficult |
| 5 ☒ Very difficult | 5 ☒ Very difficult | 5 ☒ Very difficult |

*RoB 2.5: Were these deviations from the intended intervention balanced between groups?*

| **Ease scale** | **Subjectivity scale** | **Availability of information** |
|---|---|---|
| 1 ☐ Very easy | 1 ☐ Very easy | 1 ☐ Very easy |
| 2 ☒ Easy | 2 ☒ Easy | 2 ☐ Easy |
| 3 ☐ Normal | 3 ☐ Normal | 3 ☐ Normal |
| 4 ☐ Difficult | 4 ☐ Difficult | 4 ☒ Difficult |
| 5 ☐ Very difficult | 5 ☐ Very difficult | 5 ☐ Very difficult |

*RoB 2.6: Was an appropriate analysis used to estimate the effect of assignment to intervention?*

| **Ease scale** | **Subjectivity scale** | **Availability of information** |
|---|---|---|
| 1 ☐ Very easy | 1 ☐ Very easy | 1 ☐ Very easy |
| 2 ☒ Easy | 2 ☒ Easy | 2 ☒ Easy |
| 3 ☐ Normal | 3 ☐ Normal | 3 ☐ Normal |
| 4 ☐ Difficult | 4 ☐ Difficult | 4 ☐ Difficult |
| 5 ☐ Very difficult | 5 ☐ Very difficult | 5 ☐ Very difficult |

*RoB 2.7 Was there potential for a substantial impact (on the result) of the failure to analyse participants in the group to which they were randomized?*

| **Ease scale** | **Subjectivity scale** | **Availability of information** |
|---|---|---|
| 1 ☐ Very easy | 1 ☐ Very easy | 1 ☐ Very easy |
| 2 ☐ Easy | 2 ☐ Easy | 2 ☐ Easy |
| 3 ☐ Normal | 3 ☐ Normal | 3 ☐ Normal |
| 4 ☒ Difficult | 4 ☐ Difficult | 4 ☐ Difficult |
| 5 ☐ Very difficult | 5 ☒ Very difficult | 5 ☒ Very difficult |

## *Risk Domain 3*

*RoB 3.1 Were data for this outcome available for all, or nearly all, Participants randomized?*

| **Ease scale** | **Subjectivity scale** | **Availability of information** |
|---|---|---|
| 1 ☐ Very easy | 1 ☐ Very easy | 1 ☐ Very easy |
| 2 ☐ Easy | 2 ☐ Easy | 2 ☐ Easy |
| 3 ☒ Normal | 3 ☐ Normal | 3 ☒ Normal |
| 4 ☐ Difficult | 4 ☒ Difficult | 4 ☐ Difficult |
| 5 ☐ Very difficult | 5 ☐ Very difficult | 5 ☐ Very difficult |

*RoB 3.2 Is there evidence that the result was not biased by missing outcome data?*

| **Ease scale** | **Subjectivity scale** | **Availability of information** |
|---|---|---|
| 1 ☐ Very easy | 1 ☐ Very easy | 1 ☐ Very easy |
| 2 ☐ Easy | 2 ☐ Easy | 2 ☐ Easy |
| 3 ☐ Normal | 3 ☐ Normal | 3 ☐ Normal |
| 4 ☒ Difficult | 4 ☒ Difficult | 4 ☐ Difficult |
| 5 ☐ Very difficult | 5 ☐ Very difficult | 5 ☒ Very difficult |

*RoB 3.3 Could missingness in the outcome depend on its true value?*

| **Ease scale** | **Subjectivity scale** | **Availability of information** |
|---|---|---|
| 1 ☐ Very easy | 1 ☐ Very easy | 1 ☐ Very easy |
| 2 ☒ Easy | 2 ☐ Easy | 2 ☐ Easy |
| 3 ☐ Normal | 3 ☐ Normal | 3 ☐ Normal |
| 4 ☐ Difficult | 4 ☒ Difficult | 4 ☒ Difficult |
| 5 ☐ Very difficult | 5 ☐ Very difficult | 5 ☐ Very difficult |

*RoB 3.4 Is it likely that missingness in the outcome depended on its true value?*

| **Ease scale** | **Subjectivity scale** | **Availability of information** |
|---|---|---|
| 1 ☐ Very easy | 1 ☐ Very easy | 1 ☐ Very easy |
| 2 ☐ Easy | 2 ☐ Easy | 2 ☐ Easy |
| 3 ☐ Normal | 3 ☐ Normal | 3 ☐ Normal |
| 4 ☐ Difficult | 4 ☐ Difficult | 4 ☐ Difficult |
| 5 ☒ Very difficult | 5 ☒ Very difficult | 5 ☒ Very difficult |

## *Risk Domain 4*

*RoB 4.1 Was the method of measurement the outcome inappropriate?*

| **Ease scale** | **Subjectivity scale** | **Availability of information** |
|---|---|---|
| 1 ☐ Very easy | 1 ☐ Very easy | 1 ☐ Very easy |
| 2 ☒ Easy | 2 ☒ Easy | 2 ☐ Easy |
| 3 ☐ Normal | 3 ☐ Normal | 3 ☐ Normal |
| 4 ☐ Difficult | 4 ☐ Difficult | 4 ☒ Difficult |
| 5 ☐ Very difficult | 5 ☐ Very difficult | 5 ☐ Very difficult |

*RoB 4.2 Could measurement or ascertainment of the outcome have differed between intervention groups?*

| Ease scale | Subjectivity scale | Availability of information |
|---|---|---|
| 1 ☐ Very easy | 1 ☐ Very easy | 1 ☐ Very easy |
| 2 ☐ Easy | 2 ☐ Easy | 2 ☐ Easy |
| 3 ☐ Normal | 3 ☐ Normal | 3 ☐ Normal |
| 4 ☒ Difficult | 4 ☒ Difficult | 4 ☐ Difficult |
| 5 ☐ Very difficult | 5 ☐ Very difficult | 5 ☒ Very difficult |

*RoB 4.3 Were outcome assessors aware of the intervention received by study participants?*

| Ease scale | Subjectivity scale | Availability of information |
|---|---|---|
| 1 ☒ Very easy | 1 ☐ Very easy | 1 ☐ Very easy |
| 2 ☐ Easy | 2 ☐ Easy | 2 ☐ Easy |
| 3 ☐ Normal | 3 ☒ Normal | 3 ☒ Normal |
| 4 ☐ Difficult | 4 ☐ Difficult | 4 ☐ Difficult |
| 5 ☐ Very difficult | 5 ☐ Very difficult | 5 ☐ Very difficult |

*RoB 4.4 Could assessment of the outcome have been influenced by knowledge of intervention received?*

| Ease scale | Subjectivity scale | Availability of information |
|---|---|---|
| 1 ☐ Very easy | 1 ☐ Very easy | 1 ☐ Very easy |
| 2 ☐ Easy | 2 ☐ Easy | 2 ☐ Easy |
| 3 ☒ Normal | 3 ☐ Normal | 3 ☐ Normal |
| 4 ☐ Difficult | 4 ☒ Difficult | 4 ☐ Difficult |
| 5 ☐ Very difficult | 5 ☐ Very difficult | 5 ☒ Very difficult |

*RoB 4.5 Is it likely that the assessment of the outcome was influenced by knowledge of the intervention received?*

| Ease scale | Subjectivity scale | Availability of information |
|---|---|---|
| 1 ☐ Very easy | 1 ☐ Very easy | 1 ☐ Very easy |
| 2 ☐ Easy | 2 ☐ Easy | 2 ☐ Easy |
| 3 ☒ Normal | 3 ☐ Normal | 3 ☒ Normal |
| 4 ☐ Difficult | 4 ☒ Difficult | 4 ☐ Difficult |
| 5 ☐ Very difficult | 5 ☐ Very difficult | 5 ☐ Very difficult |

## *Risk Domain 5*

*RoB 5.1 Were the data that produced this result analysed in accordance with a pre-specified analysis plan that was finalized before unblinded outcome data were available for analysis?*

| **Ease scale** | **Subjectivity scale** | **Availability of information** |
|---|---|---|
| 1 ☐ Very easy | 1 ☐ Very easy | 1 ☐ Very easy |
| 2 ☐ Easy | 2 ☐ Easy | 2 ☐ Easy |
| 3 ☐ Normal | 3 ☐ Normal | 3 ☐ Normal |
| 4 ☒ Difficult | 4 ☒ Difficult | 4 ☒ Difficult |
| 5 ☐ Very difficult | 5 ☐ Very difficult | 5 ☐ Very difficult |

*RoB 5.2 Is the numerical result being assessed likely to have been selected, on the basis of the results, from multiple eligible outcome measurements (e.g. scales, definitions, time points) within the outcome domain?*

| **Ease scale** | **Subjectivity scale** | **Availability of information** |
|---|---|---|
| 1 ☐ Very easy | 1 ☐ Very easy | 1 ☐ Very easy |
| 2 ☐ Easy | 2 ☐ Easy | 2 ☐ Easy |
| 3 ☒ Normal | 3 ☒ Normal | 3 ☐ Normal |
| 4 ☐ Difficult | 4 ☐ Difficult | 4 ☒ Difficult |
| 5 ☐ Very difficult | 5 ☐ Very difficult | 5 ☐ Very difficult |

*RoB 5.3 Is the numerical result being assessed likely to have been selected, on the basis of the results, from multiple eligible analyses of the data?*

| **Ease scale** | **Subjectivity scale** | **Availability of information** |
|---|---|---|
| 1 ☐ Very easy | 1 ☐ Very easy | 1 ☐ Very easy |
| 2 ☐ Easy | 2 ☐ Easy | 2 ☐ Easy |
| 3 ☐ Normal | 3 ☐ Normal | 3 ☐ Normal |
| 4 ☒ Difficult | 4 ☒ Difficult | 4 ☐ Difficult |
| 5 ☐ Very difficult | 5 ☐ Very difficult | 5 ☒ Very difficult |
