# Supplementary material for: RoBuster—Corpus Annotated With Risk of Bias Text Spans in Randomized Controlled Trials in Physiotherapy and Rehabilitation: Corpus Development and Annotation Study
Source: JMIR Form Res. 2026 Apr 27;10:e55127. doi: 10.2196/55127 (PMC13120535; doi:10.2196/55127)
Supplement: Multimedia Appendix 3 [file formative-v10-e55127-s003.pptx]

## Slide 1
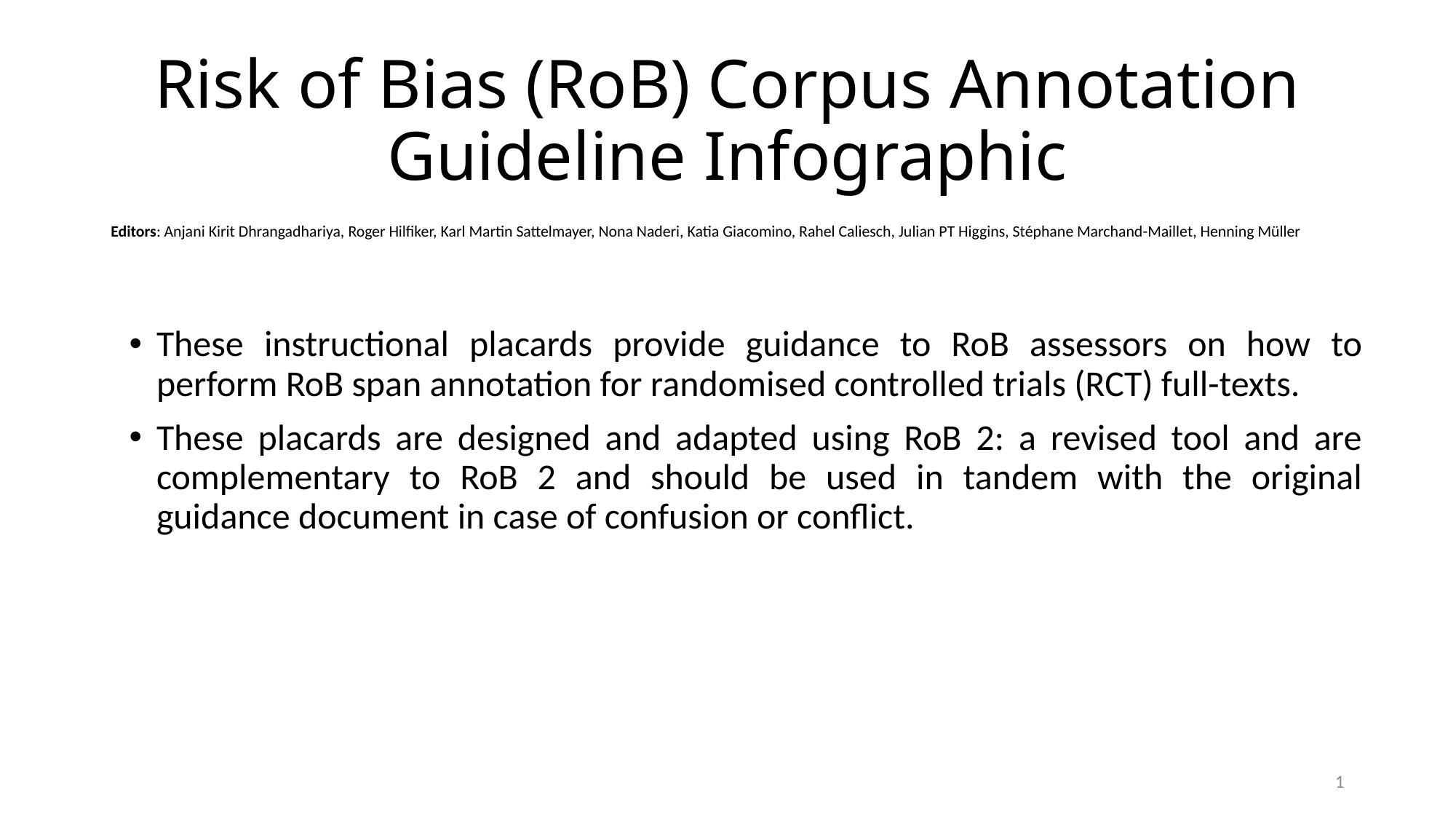

# Risk of Bias (RoB) Corpus Annotation Guideline Infographic
Editors: Anjani Kirit Dhrangadhariya, Roger Hilfiker, Karl Martin Sattelmayer, Nona Naderi, Katia Giacomino, Rahel Caliesch, Julian PT Higgins, Stéphane Marchand-Maillet, Henning Müller
These instructional placards provide guidance to RoB assessors on how to perform RoB span annotation for randomised controlled trials (RCT) full-texts.
These placards are designed and adapted using RoB 2: a revised tool and are complementary to RoB 2 and should be used in tandem with the original guidance document in case of confusion or conflict.
1

## Slide 2
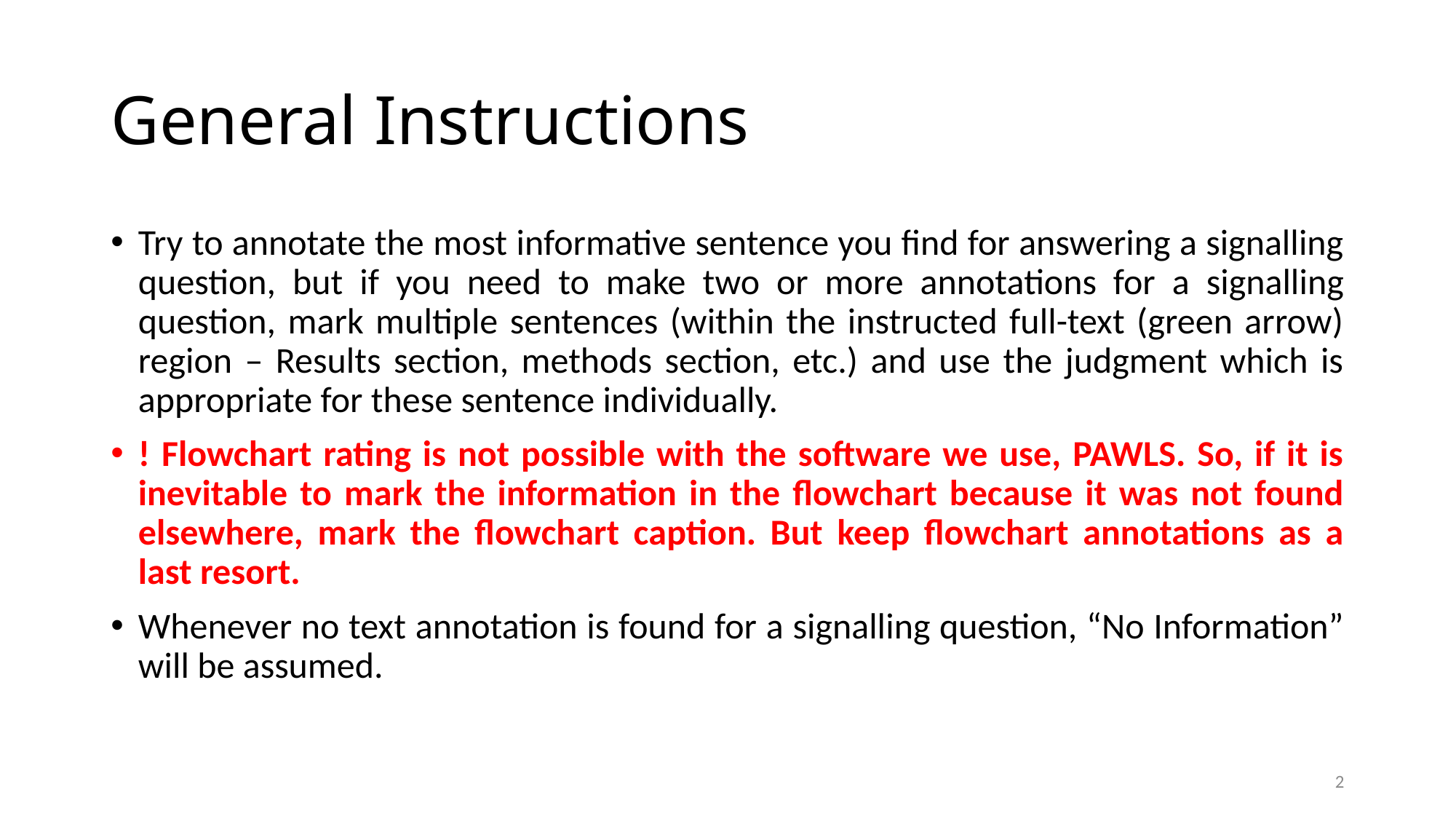

# General Instructions
Try to annotate the most informative sentence you find for answering a signalling question, but if you need to make two or more annotations for a signalling question, mark multiple sentences (within the instructed full-text (green arrow) region – Results section, methods section, etc.) and use the judgment which is appropriate for these sentence individually.
! Flowchart rating is not possible with the software we use, PAWLS. So, if it is inevitable to mark the information in the flowchart because it was not found elsewhere, mark the flowchart caption. But keep flowchart annotations as a last resort.
Whenever no text annotation is found for a signalling question, “No Information” will be assumed.
2

## Slide 3
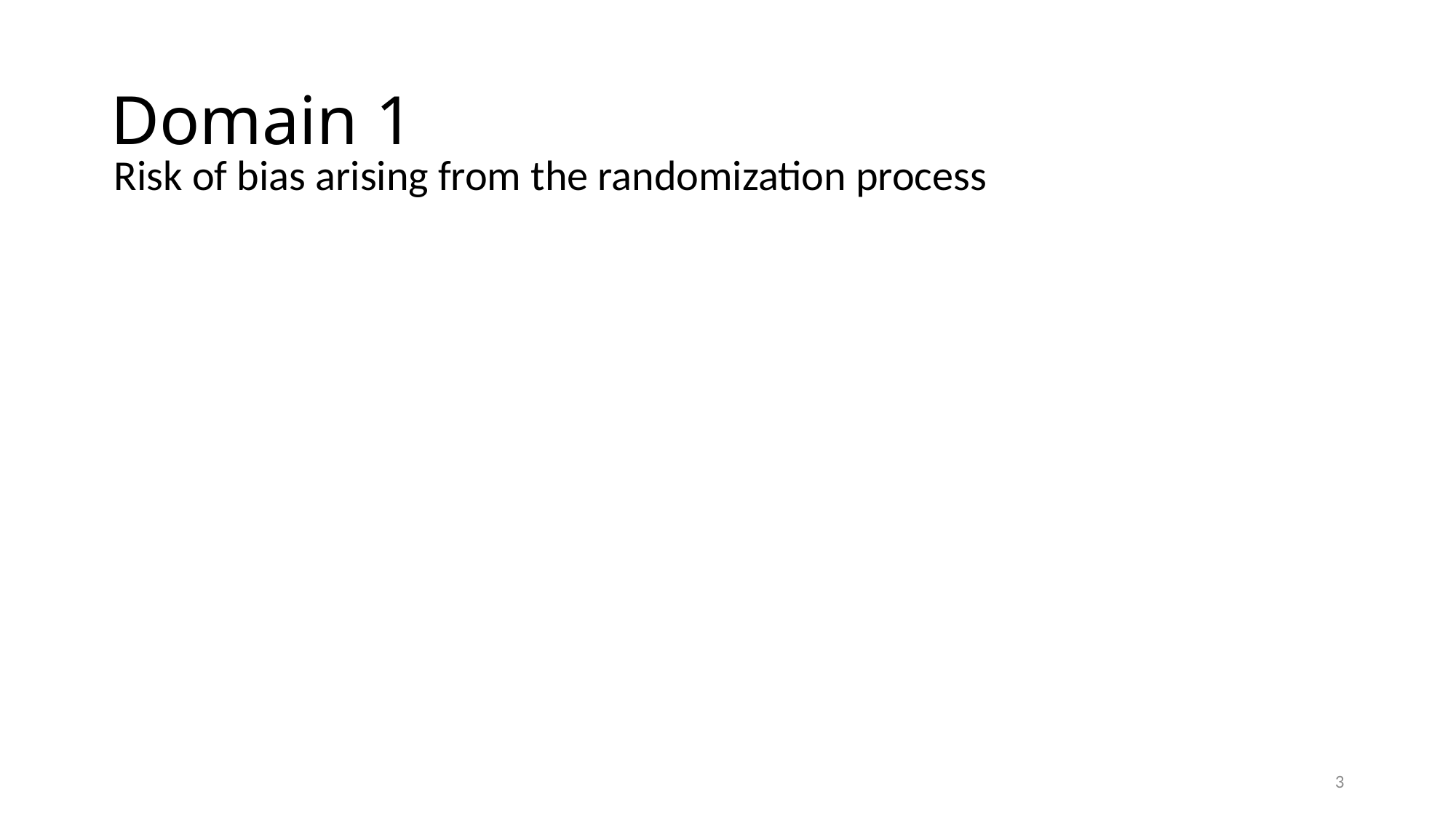

# Domain 1
Risk of bias arising from the randomization process
3

## Slide 4
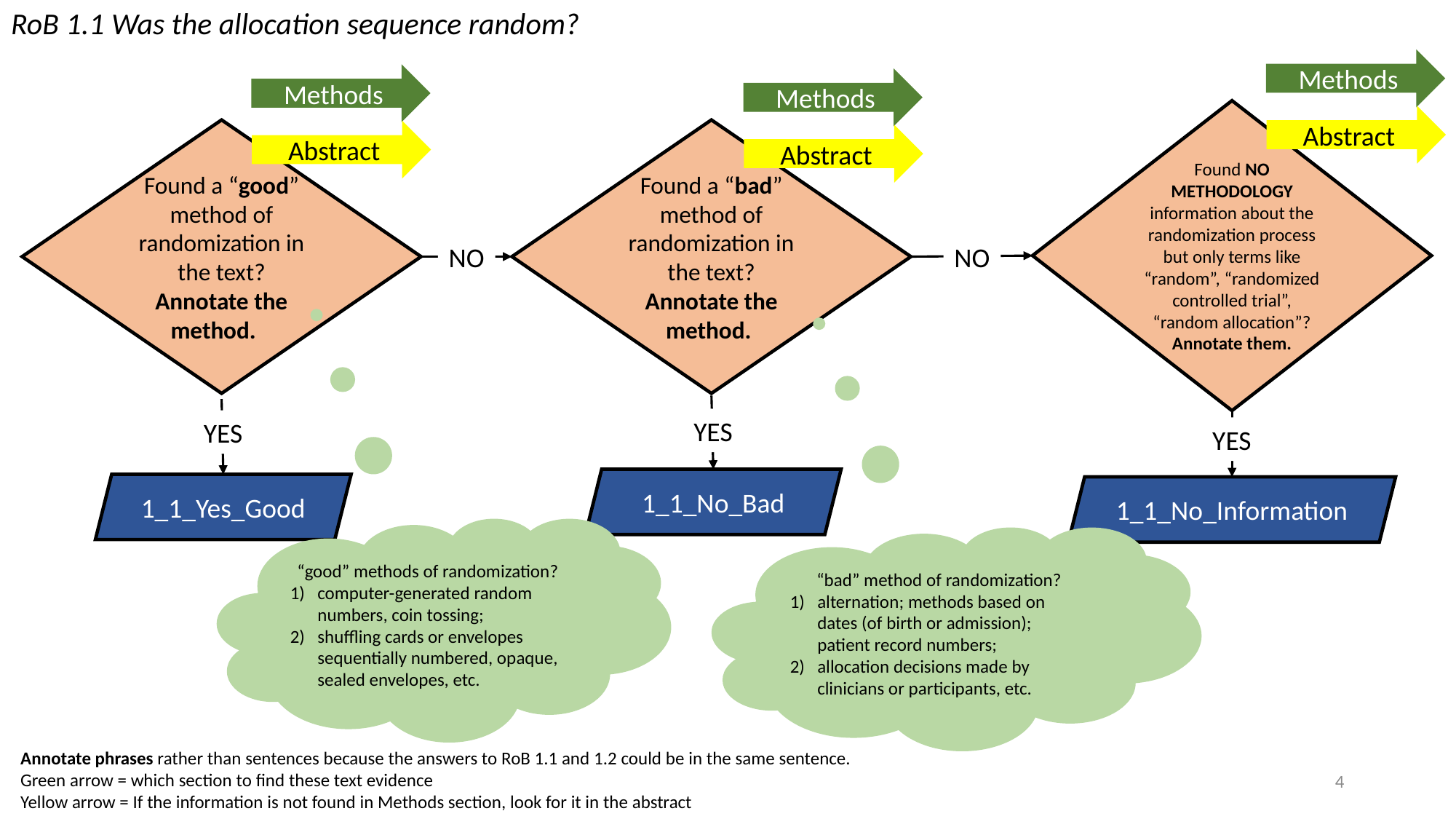

RoB 1.1 Was the allocation sequence random?
Methods
Methods
Methods
Found NO METHODOLOGY information about the randomization process but only terms like “random”, “randomized controlled trial”, “random allocation”? Annotate them.
Abstract
Found a “good” method of randomization in the text? Annotate the method.
Found a “bad” method of randomization in the text? Annotate the method.
Abstract
Abstract
NO
NO
YES
YES
YES
1_1_No_Bad
1_1_Yes_Good
1_1_No_Information
“good” methods of randomization?
computer-generated random numbers, coin tossing;
shuffling cards or envelopes sequentially numbered, opaque, sealed envelopes, etc.
“bad” method of randomization?
alternation; methods based on dates (of birth or admission); patient record numbers;
allocation decisions made by clinicians or participants, etc.
Annotate phrases rather than sentences because the answers to RoB 1.1 and 1.2 could be in the same sentence.
Green arrow = which section to find these text evidence
Yellow arrow = If the information is not found in Methods section, look for it in the abstract
4

## Slide 5
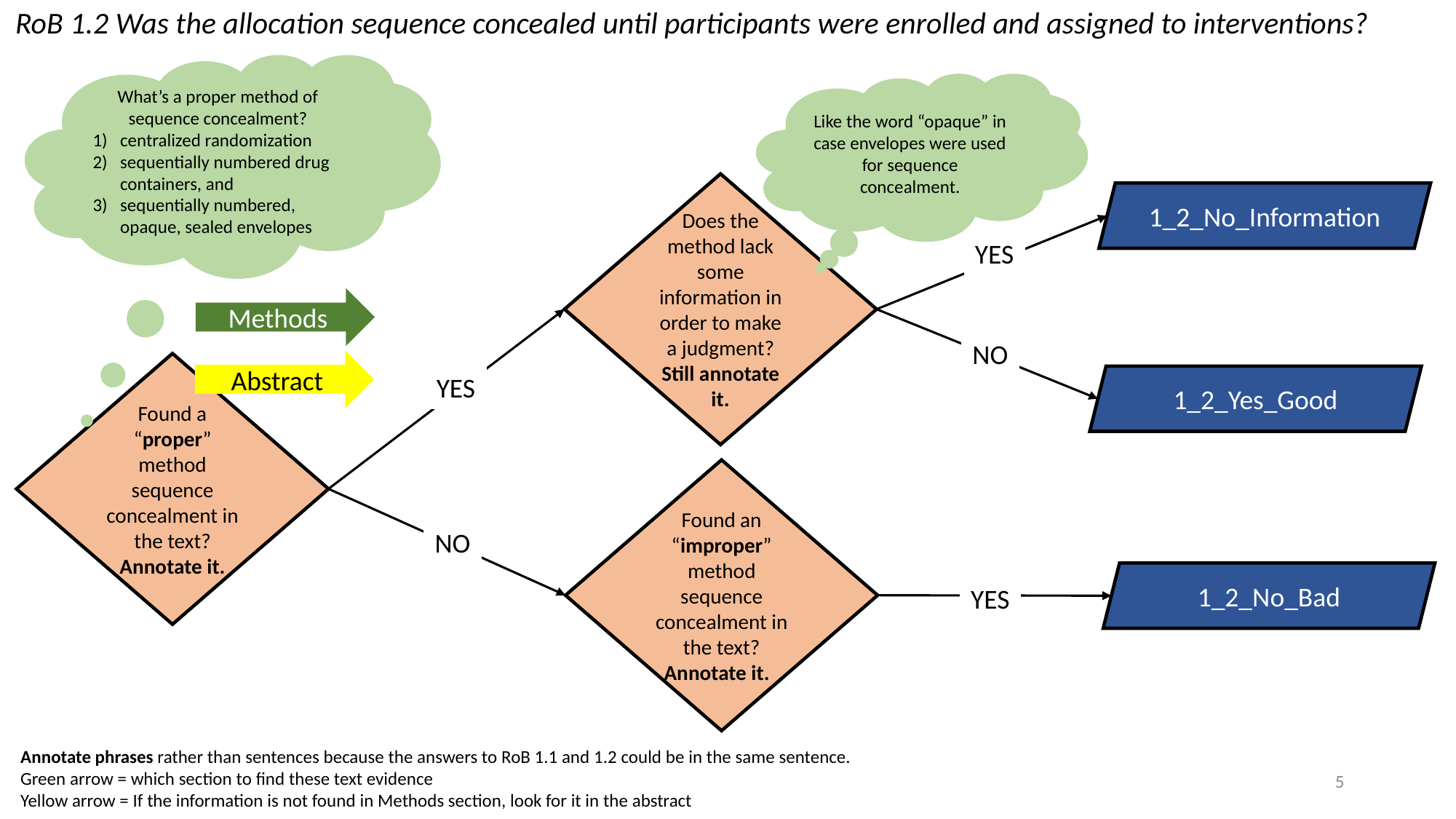

RoB 1.2 Was the allocation sequence concealed until participants were enrolled and assigned to interventions?
What’s a proper method of sequence concealment?
centralized randomization
sequentially numbered drug containers, and
sequentially numbered, opaque, sealed envelopes
Like the word “opaque” in case envelopes were used for sequence concealment.
Does the method lack some information in order to make a judgment? Still annotate it.
1_2_No_Information
YES
Methods
NO
Abstract
Found a “proper” method sequence concealment in the text? Annotate it.
YES
1_2_Yes_Good
Found an “improper” method sequence concealment in the text? Annotate it.
NO
1_2_No_Bad
YES
Annotate phrases rather than sentences because the answers to RoB 1.1 and 1.2 could be in the same sentence.
Green arrow = which section to find these text evidence
Yellow arrow = If the information is not found in Methods section, look for it in the abstract
5

## Slide 6
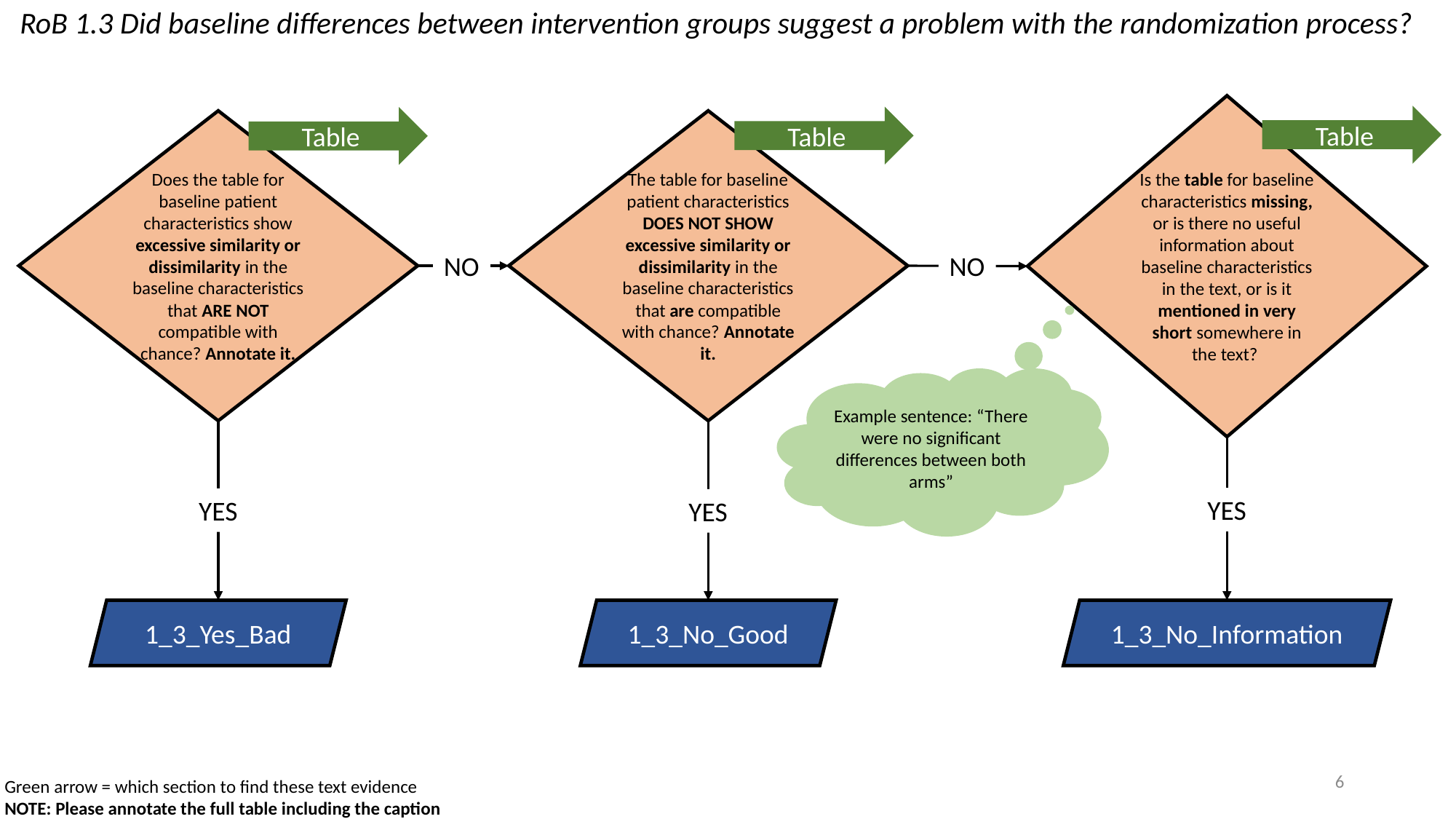

RoB 1.3 Did baseline differences between intervention groups suggest a problem with the randomization process?
Is the table for baseline characteristics missing, or is there no useful information about baseline characteristics in the text, or is it mentioned in very short somewhere in the text?
Table
Table
Table
Does the table for baseline patient characteristics show excessive similarity or dissimilarity in the baseline characteristics that ARE NOT compatible with chance? Annotate it.
The table for baseline patient characteristics DOES NOT SHOW excessive similarity or dissimilarity in the baseline characteristics that are compatible with chance? Annotate it.
NO
NO
Example sentence: “There were no significant differences between both arms”
YES
YES
YES
1_3_Yes_Bad
1_3_No_Good
1_3_No_Information
6
Green arrow = which section to find these text evidence
NOTE: Please annotate the full table including the caption

## Slide 7
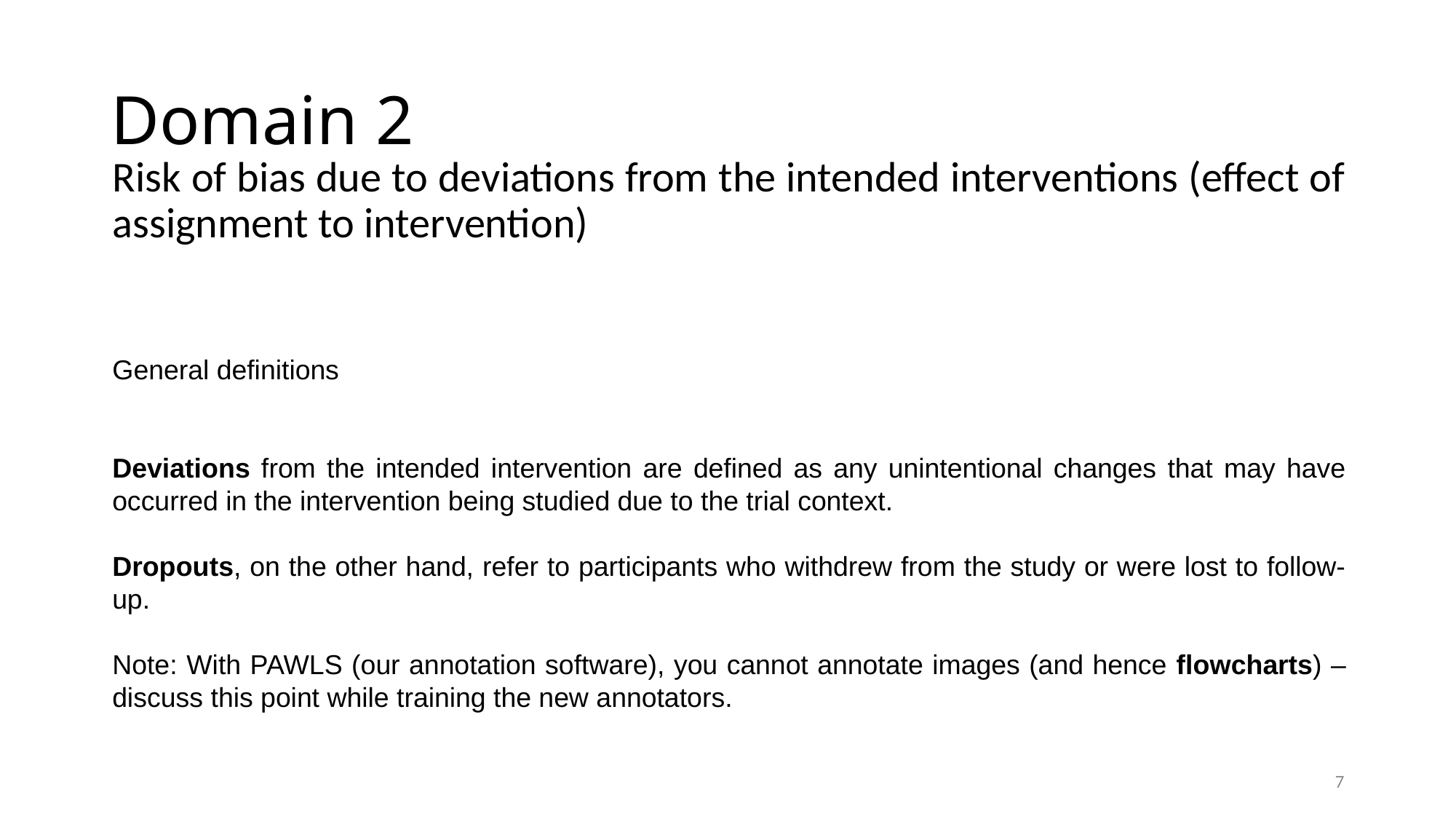

# Domain 2
Risk of bias due to deviations from the intended interventions (effect of assignment to intervention)
General definitions
Deviations from the intended intervention are defined as any unintentional changes that may have occurred in the intervention being studied due to the trial context.
Dropouts, on the other hand, refer to participants who withdrew from the study or were lost to follow-up.
Note: With PAWLS (our annotation software), you cannot annotate images (and hence flowcharts) – discuss this point while training the new annotators.
7

## Slide 8
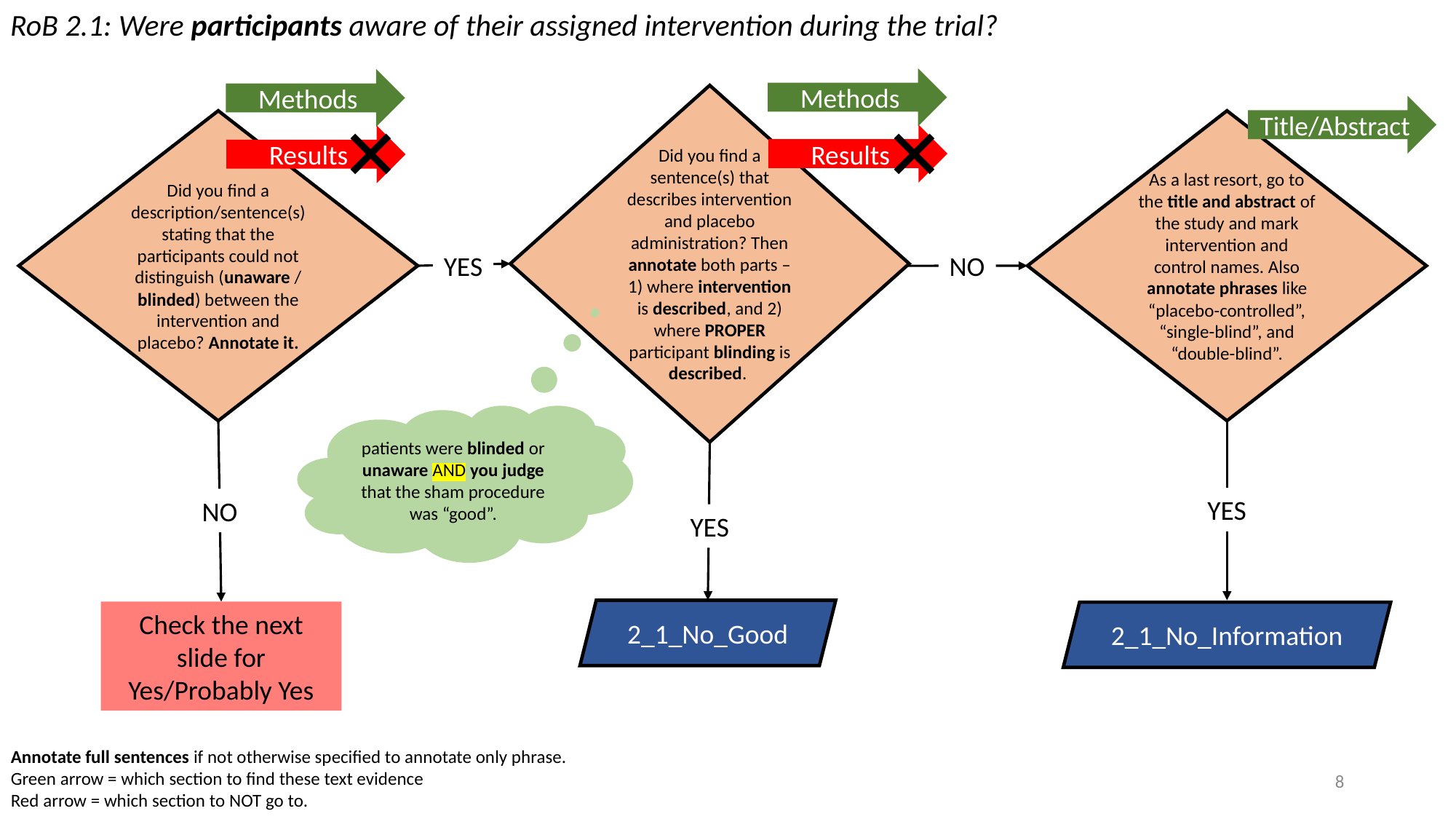

RoB 2.1: Were participants aware of their assigned intervention during the trial?
Methods
Methods
Did you find a sentence(s) that describes intervention and placebo administration? Then annotate both parts – 1) where intervention is described, and 2) where PROPER participant blinding is described.
Title/Abstract
Did you find a description/sentence(s) stating that the participants could not distinguish (unaware / blinded) between the intervention and placebo? Annotate it.
As a last resort, go to the title and abstract of the study and mark intervention and control names. Also annotate phrases like “placebo-controlled”, “single-blind”, and “double-blind”.
Results
Results
YES
NO
patients were blinded or unaware AND you judge that the sham procedure was “good”.
YES
NO
YES
2_1_No_Good
Check the next slide for Yes/Probably Yes
2_1_No_Information
Annotate full sentences if not otherwise specified to annotate only phrase.
Green arrow = which section to find these text evidence
Red arrow = which section to NOT go to.
8

## Slide 9
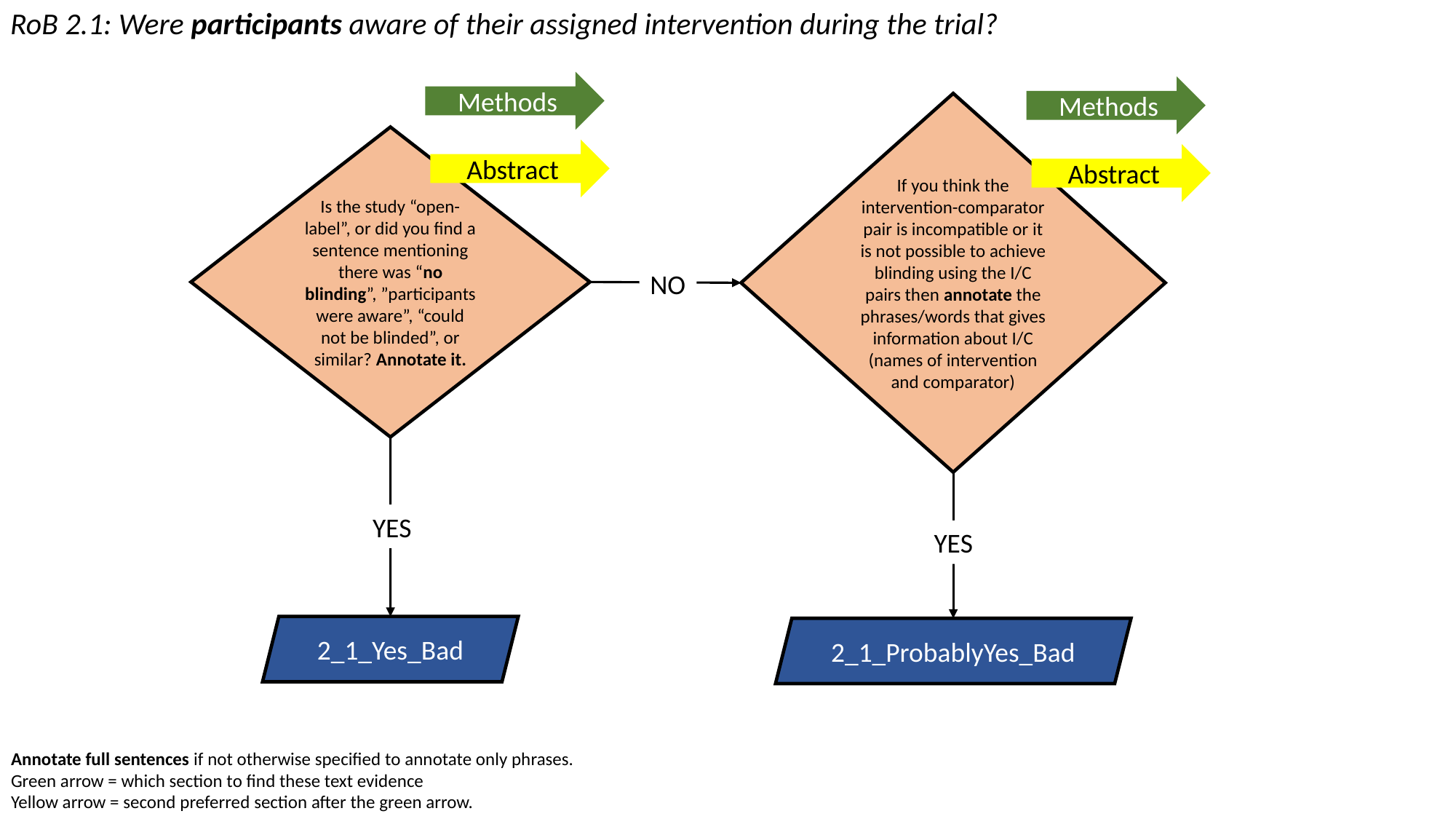

RoB 2.1: Were participants aware of their assigned intervention during the trial?
Methods
Methods
If you think the intervention-comparator pair is incompatible or it is not possible to achieve blinding using the I/C pairs then annotate the phrases/words that gives information about I/C (names of intervention and comparator)
Is the study “open-label”, or did you find a sentence mentioning there was “no blinding”, ”participants were aware”, “could not be blinded”, or similar? Annotate it.
Abstract
Abstract
NO
YES
YES
2_1_Yes_Bad
2_1_ProbablyYes_Bad
Annotate full sentences if not otherwise specified to annotate only phrases.
Green arrow = which section to find these text evidence
Yellow arrow = second preferred section after the green arrow.

## Slide 10
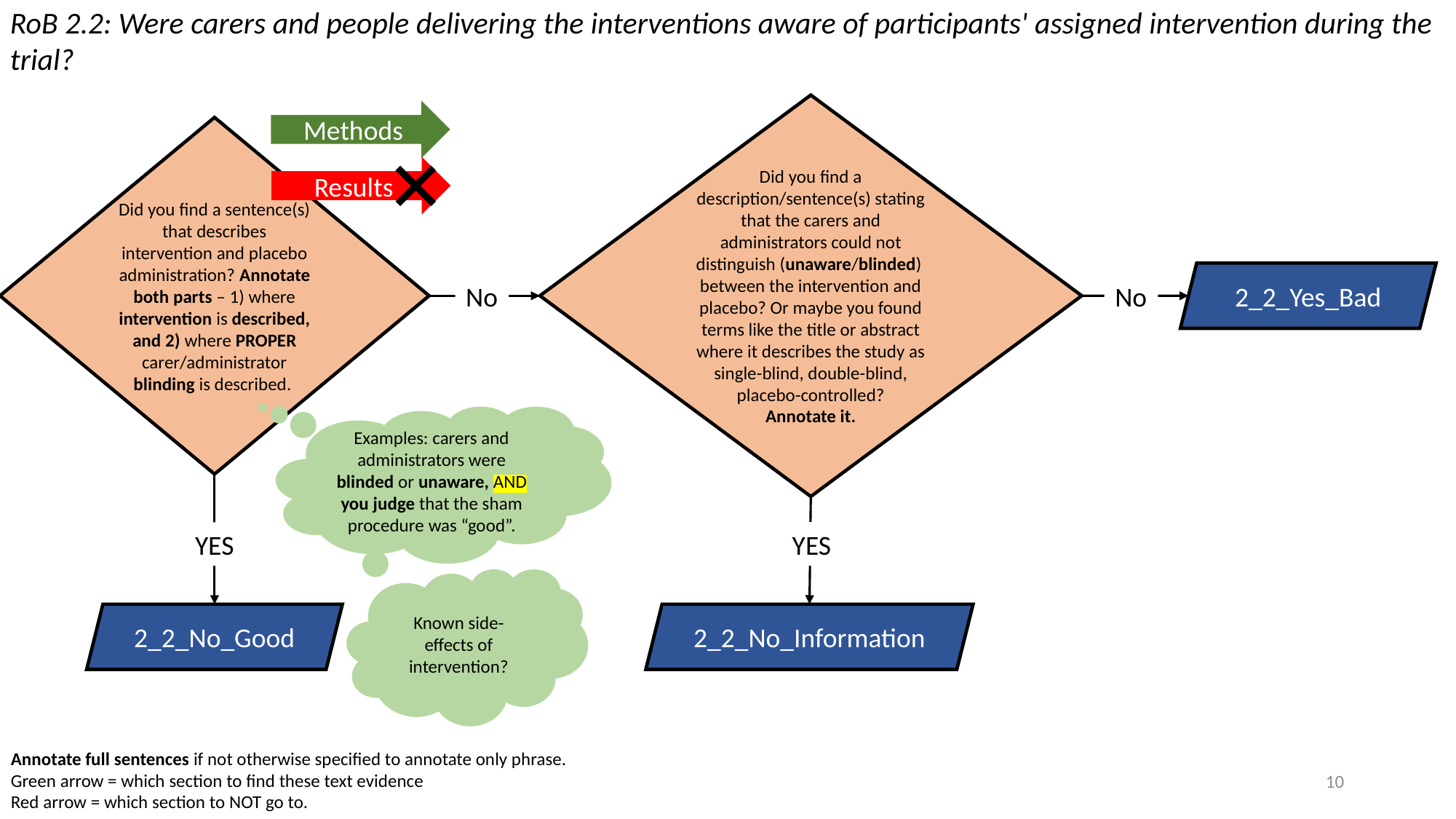

RoB 2.2: Were carers and people delivering the interventions aware of participants' assigned intervention during the trial?
Did you find a description/sentence(s) stating that the carers and administrators could not distinguish (unaware/blinded) between the intervention and placebo? Or maybe you found terms like the title or abstract where it describes the study as single-blind, double-blind, placebo-controlled?
Annotate it.
Methods
Did you find a sentence(s) that describes intervention and placebo administration? Annotate both parts – 1) where intervention is described, and 2) where PROPER carer/administrator blinding is described.
Results
2_2_Yes_Bad
No
No
Examples: carers and administrators were blinded or unaware, AND you judge that the sham procedure was “good”.
YES
YES
Known side-effects of intervention?
2_2_No_Good
2_2_No_Information
Annotate full sentences if not otherwise specified to annotate only phrase.
Green arrow = which section to find these text evidence
Red arrow = which section to NOT go to.
10

## Slide 11
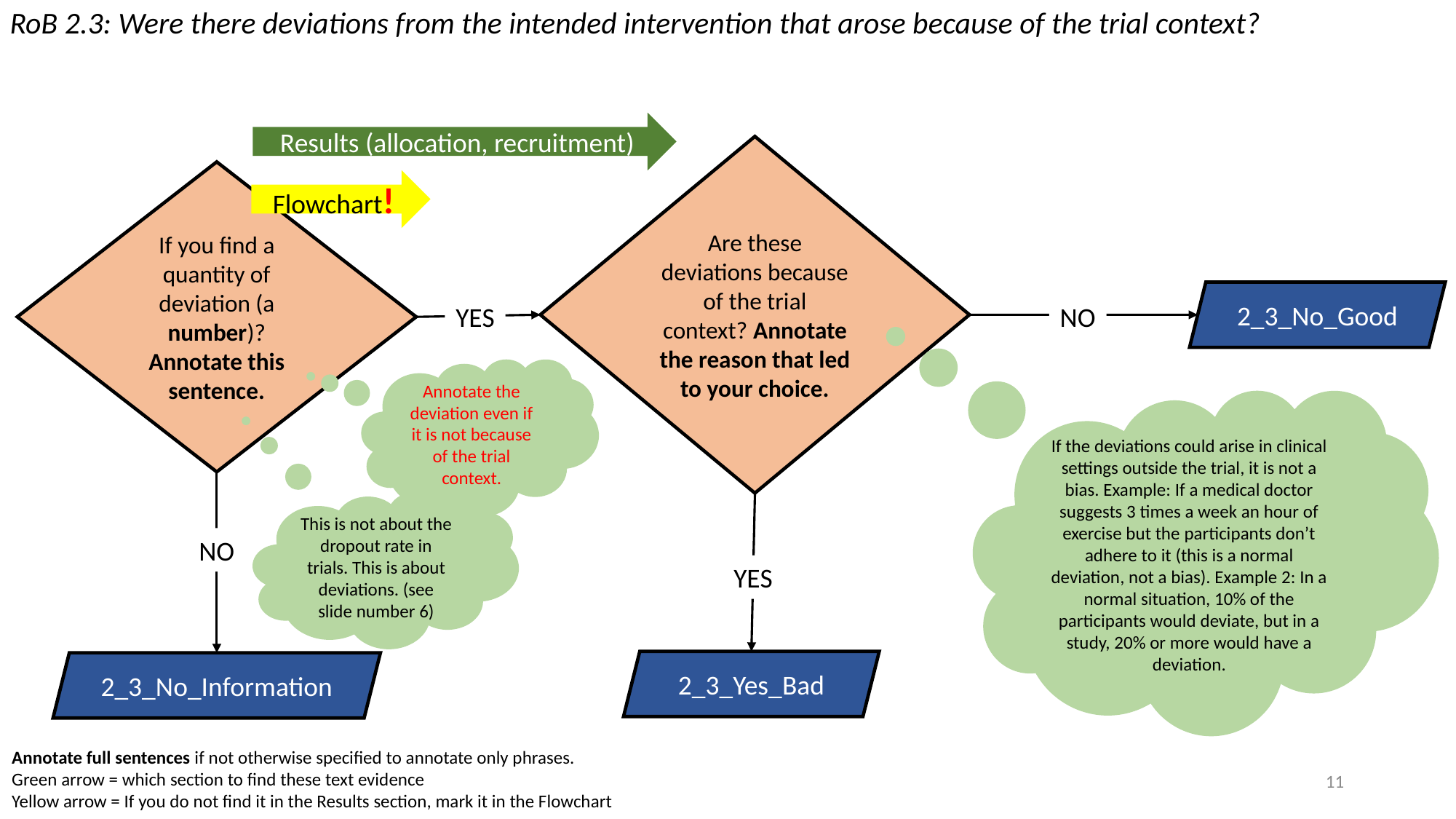

RoB 2.3: Were there deviations from the intended intervention that arose because of the trial context?
Results (allocation, recruitment)
Are these deviations because of the trial context? Annotate the reason that led to your choice.
If you find a quantity of deviation (a number)? Annotate this sentence.
Flowchart!
2_3_No_Good
NO
YES
Annotate the deviation even if it is not because of the trial context.
If the deviations could arise in clinical settings outside the trial, it is not a bias. Example: If a medical doctor suggests 3 times a week an hour of exercise but the participants don’t adhere to it (this is a normal deviation, not a bias). Example 2: In a normal situation, 10% of the participants would deviate, but in a study, 20% or more would have a deviation.
This is not about the dropout rate in trials. This is about deviations. (see slide number 6)
NO
YES
2_3_Yes_Bad
2_3_No_Information
Annotate full sentences if not otherwise specified to annotate only phrases.
Green arrow = which section to find these text evidence
Yellow arrow = If you do not find it in the Results section, mark it in the Flowchart
11

## Slide 12
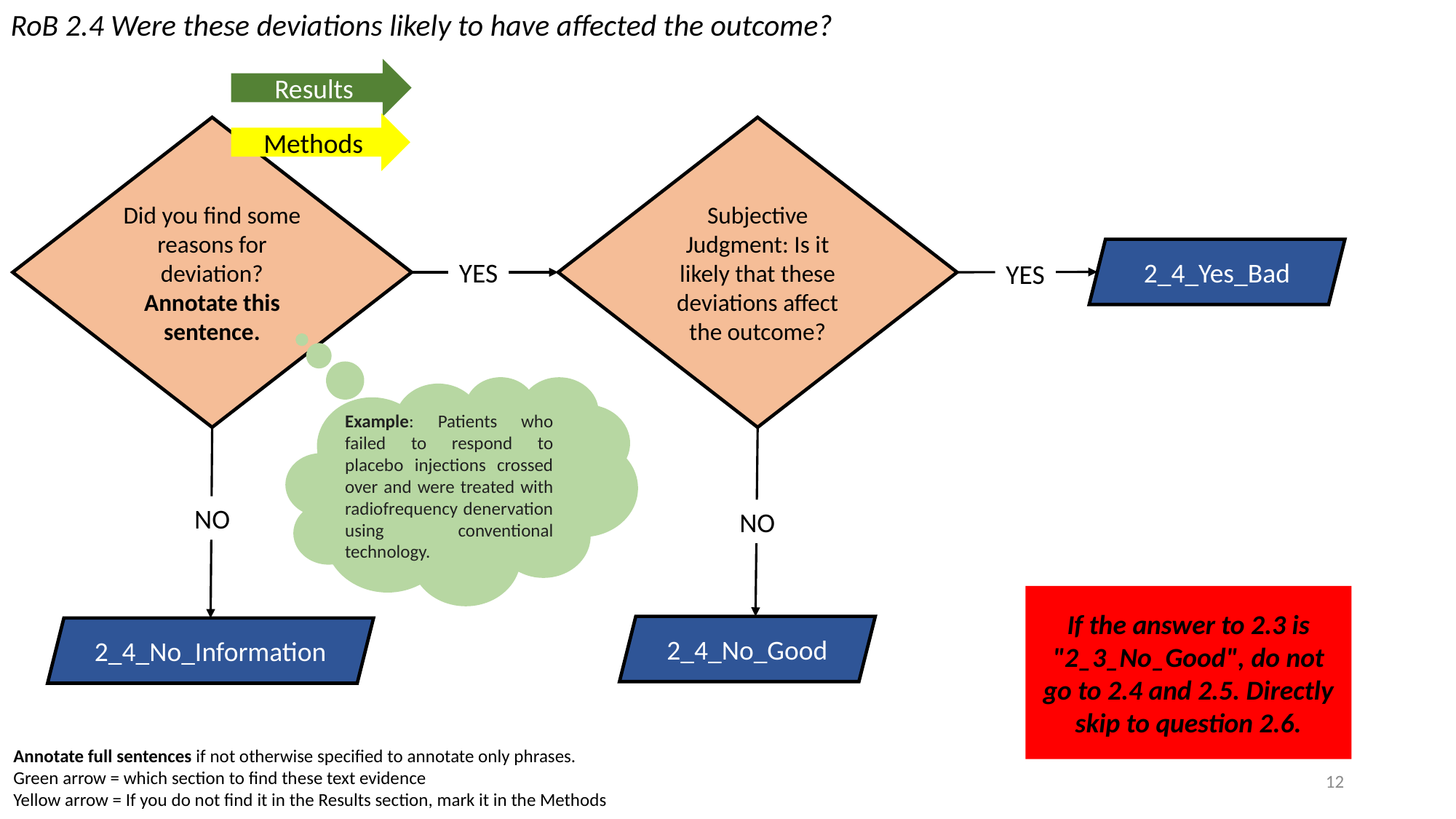

RoB 2.4 Were these deviations likely to have affected the outcome?
Results
Methods
Did you find some reasons for deviation? Annotate this sentence.
Subjective Judgment: Is it likely that these deviations affect the outcome?
2_4_Yes_Bad
YES
YES
Example: Patients who failed to respond to placebo injections crossed over and were treated with radiofrequency denervation using conventional technology.
NO
NO
If the answer to 2.3 is "2_3_No_Good", do not go to 2.4 and 2.5. Directly skip to question 2.6.
2_4_No_Good
2_4_No_Information
Annotate full sentences if not otherwise specified to annotate only phrases.
Green arrow = which section to find these text evidence
Yellow arrow = If you do not find it in the Results section, mark it in the Methods
12

## Slide 13
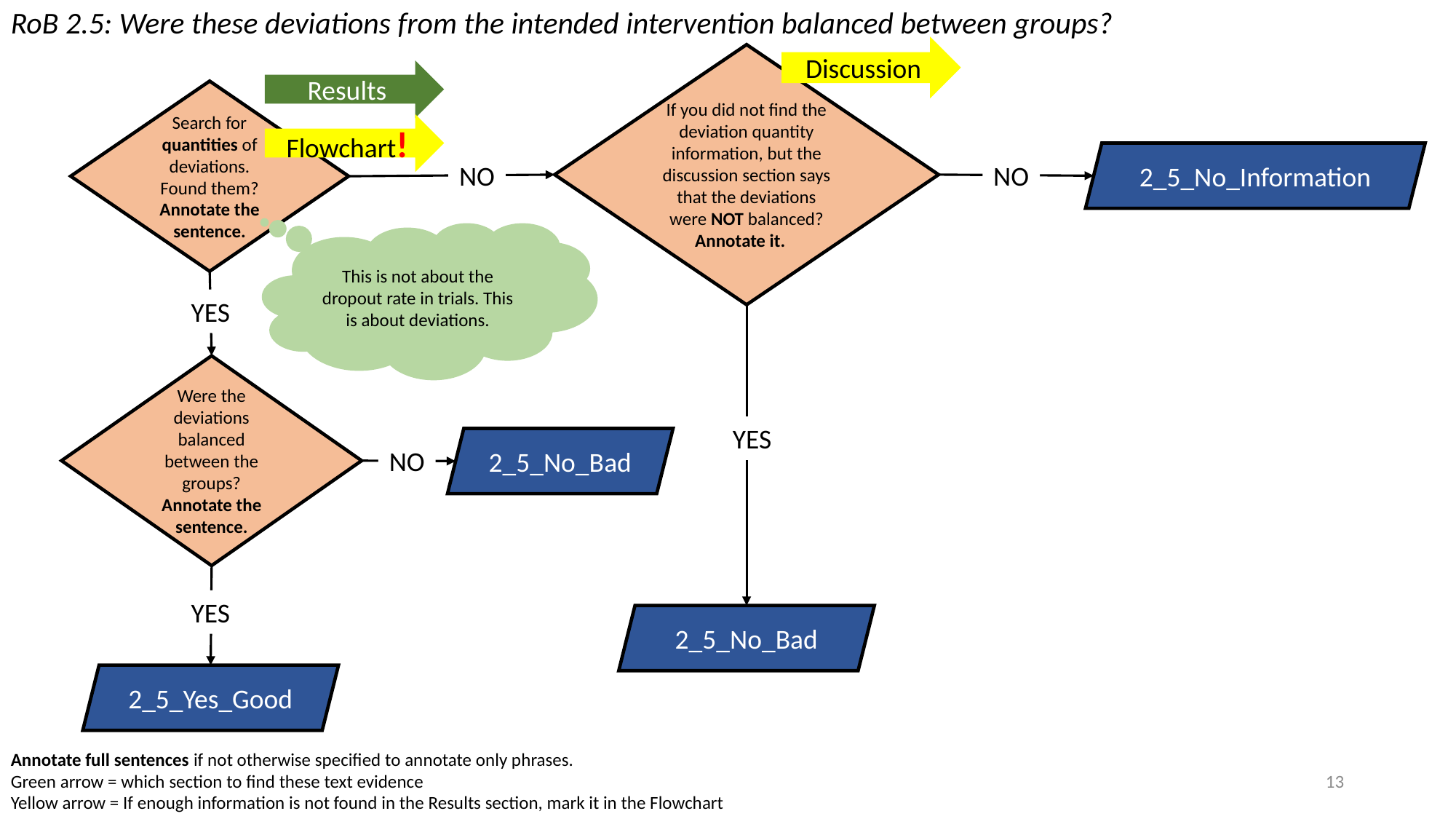

RoB 2.5: Were these deviations from the intended intervention balanced between groups?
Discussion
If you did not find the deviation quantity information, but the discussion section says that the deviations were NOT balanced? Annotate it.
Results
Search for quantities of deviations. Found them?
Annotate the sentence.
Flowchart!
2_5_No_Information
NO
NO
This is not about the dropout rate in trials. This is about deviations.
YES
Were the deviations balanced between the groups? Annotate the sentence.
YES
2_5_No_Bad
NO
YES
2_5_No_Bad
2_5_Yes_Good
Annotate full sentences if not otherwise specified to annotate only phrases.
Green arrow = which section to find these text evidence
Yellow arrow = If enough information is not found in the Results section, mark it in the Flowchart
13

## Slide 14
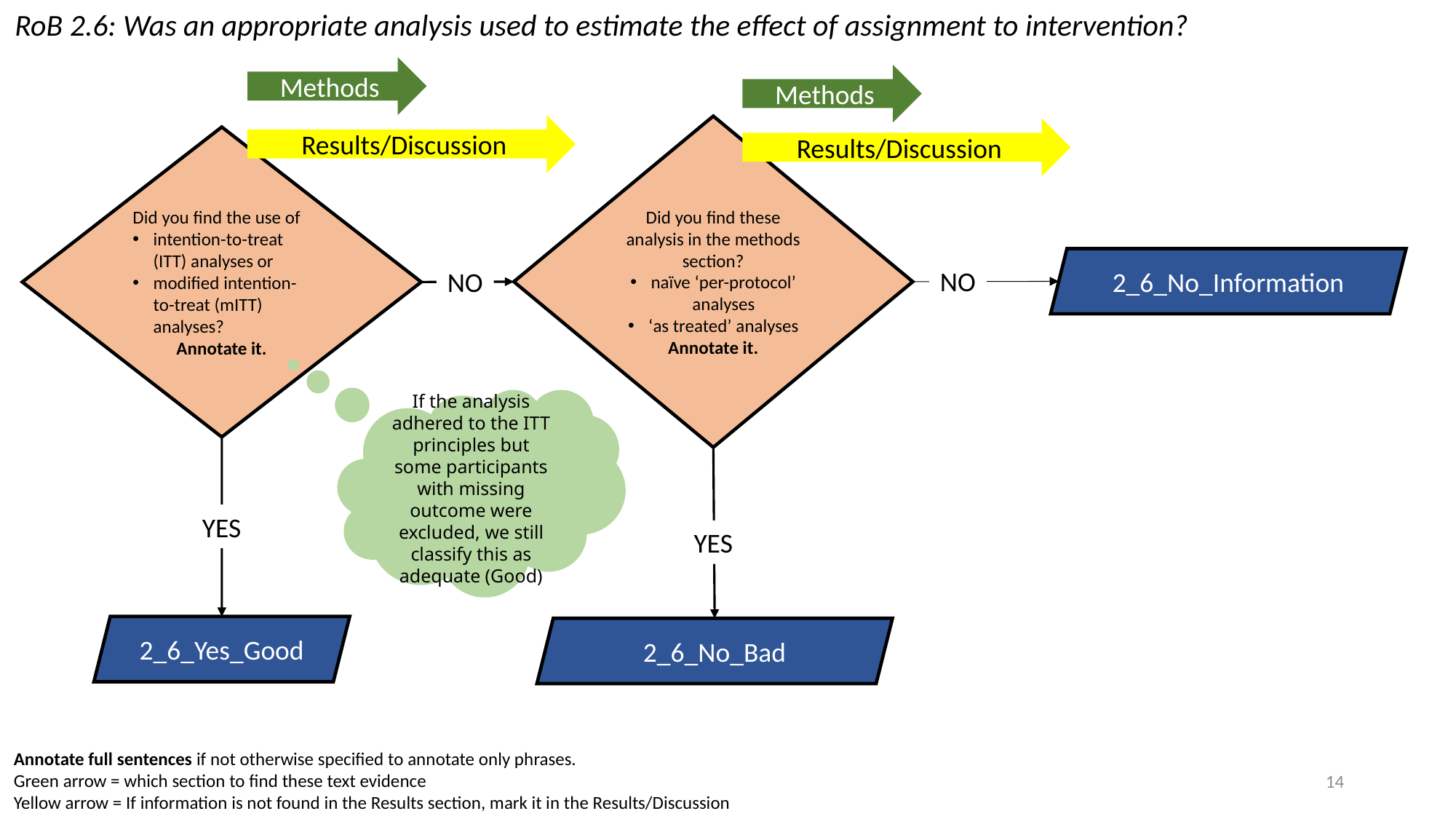

RoB 2.6: Was an appropriate analysis used to estimate the effect of assignment to intervention?
Methods
Methods
Results/Discussion
Did you find these analysis in the methods section?
naïve ‘per-protocol’ analyses
‘as treated’ analyses
Annotate it.
Results/Discussion
Did you find the use of
intention-to-treat (ITT) analyses or
modified intention-to-treat (mITT) analyses?
Annotate it.
2_6_No_Information
NO
NO
If the analysis adhered to the ITT principles but some participants with missing outcome were excluded, we still classify this as adequate (Good)
YES
YES
2_6_Yes_Good
2_6_No_Bad
Annotate full sentences if not otherwise specified to annotate only phrases.
Green arrow = which section to find these text evidence
Yellow arrow = If information is not found in the Results section, mark it in the Results/Discussion
14

## Slide 15
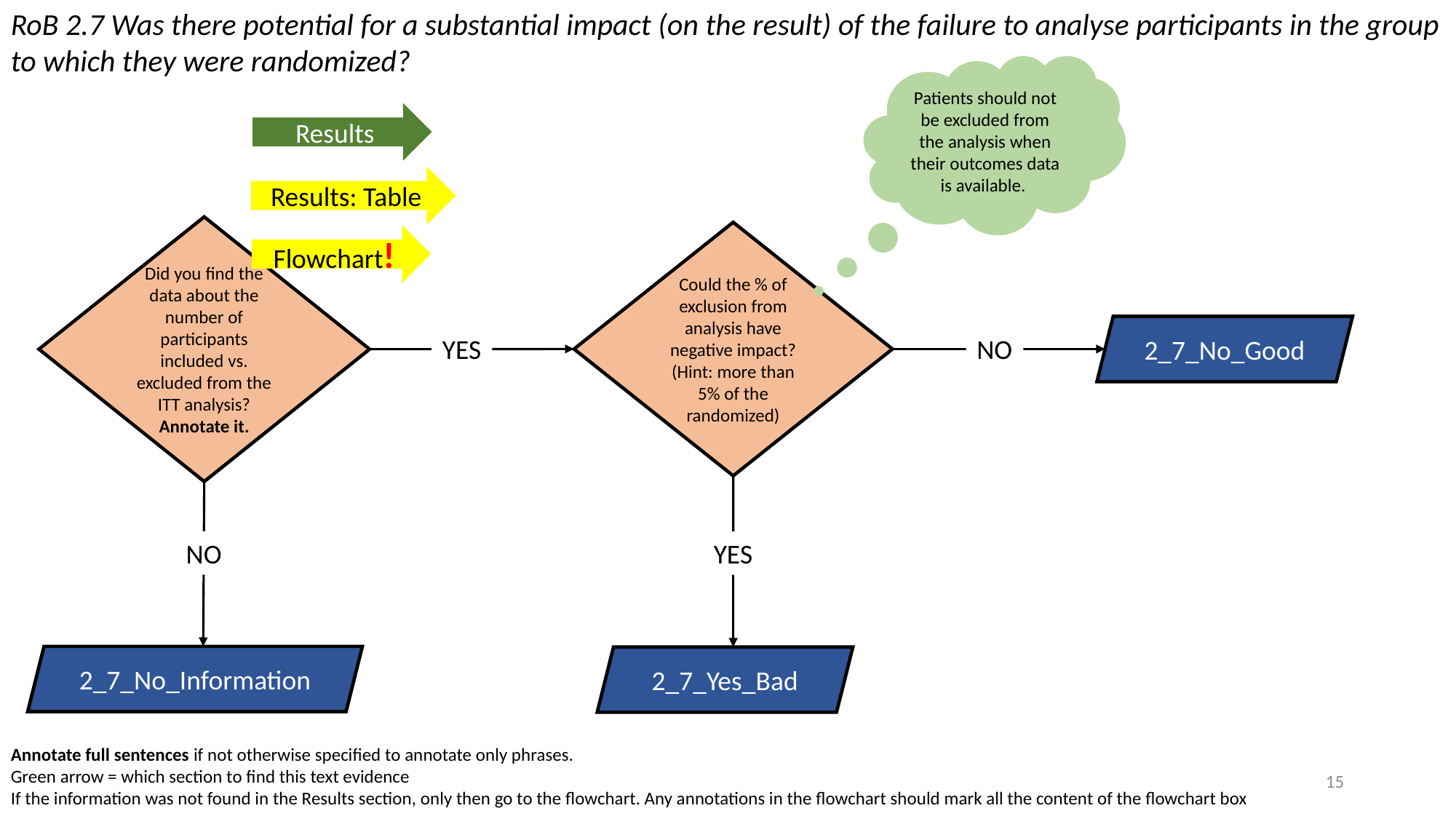

RoB 2.7 Was there potential for a substantial impact (on the result) of the failure to analyse participants in the group to which they were randomized?
Patients should not be excluded from the analysis when their outcomes data is available.
Results
Results: Table
Did you find the data about the number of participants included vs. excluded from the ITT analysis? Annotate it.
Could the % of exclusion from analysis have negative impact? (Hint: more than 5% of the randomized)
Flowchart!
2_7_No_Good
YES
NO
NO
YES
2_7_No_Information
2_7_Yes_Bad
Annotate full sentences if not otherwise specified to annotate only phrases.
Green arrow = which section to find this text evidence
If the information was not found in the Results section, only then go to the flowchart. Any annotations in the flowchart should mark all the content of the flowchart box
15

## Slide 16
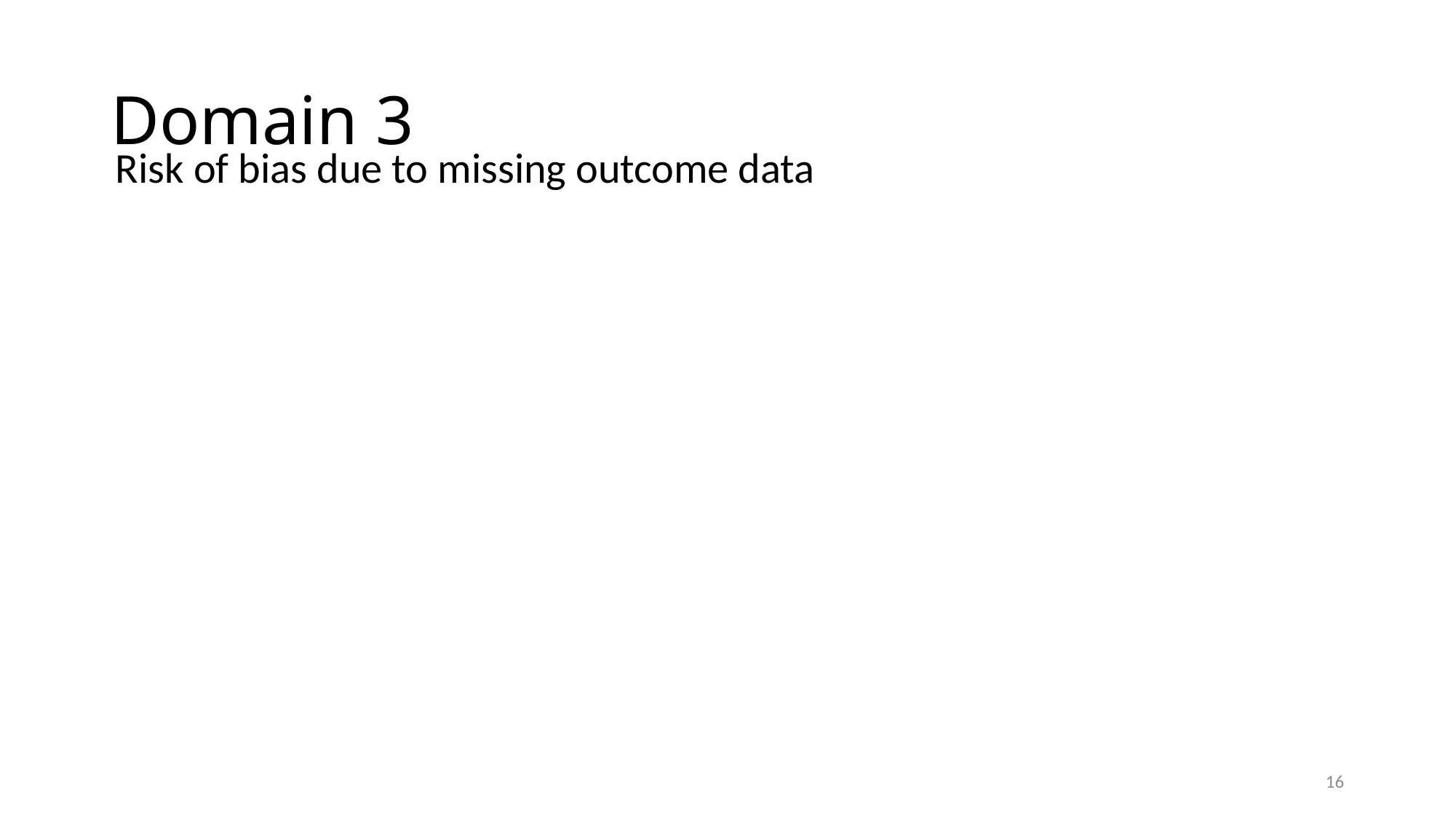

# Domain 3
Risk of bias due to missing outcome data
16

## Slide 17
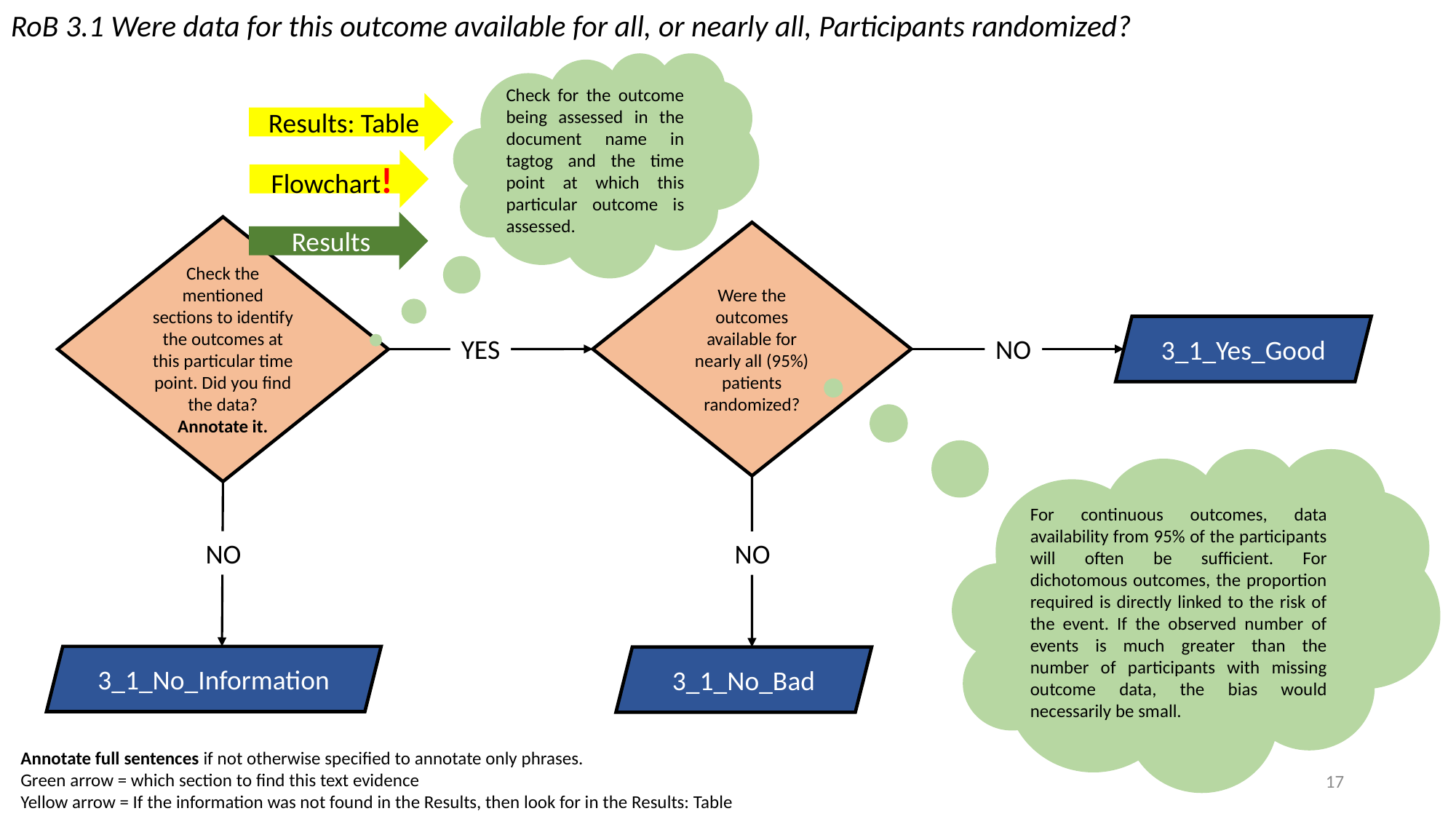

RoB 3.1 Were data for this outcome available for all, or nearly all, Participants randomized?
Check for the outcome being assessed in the document name in tagtog and the time point at which this particular outcome is assessed.
Results: Table
Flowchart!
Results
Check the mentioned sections to identify the outcomes at this particular time point. Did you find the data? Annotate it.
Were the outcomes available for nearly all (95%) patients randomized?
3_1_Yes_Good
YES
NO
For continuous outcomes, data availability from 95% of the participants will often be sufficient. For dichotomous outcomes, the proportion required is directly linked to the risk of the event. If the observed number of events is much greater than the number of participants with missing outcome data, the bias would necessarily be small.
NO
NO
3_1_No_Information
3_1_No_Bad
Annotate full sentences if not otherwise specified to annotate only phrases.
Green arrow = which section to find this text evidence
Yellow arrow = If the information was not found in the Results, then look for in the Results: Table
17

## Slide 18
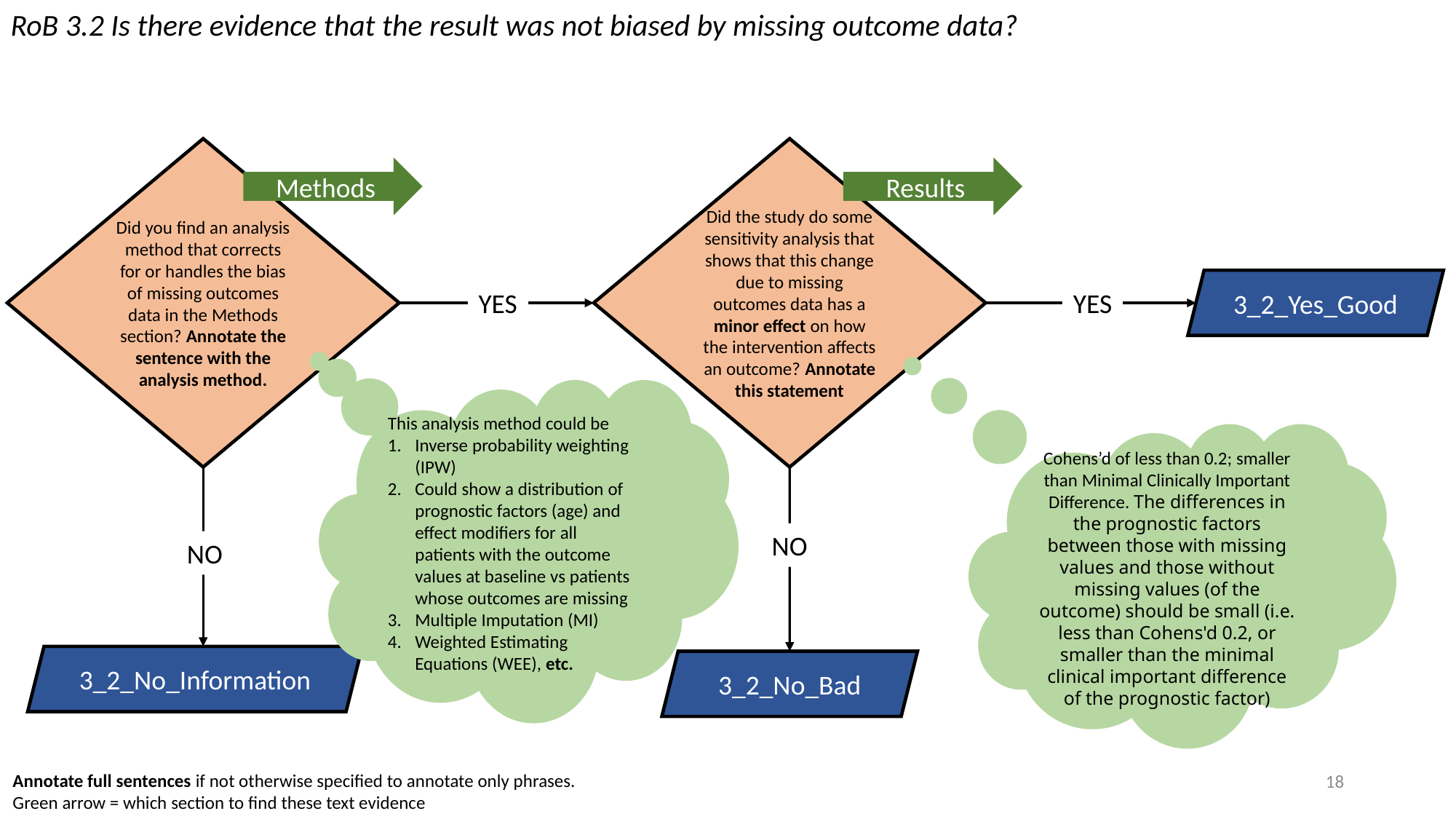

RoB 3.2 Is there evidence that the result was not biased by missing outcome data?
Did you find an analysis method that corrects for or handles the bias of missing outcomes data in the Methods section? Annotate the sentence with the analysis method.
Did the study do some sensitivity analysis that shows that this change due to missing outcomes data has a minor effect on how the intervention affects an outcome? Annotate this statement
Methods
Results
3_2_Yes_Good
YES
YES
This analysis method could be
Inverse probability weighting (IPW)
Could show a distribution of prognostic factors (age) and effect modifiers for all patients with the outcome values at baseline vs patients whose outcomes are missing
Multiple Imputation (MI)
Weighted Estimating Equations (WEE), etc.
Cohens’d of less than 0.2; smaller than Minimal Clinically Important Difference. The differences in the prognostic factors between those with missing values and those without missing values (of the outcome) should be small (i.e. less than Cohens'd 0.2, or smaller than the minimal clinical important difference of the prognostic factor)
NO
NO
3_2_No_Information
3_2_No_Bad
18
Annotate full sentences if not otherwise specified to annotate only phrases.
Green arrow = which section to find these text evidence

## Slide 19
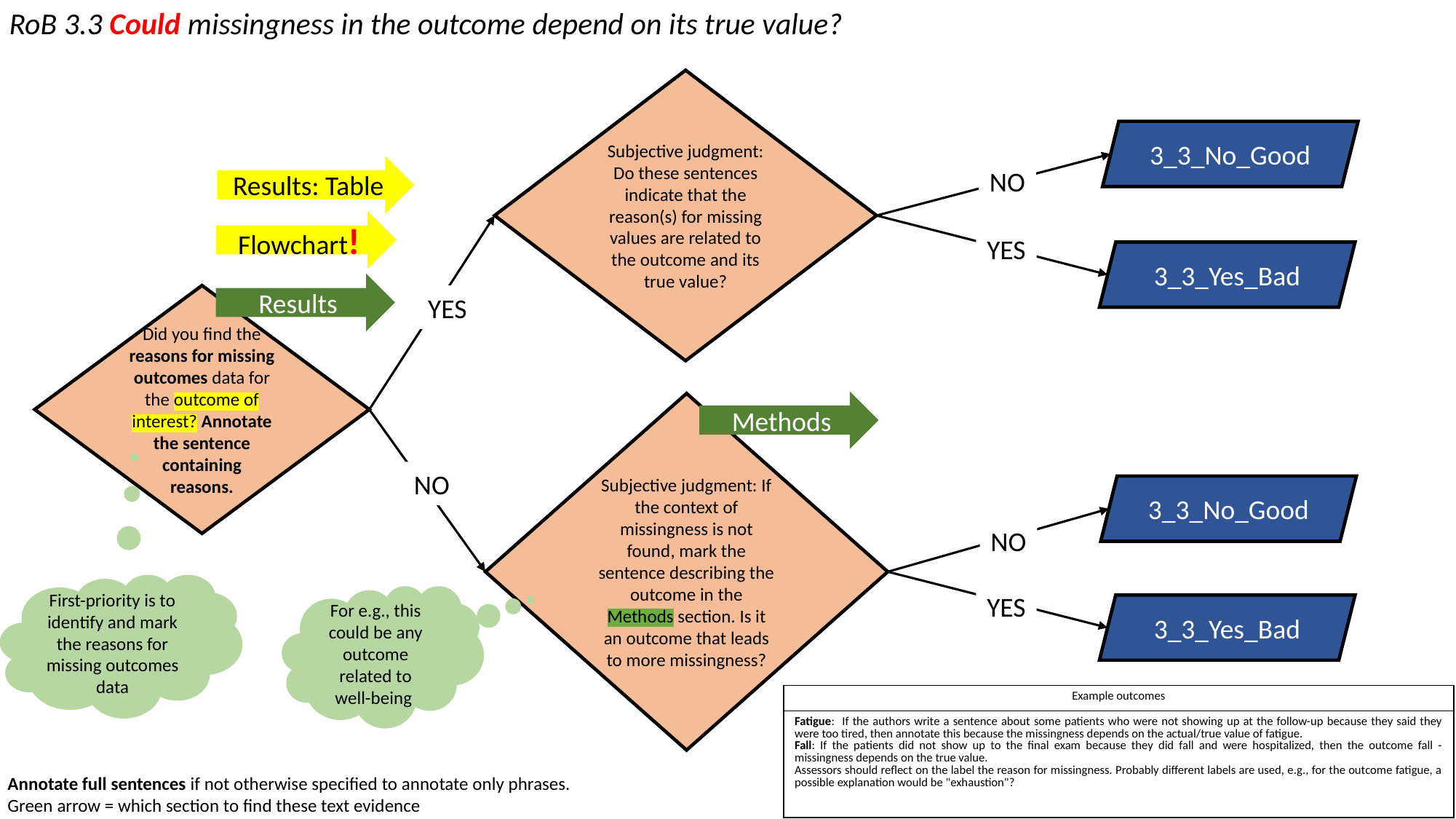

RoB 3.3 Could missingness in the outcome depend on its true value?
Subjective judgment: Do these sentences indicate that the reason(s) for missing values are related to the outcome and its true value?
3_3_No_Good
Results: Table
NO
Flowchart!
YES
3_3_Yes_Bad
Results
Did you find the reasons for missing outcomes data for the outcome of interest? Annotate the sentence containing reasons.
YES
Methods
Subjective judgment: If the context of missingness is not found, mark the sentence describing the outcome in the Methods section. Is it an outcome that leads to more missingness?
NO
3_3_No_Good
NO
First-priority is to identify and mark the reasons for missing outcomes data
YES
For e.g., this could be any outcome related to well-being
3_3_Yes_Bad
| Example outcomes |
| --- |
| Fatigue: If the authors write a sentence about some patients who were not showing up at the follow-up because they said they were too tired, then annotate this because the missingness depends on the actual/true value of fatigue. Fall: If the patients did not show up to the final exam because they did fall and were hospitalized, then the outcome fall -missingness depends on the true value. Assessors should reflect on the label the reason for missingness. Probably different labels are used, e.g., for the outcome fatigue, a possible explanation would be "exhaustion"? |
Annotate full sentences if not otherwise specified to annotate only phrases.
Green arrow = which section to find these text evidence

## Slide 20
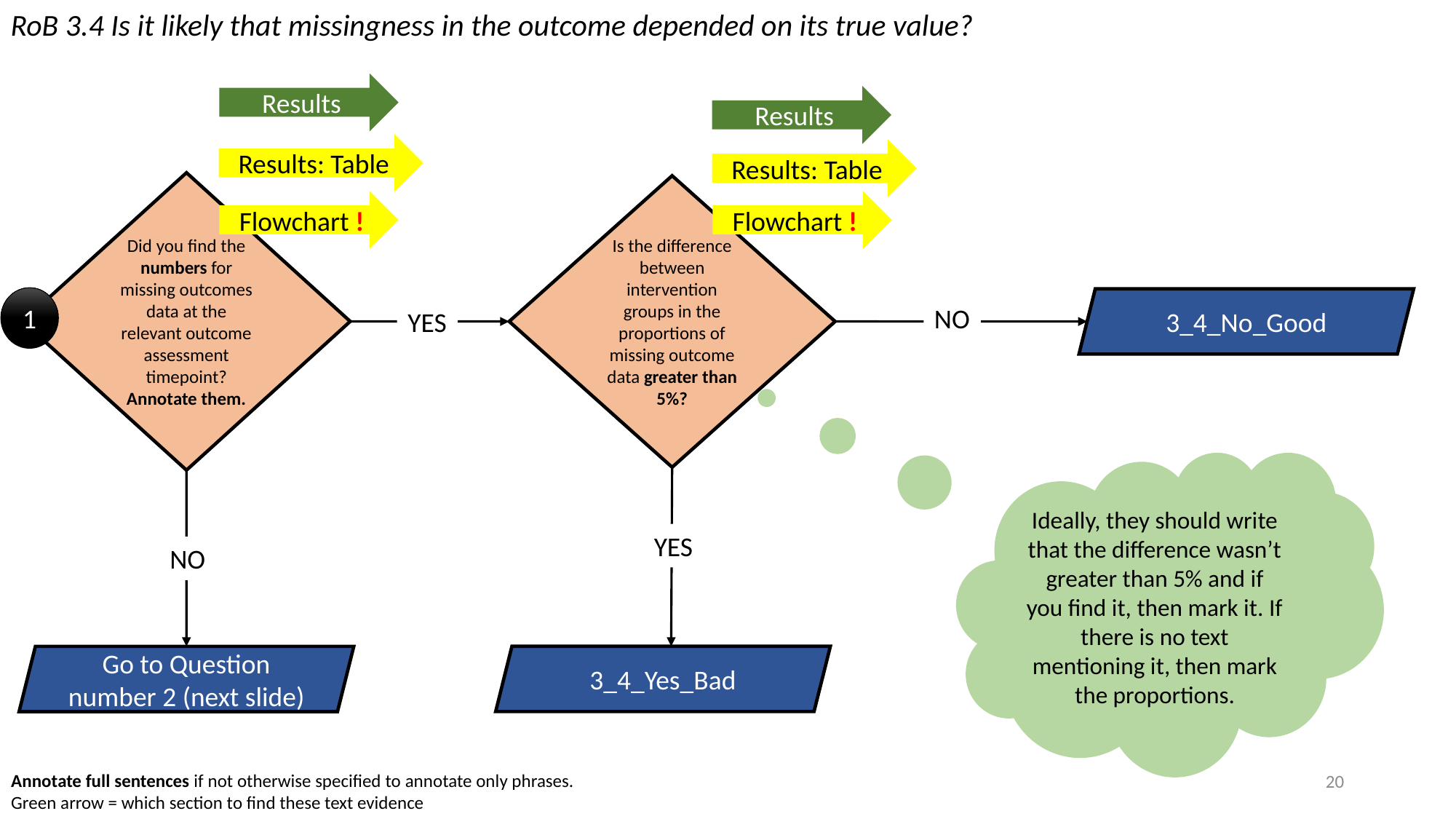

RoB 3.4 Is it likely that missingness in the outcome depended on its true value?
Results
Results
Results: Table
Results: Table
Did you find the numbers for missing outcomes data at the relevant outcome assessment timepoint? Annotate them.
Is the difference between intervention groups in the proportions of missing outcome data greater than 5%?
Flowchart !
Flowchart !
1
3_4_No_Good
NO
YES
Ideally, they should write that the difference wasn’t greater than 5% and if you find it, then mark it. If there is no text mentioning it, then mark the proportions.
YES
NO
3_4_Yes_Bad
Go to Question number 2 (next slide)
20
Annotate full sentences if not otherwise specified to annotate only phrases.
Green arrow = which section to find these text evidence

## Slide 21
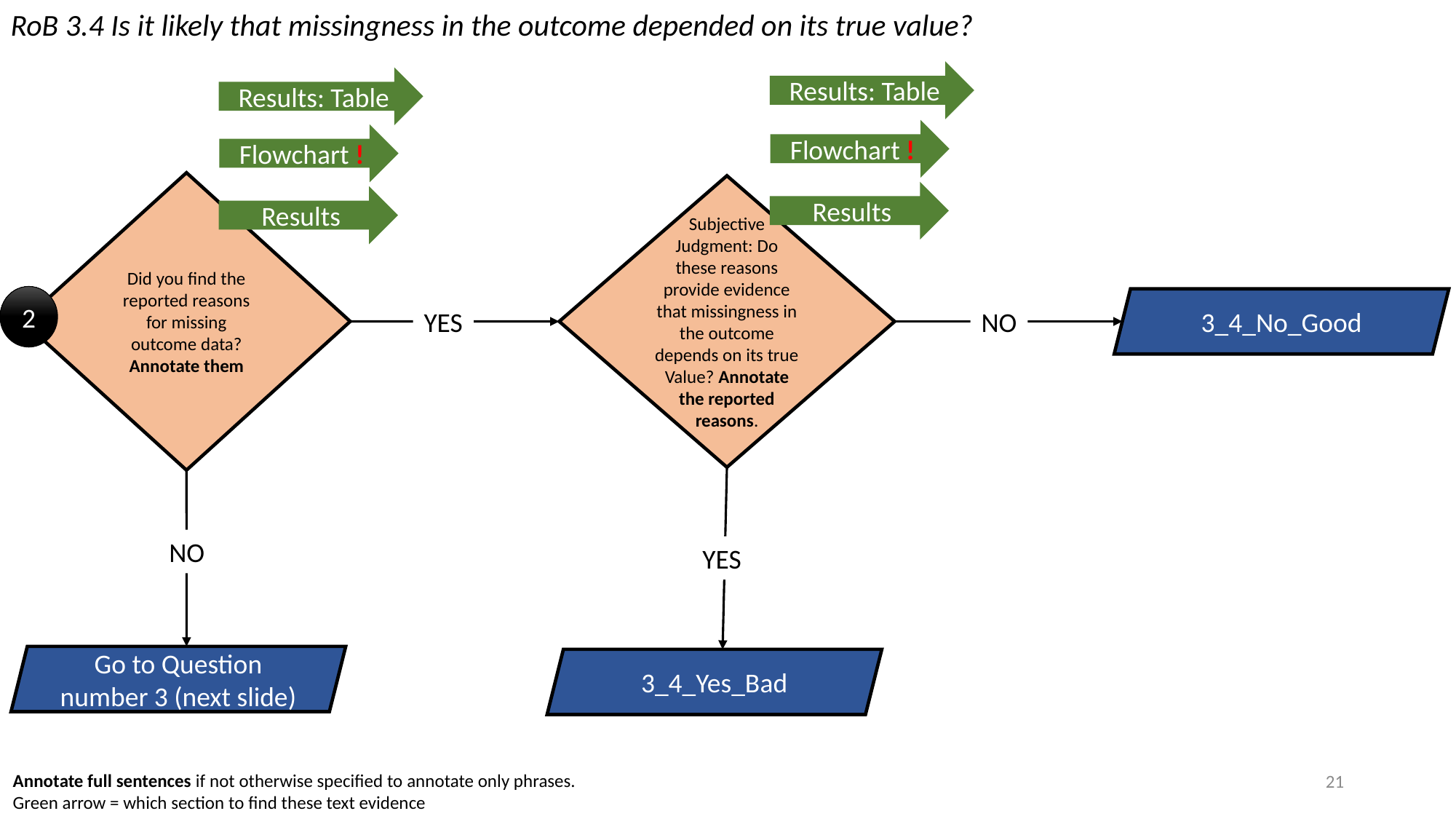

RoB 3.4 Is it likely that missingness in the outcome depended on its true value?
Results: Table
Results: Table
Flowchart !
Flowchart !
Did you find the reported reasons for missing outcome data? Annotate them
Subjective Judgment: Do these reasons provide evidence that missingness in the outcome depends on its true
Value? Annotate the reported reasons.
Results
Results
2
3_4_No_Good
YES
NO
NO
YES
Go to Question number 3 (next slide)
3_4_Yes_Bad
21
Annotate full sentences if not otherwise specified to annotate only phrases.
Green arrow = which section to find these text evidence

## Slide 22
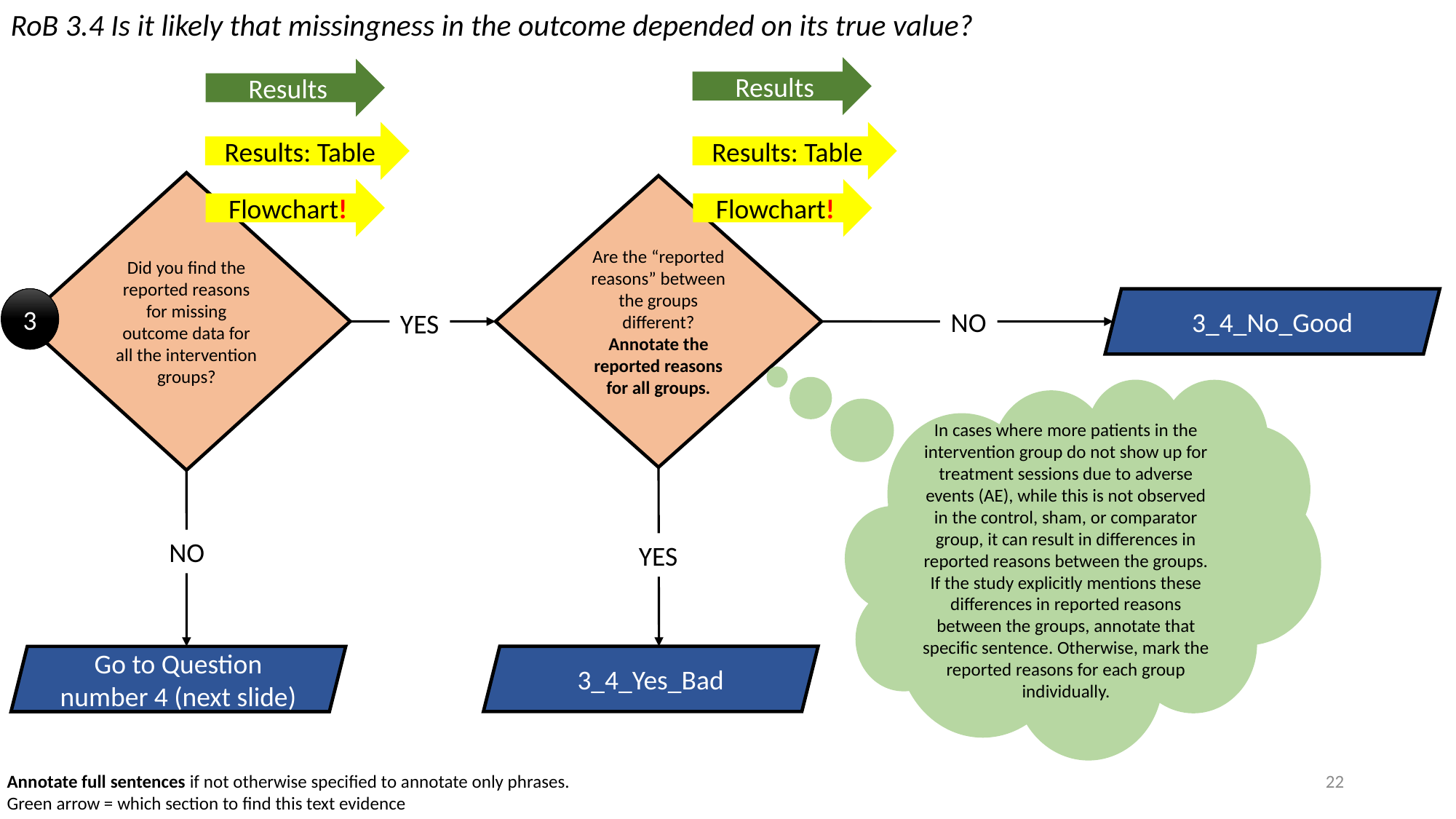

RoB 3.4 Is it likely that missingness in the outcome depended on its true value?
Results
Results
Results: Table
Results: Table
Did you find the reported reasons for missing outcome data for all the intervention groups?
Are the “reported reasons” between the groups different? Annotate the reported reasons for all groups.
Flowchart!
Flowchart!
3
3_4_No_Good
NO
YES
In cases where more patients in the intervention group do not show up for treatment sessions due to adverse events (AE), while this is not observed in the control, sham, or comparator group, it can result in differences in reported reasons between the groups. If the study explicitly mentions these differences in reported reasons between the groups, annotate that specific sentence. Otherwise, mark the reported reasons for each group individually.
NO
YES
3_4_Yes_Bad
Go to Question number 4 (next slide)
22
Annotate full sentences if not otherwise specified to annotate only phrases.
Green arrow = which section to find this text evidence

## Slide 23
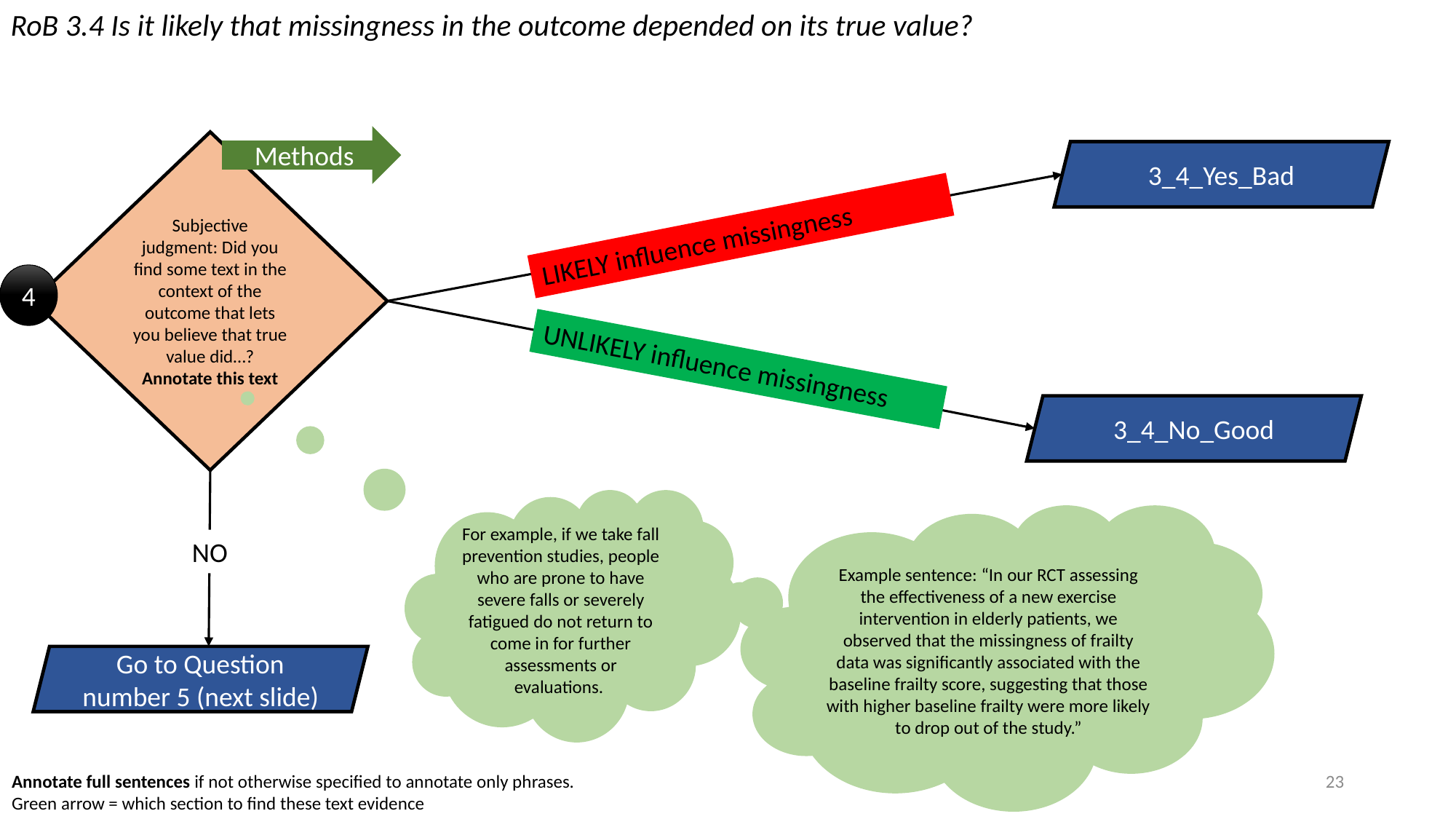

RoB 3.4 Is it likely that missingness in the outcome depended on its true value?
Methods
Subjective judgment: Did you find some text in the context of the outcome that lets you believe that true value did…? Annotate this text
3_4_Yes_Bad
LIKELY influence missingness
4
UNLIKELY influence missingness
3_4_No_Good
For example, if we take fall prevention studies, people who are prone to have severe falls or severely fatigued do not return to come in for further assessments or evaluations.
Example sentence: “In our RCT assessing the effectiveness of a new exercise intervention in elderly patients, we observed that the missingness of frailty data was significantly associated with the baseline frailty score, suggesting that those with higher baseline frailty were more likely to drop out of the study.”
NO
Go to Question number 5 (next slide)
23
Annotate full sentences if not otherwise specified to annotate only phrases.
Green arrow = which section to find these text evidence

## Slide 24
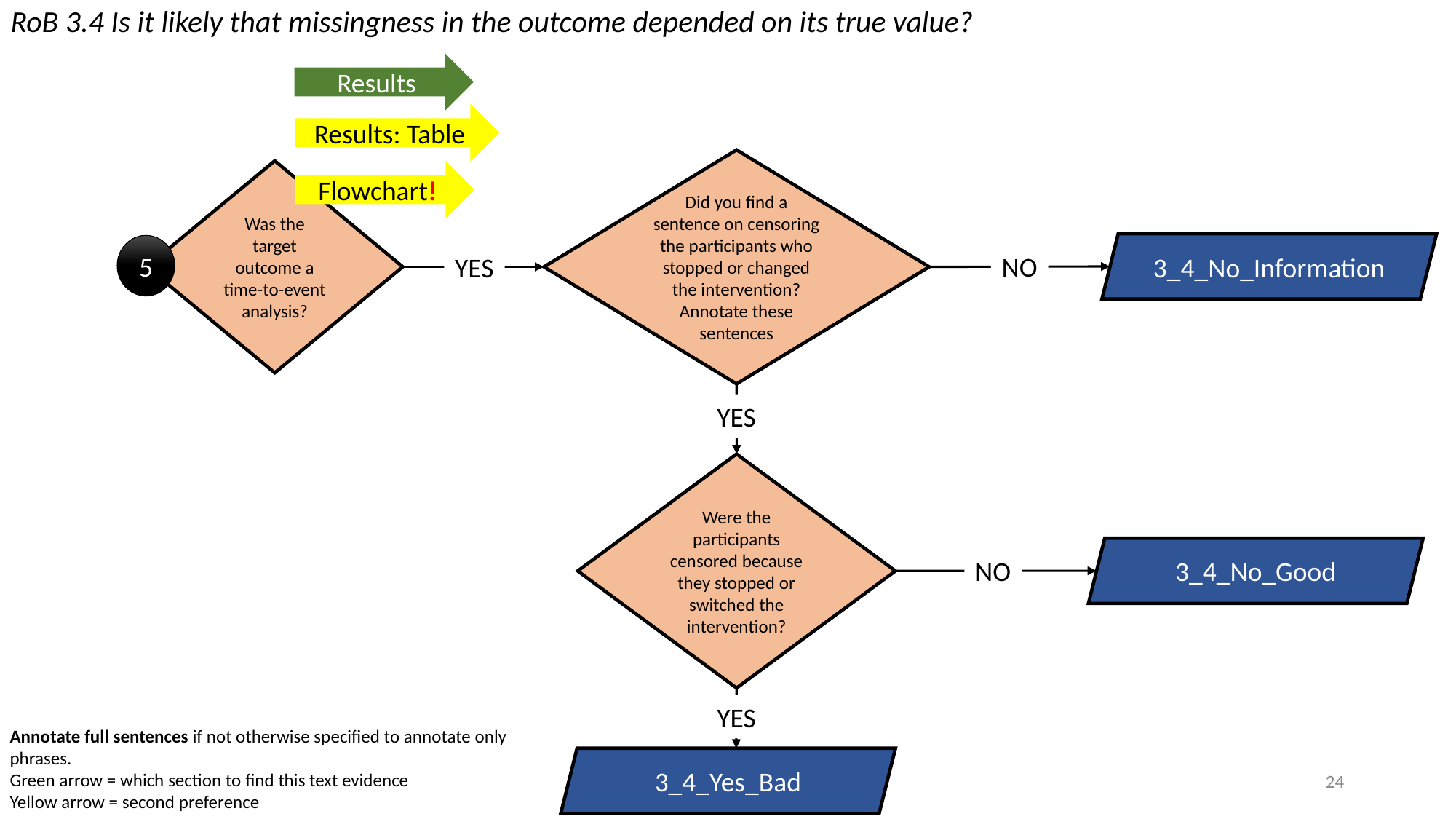

RoB 3.4 Is it likely that missingness in the outcome depended on its true value?
Results
Results: Table
Did you find a sentence on censoring the participants who stopped or changed the intervention? Annotate these sentences
Flowchart!
Was the target outcome a time-to-event analysis?
3_4_No_Information
5
NO
YES
YES
Were the participants censored because they stopped or switched the intervention?
3_4_No_Good
NO
YES
Annotate full sentences if not otherwise specified to annotate only phrases.
Green arrow = which section to find this text evidence
Yellow arrow = second preference
3_4_Yes_Bad
24

## Slide 25
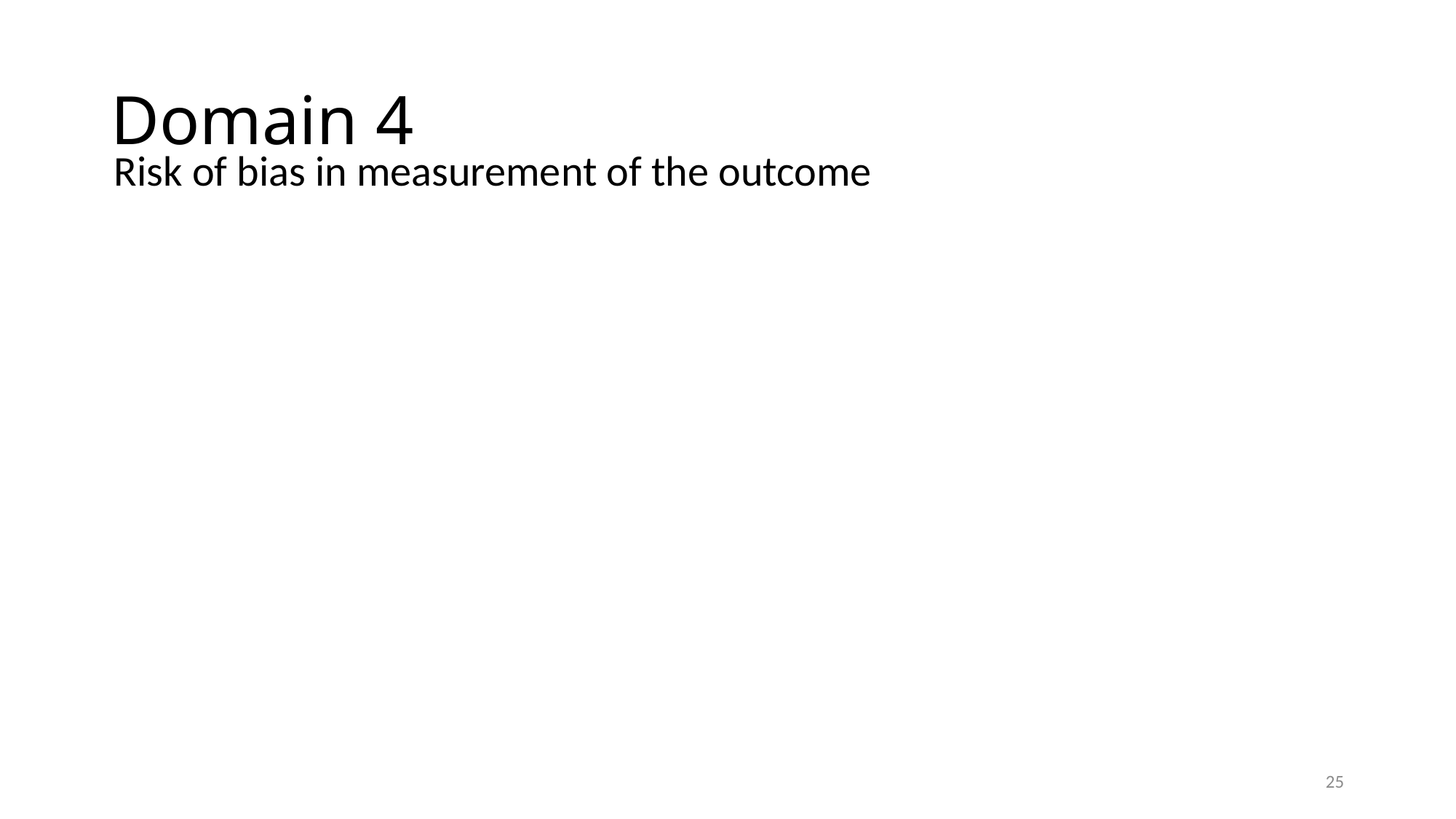

# Domain 4
Risk of bias in measurement of the outcome
25

## Slide 26
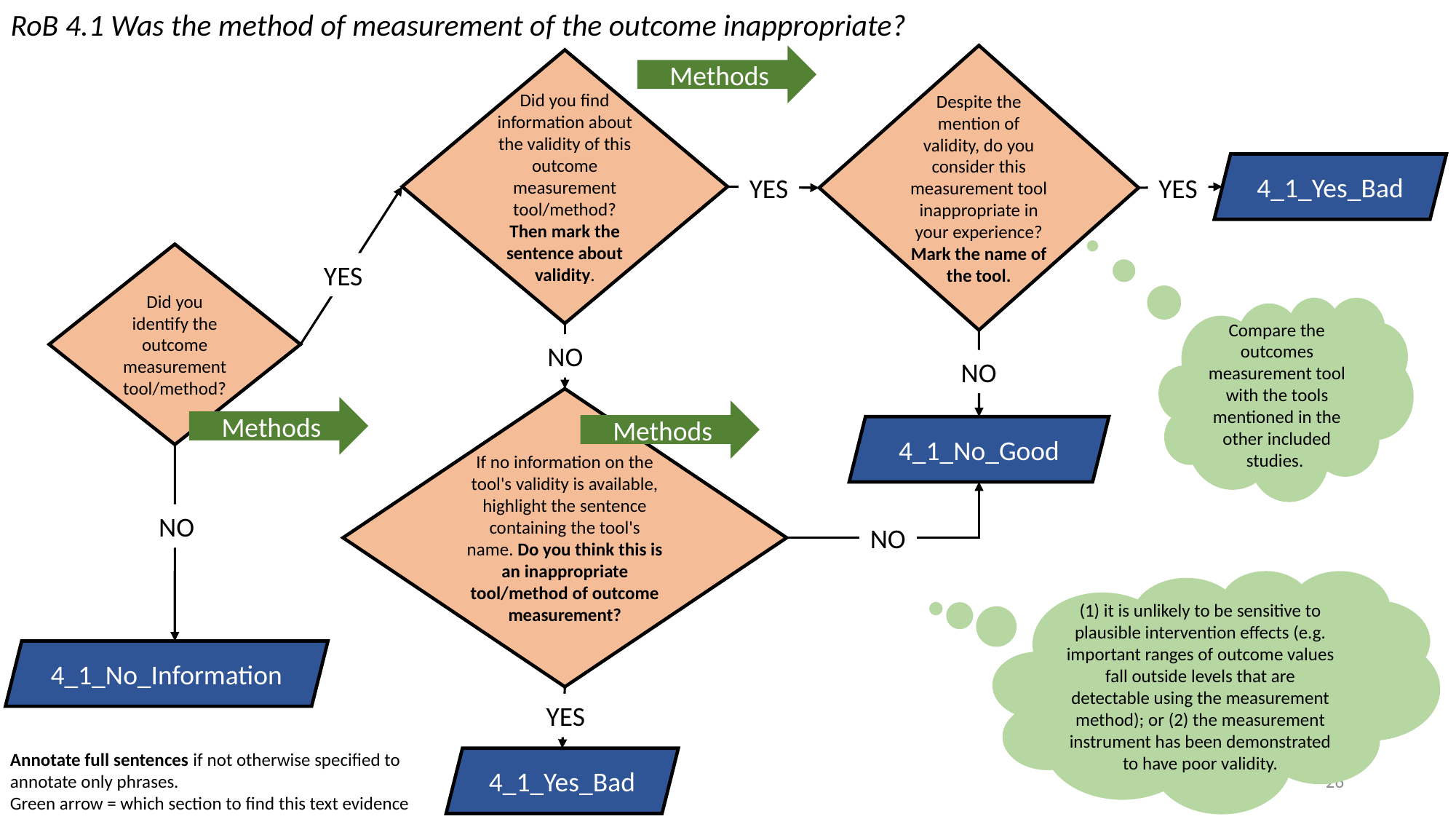

RoB 4.1 Was the method of measurement of the outcome inappropriate?
Methods
Despite the mention of validity, do you consider this measurement tool inappropriate in your experience? Mark the name of the tool.
Did you find information about the validity of this outcome measurement tool/method? Then mark the sentence about validity.
4_1_Yes_Bad
YES
YES
Did you identify the outcome measurement tool/method?
YES
Compare the outcomes measurement tool with the tools mentioned in the other included studies.
NO
NO
If no information on the tool's validity is available, highlight the sentence containing the tool's name. Do you think this is an inappropriate tool/method of outcome measurement?
Methods
Methods
4_1_No_Good
NO
NO
(1) it is unlikely to be sensitive to plausible intervention effects (e.g. important ranges of outcome values fall outside levels that are detectable using the measurement method); or (2) the measurement instrument has been demonstrated to have poor validity.
4_1_No_Information
YES
Annotate full sentences if not otherwise specified to annotate only phrases.
Green arrow = which section to find this text evidence
4_1_Yes_Bad
26

## Slide 27
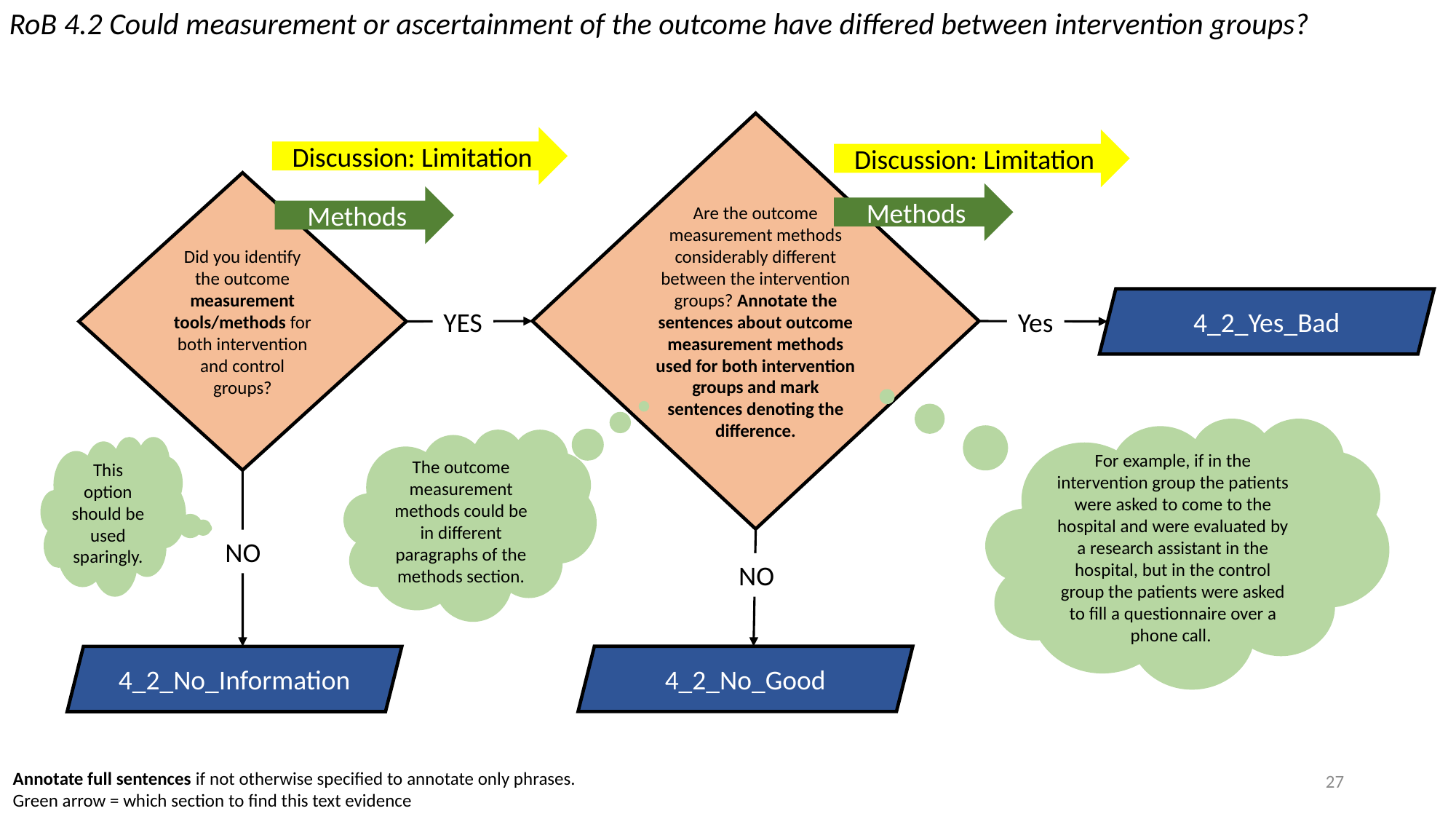

RoB 4.2 Could measurement or ascertainment of the outcome have differed between intervention groups?
Are the outcome measurement methods considerably different between the intervention groups? Annotate the sentences about outcome measurement methods used for both intervention groups and mark sentences denoting the difference.
Discussion: Limitation
Discussion: Limitation
Did you identify the outcome measurement tools/methods for both intervention and control groups?
Methods
Methods
4_2_Yes_Bad
YES
Yes
For example, if in the intervention group the patients were asked to come to the hospital and were evaluated by a research assistant in the hospital, but in the control group the patients were asked to fill a questionnaire over a phone call.
The outcome measurement methods could be in different paragraphs of the methods section.
This option should be used sparingly.
NO
NO
4_2_No_Good
4_2_No_Information
27
Annotate full sentences if not otherwise specified to annotate only phrases.
Green arrow = which section to find this text evidence

## Slide 28
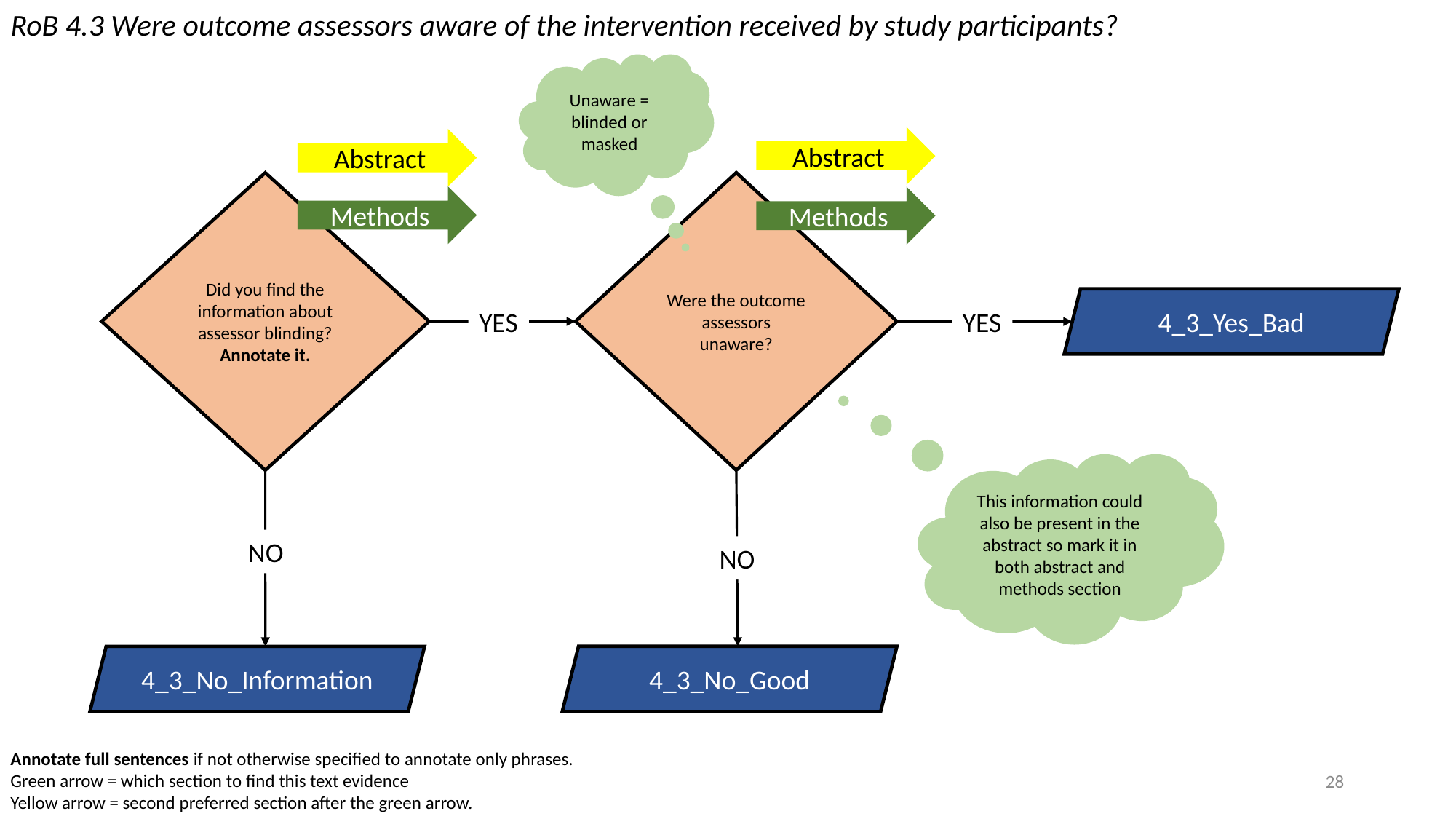

RoB 4.3 Were outcome assessors aware of the intervention received by study participants?
Unaware = blinded or masked
Abstract
Abstract
Were the outcome assessors unaware?
Did you find the information about assessor blinding? Annotate it.
Methods
Methods
4_3_Yes_Bad
YES
YES
This information could also be present in the abstract so mark it in both abstract and methods section
NO
NO
4_3_No_Good
4_3_No_Information
Annotate full sentences if not otherwise specified to annotate only phrases.
Green arrow = which section to find this text evidence
Yellow arrow = second preferred section after the green arrow.
28

## Slide 29
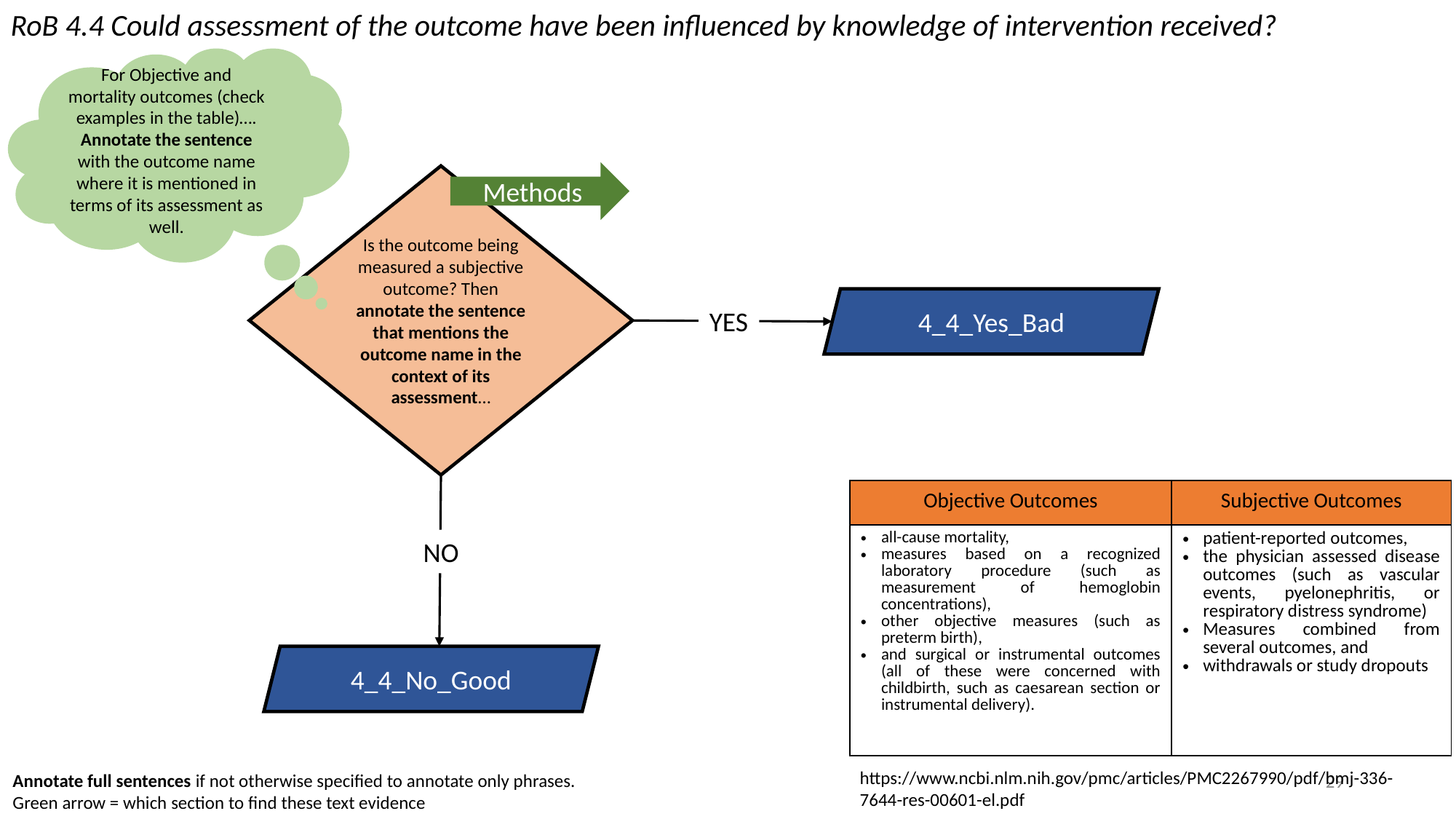

RoB 4.4 Could assessment of the outcome have been influenced by knowledge of intervention received?
For Objective and mortality outcomes (check examples in the table)…. Annotate the sentence with the outcome name where it is mentioned in terms of its assessment as well.
Methods
Is the outcome being measured a subjective outcome? Then annotate the sentence that mentions the outcome name in the context of its assessment…
4_4_Yes_Bad
YES
| Objective Outcomes | Subjective Outcomes |
| --- | --- |
| all-cause mortality, measures based on a recognized laboratory procedure (such as measurement of hemoglobin concentrations), other objective measures (such as preterm birth), and surgical or instrumental outcomes (all of these were concerned with childbirth, such as caesarean section or instrumental delivery). | patient-reported outcomes, the physician assessed disease outcomes (such as vascular events, pyelonephritis, or respiratory distress syndrome) Measures combined from several outcomes, and withdrawals or study dropouts |
NO
4_4_No_Good
29
https://www.ncbi.nlm.nih.gov/pmc/articles/PMC2267990/pdf/bmj-336-7644-res-00601-el.pdf
Annotate full sentences if not otherwise specified to annotate only phrases.
Green arrow = which section to find these text evidence

## Slide 30
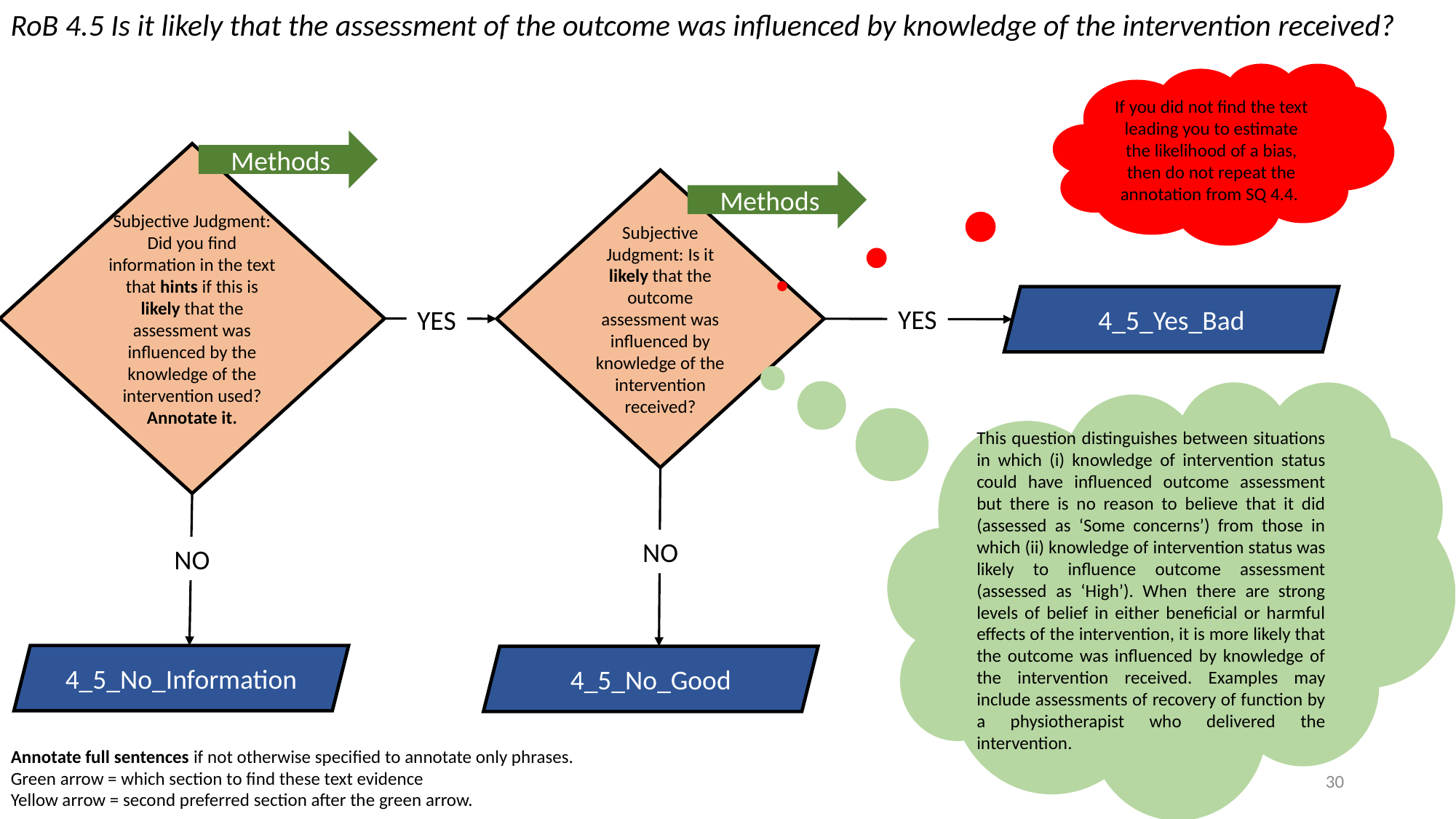

RoB 4.5 Is it likely that the assessment of the outcome was influenced by knowledge of the intervention received?
If you did not find the text leading you to estimate the likelihood of a bias, then do not repeat the annotation from SQ 4.4.
Methods
Subjective Judgment: Did you find information in the text that hints if this is likely that the assessment was influenced by the knowledge of the intervention used? Annotate it.
Subjective Judgment: Is it likely that the outcome assessment was influenced by knowledge of the intervention received?
Methods
4_5_Yes_Bad
YES
YES
This question distinguishes between situations in which (i) knowledge of intervention status could have influenced outcome assessment but there is no reason to believe that it did (assessed as ‘Some concerns’) from those in which (ii) knowledge of intervention status was likely to influence outcome assessment (assessed as ‘High’). When there are strong levels of belief in either beneficial or harmful effects of the intervention, it is more likely that the outcome was influenced by knowledge of the intervention received. Examples may include assessments of recovery of function by a physiotherapist who delivered the intervention.
NO
NO
4_5_No_Information
4_5_No_Good
Annotate full sentences if not otherwise specified to annotate only phrases.
Green arrow = which section to find these text evidence
Yellow arrow = second preferred section after the green arrow.
30

## Slide 31
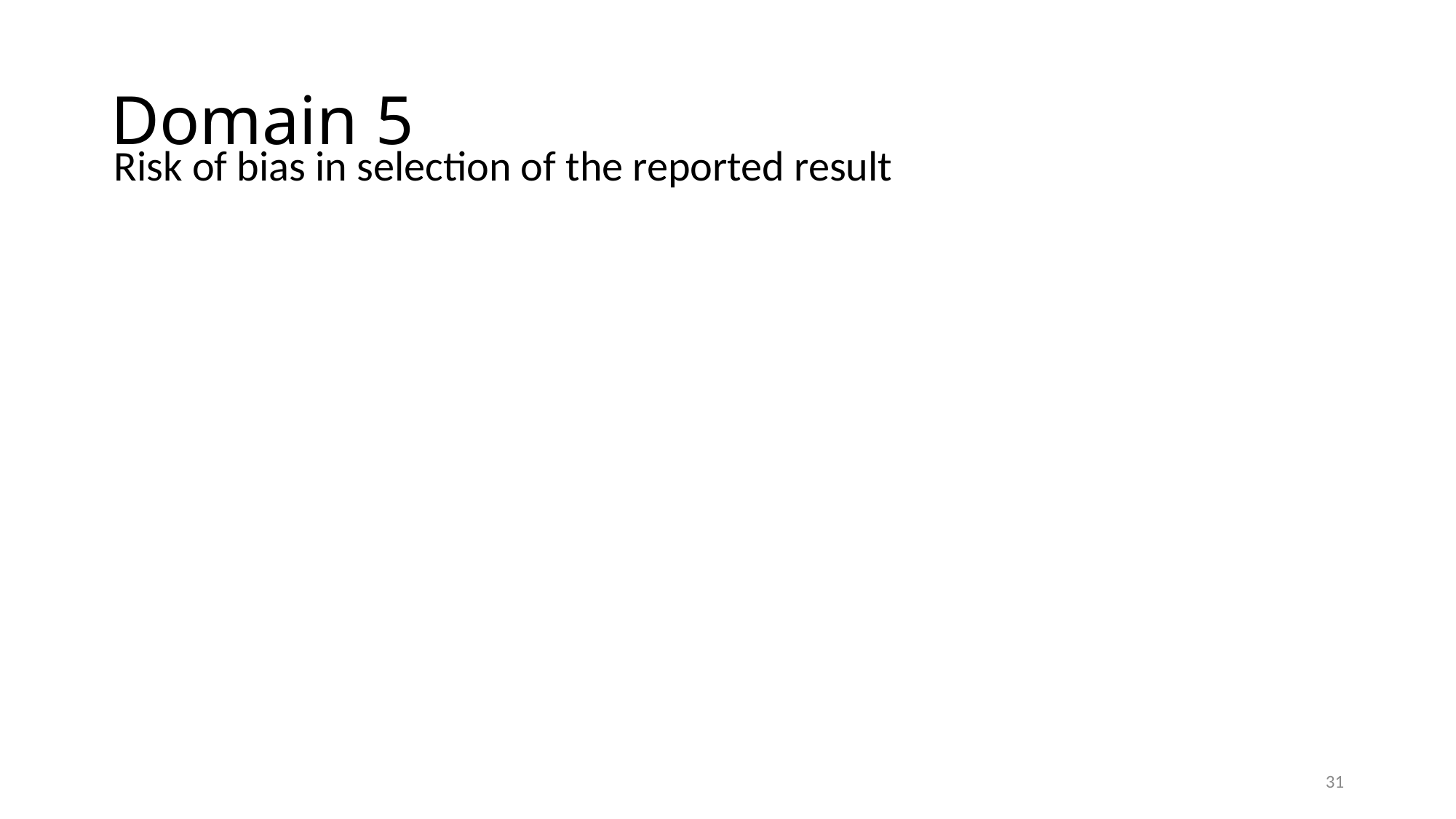

# Domain 5
Risk of bias in selection of the reported result
31

## Slide 32
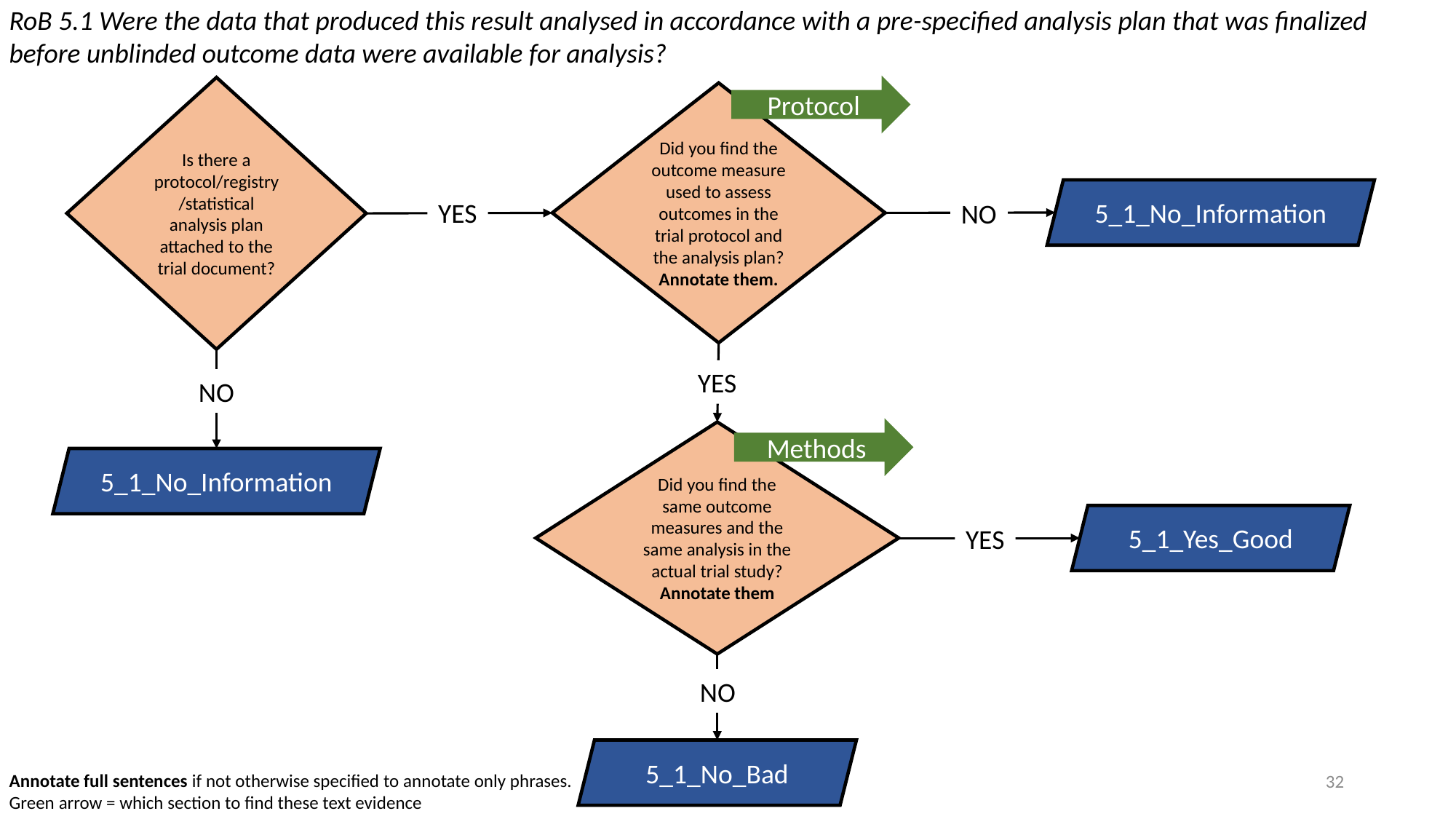

RoB 5.1 Were the data that produced this result analysed in accordance with a pre-specified analysis plan that was finalized before unblinded outcome data were available for analysis?
Protocol
Is there a protocol/registry/statistical analysis plan attached to the trial document?
Did you find the outcome measure used to assess outcomes in the trial protocol and the analysis plan? Annotate them.
5_1_No_Information
YES
NO
YES
NO
Methods
Did you find the same outcome measures and the same analysis in the actual trial study? Annotate them
5_1_No_Information
5_1_Yes_Good
YES
NO
5_1_No_Bad
32
Annotate full sentences if not otherwise specified to annotate only phrases.
Green arrow = which section to find these text evidence

## Slide 33
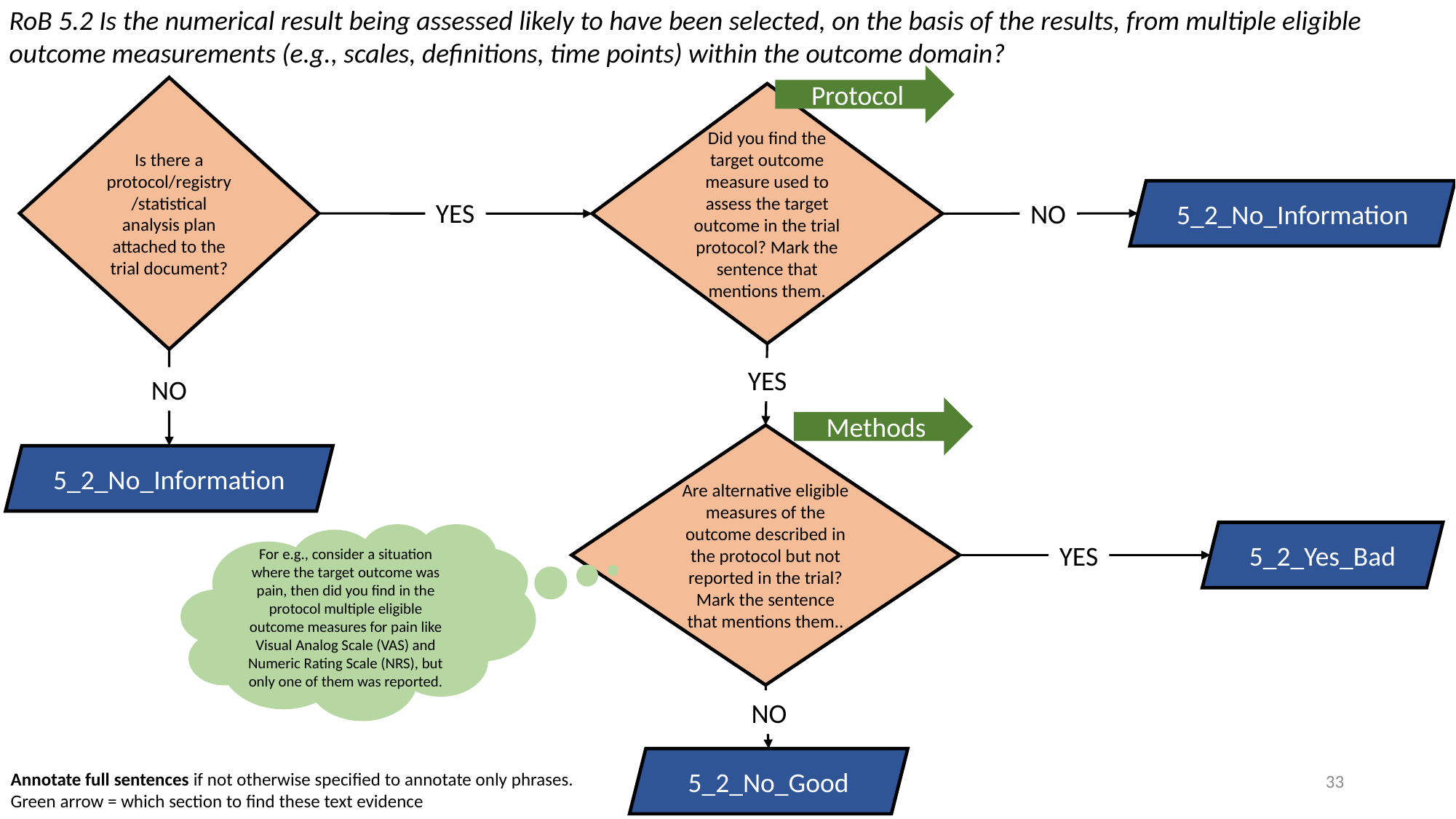

RoB 5.2 Is the numerical result being assessed likely to have been selected, on the basis of the results, from multiple eligible outcome measurements (e.g., scales, definitions, time points) within the outcome domain?
Protocol
Is there a protocol/registry/statistical analysis plan attached to the trial document?
Did you find the target outcome measure used to assess the target outcome in the trial protocol? Mark the sentence that mentions them.
5_2_No_Information
YES
NO
YES
NO
Methods
Are alternative eligible measures of the outcome described in the protocol but not reported in the trial? Mark the sentence that mentions them..
5_2_No_Information
5_2_Yes_Bad
For e.g., consider a situation where the target outcome was pain, then did you find in the protocol multiple eligible outcome measures for pain like Visual Analog Scale (VAS) and Numeric Rating Scale (NRS), but only one of them was reported.
YES
NO
5_2_No_Good
33
Annotate full sentences if not otherwise specified to annotate only phrases.
Green arrow = which section to find these text evidence

## Slide 34
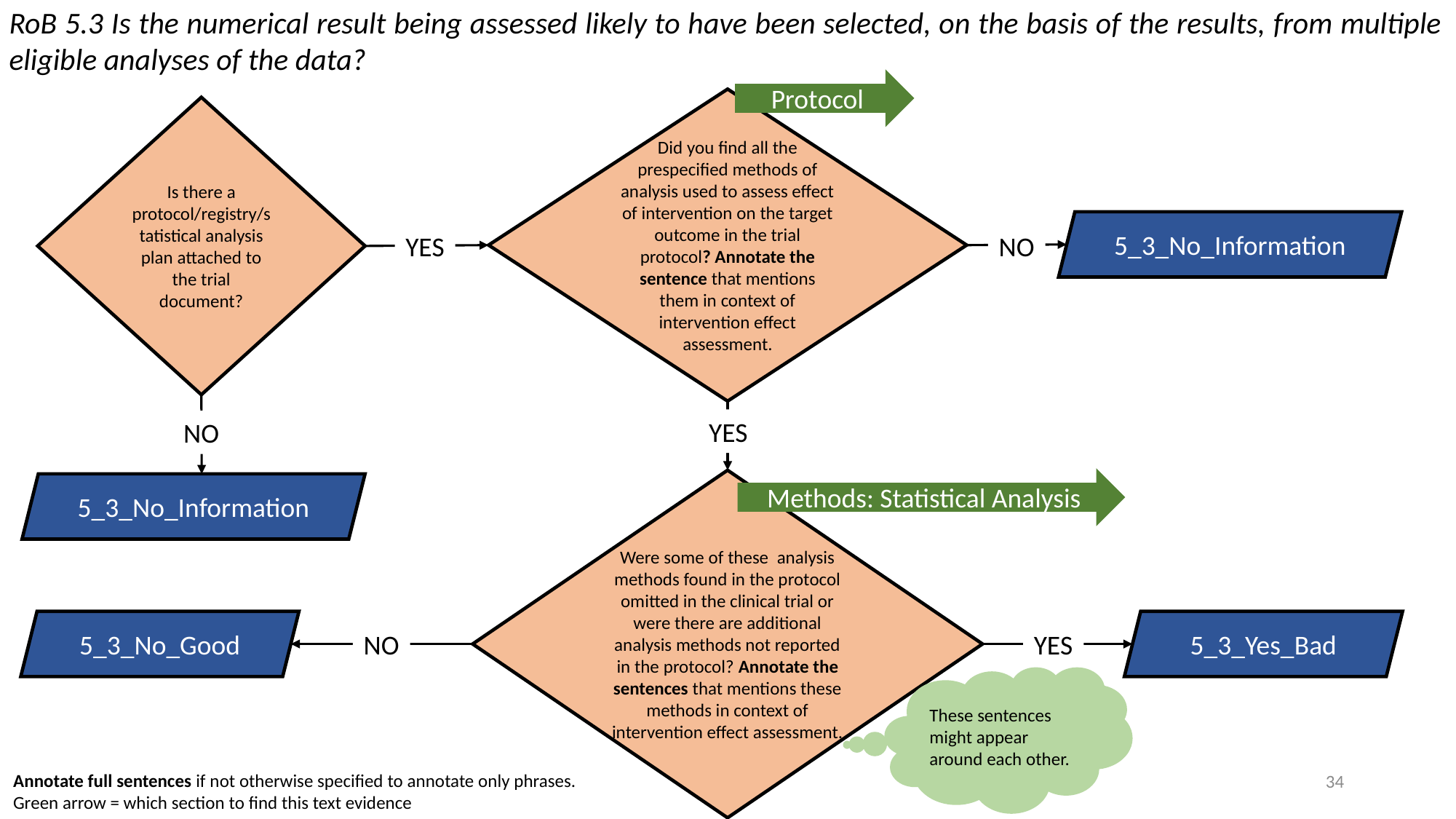

RoB 5.3 Is the numerical result being assessed likely to have been selected, on the basis of the results, from multiple eligible analyses of the data?
Protocol
Did you find all the prespecified methods of analysis used to assess effect of intervention on the target outcome in the trial protocol? Annotate the sentence that mentions them in context of intervention effect assessment.
Is there a protocol/registry/statistical analysis plan attached to the trial document?
5_3_No_Information
YES
NO
YES
NO
Methods: Statistical Analysis
Were some of these analysis methods found in the protocol omitted in the clinical trial or were there are additional analysis methods not reported in the protocol? Annotate the sentences that mentions these methods in context of intervention effect assessment.
5_3_No_Information
5_3_No_Good
5_3_Yes_Bad
YES
NO
These sentences might appear around each other.
34
Annotate full sentences if not otherwise specified to annotate only phrases.
Green arrow = which section to find this text evidence

## Slide 35
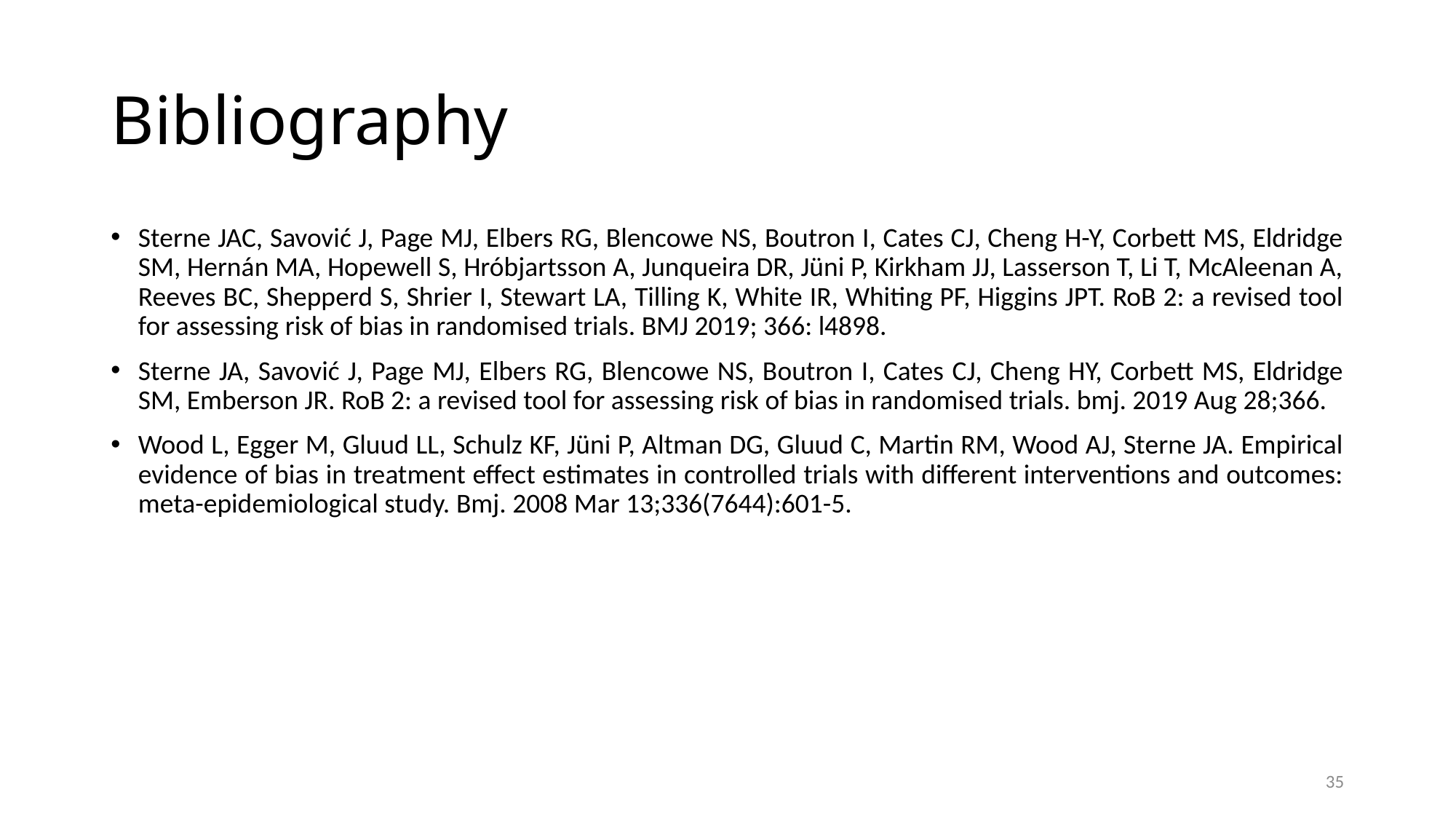

# Bibliography
Sterne JAC, Savović J, Page MJ, Elbers RG, Blencowe NS, Boutron I, Cates CJ, Cheng H-Y, Corbett MS, Eldridge SM, Hernán MA, Hopewell S, Hróbjartsson A, Junqueira DR, Jüni P, Kirkham JJ, Lasserson T, Li T, McAleenan A, Reeves BC, Shepperd S, Shrier I, Stewart LA, Tilling K, White IR, Whiting PF, Higgins JPT. RoB 2: a revised tool for assessing risk of bias in randomised trials. BMJ 2019; 366: l4898.
Sterne JA, Savović J, Page MJ, Elbers RG, Blencowe NS, Boutron I, Cates CJ, Cheng HY, Corbett MS, Eldridge SM, Emberson JR. RoB 2: a revised tool for assessing risk of bias in randomised trials. bmj. 2019 Aug 28;366.
Wood L, Egger M, Gluud LL, Schulz KF, Jüni P, Altman DG, Gluud C, Martin RM, Wood AJ, Sterne JA. Empirical evidence of bias in treatment effect estimates in controlled trials with different interventions and outcomes: meta-epidemiological study. Bmj. 2008 Mar 13;336(7644):601-5.
35
